# Identifying Differential Methylation in Cancer Epigenetics via a Bayesian Functional Regression Model

**DOI:** 10.3390/biom14060639

**Published:** 2024-05-29

**Authors:** Farhad Shokoohi, David A. Stephens, Celia M. T. Greenwood

**Affiliations:** 1 Department of Mathematical Sciences, University of Nevada Las Vegas, Las Vegas, NV 89154, USA; 2 Department of Mathematics and Statistics, McGill University, Montreal, QC H3A 0B9, Canada; david.stephens@mcgill.ca; 3 Lady Davis Institute for Medical Research, Montreal, QC H3T 1E2, Canada; celia.greenwood@mcgill.ca; 4 Gerald Bronfman Department of Oncology, McGill University, Montreal, QC H4A 3T2, Canada; 5 Department of Epidemiology, Biostatistics & Occupational Health, McGill University, Montreal, QC H3A 1G1, Canada

**Keywords:** Gibbs sampler, missing values, bisulfite sequencing, read depth, acute promyelocytic leukemia

## Abstract

DNA methylation plays an essential role in regulating gene activity, modulating disease risk, and determining treatment response. We can obtain insight into methylation patterns at a single-nucleotide level via next-generation sequencing technologies. However, complex features inherent in the data obtained via these technologies pose challenges beyond the typical big data problems. Identifying differentially methylated cytosines (dmc) or regions is one such challenge. We have developed DMCFB, an efficient dmc identification method based on Bayesian functional regression, to tackle these challenges. Using simulations, we establish that DMCFB outperforms current methods and results in better smoothing and efficient imputation. We analyzed a dataset of patients with acute promyelocytic leukemia and control samples. With DMCFB, we discovered many new dmcs and, more importantly, exhibited enhanced consistency of differential methylation within islands and their adjacent shores. Additionally, we detected differential methylation at more of the binding sites of the fused gene involved in this cancer.

## 1. Introduction

DNA methylation at the fifth position in cytosine (5 mC), an epigenetic mark found in many living organisms, has a variety of regulatory roles in disease and normal biology. Specifically, it regulates or causes transcriptional activity during embryonic development [1], tissue development [2], cell differentiation [3], genomic imprinting [4], and X-chromosome inactivation [5], among other things.

We consider, in particular, the role of differential methylation of processes leading to the development of cancer (e.g., [6]). There is increasing evidence that epigenetic variation considerably influences the regulatory processes that modulate cancer growth. The principal focus of our analysis is acute promyelocytic leukemia (apl) [7,8]. apl is an aggressive subtype of acute myeloid leukemia (aml) that accounts for 10% of its cases. aml is a group of hematopoietic neoplasms involving myeloid lineage cells, with apl as a distinct variant. A single mutation is known to cause apl, specifically a translocation involving two genes: *PML* on Chromosome (Chr) 15 and *RARA* on Chr 17 [9], denoted t(15; 17)(q24.1; q21.2); *PML::RARA*. apl leads to high early mortality, often from hemorrhage caused by coagulopathy. Immediate treatment with all-trans retinoic acid is crucial once apl is suspected. Approximately 92% of apl patients have the balanced translocation t(15; 17)(q24.1; q21.1), while 5% have the *PML/RARA* fusion gene from other chromosomal rearrangements or insertions. Although the genetic cause is clear, how epigenetic changes lead to dysregulation is the subject of ongoing research [7]. For example, Schoofs et al. [8] observed broad DNA hypermethylation in apl cells compared with control samples and argued that this is a consequence of a loss of transcription factor binding.

Epigenetic variation is also strongly believed to influence the choice of therapeutic intervention: The success of all-trans retinoic acid treatments as an alternative to chemotherapy has been marked [6], but the treatments vary in efficacy, with resistance to treatment being observed, due to mechanisms related to methylation. Of specific interest is the *PML-RAR*α fusion protein which, when over-expressed due to translocation, has been determined to have a leukemia-generating action [10,11]. Thus, understanding the regulation modulation of the expression of this protein is crucial. Understanding methylation patterns and variations associated with apl can help one to identify potential biomarkers for early diagnosis, prognosis, and treatment targets.

In light of the growing understanding of the role of epigenetic variation in processes influencing tumorigenesis, there is a demand for both fundamental and clinical research on DNA methylation and also a demand for analytical and statistical tools. Several assays to collect methylation data have been developed [12]. Bisulfite sequencing (bs-seq) [13] of DNA is a popular technique that provides positive identification of 5 mC residues in genomic DNA, particularly at cpg sites (where a cytosine nucleotide is followed by a guanine nucleotide in the 5${\;\;}^{\prime }$–3${\;\;}^{\prime }$ direction). bs-seq benefits from the fact that bisulfite treatment will not affect 5 mC but converts unmethylated cytosines to uracils. After polymerase chain reaction and sequencing, the combined experiment leads to single-base resolution information on methylation status by counting the number of times a sequencing read at a single genomic position appears as methylated vs. unmethylated. Coupled with next-generation sequencing (ngs) [14], bs-seq has become an effective tool for obtaining single-nucleotide resolution data from the whole genome. The rapid decline in ngs costs made whole-genome bisulfite sequencing (wgbs) [15] more accessible for research. Other direct assessment techniques include bc-seq [16], bspp [17], and rrbs [3]. rrbs provides single-nucleotide resolution estimates of methylation enriched in genomic regions with high CpG content. bspp is an alternative method for targeting CpG-enriched regions in mammalian genomes [18]. Although bs-seq is one of the most accurate techniques for retrieving methylation data, it has several pitfalls, including false-positive methylation calls due to incomplete conversion, DNA degradation during bisulfite treatment, methylation in pseudogenes, and the inability to discriminate between different methylated states such as 5 mC and 5 hmC [19].

Much research has been conducted to provide efficient methods for quantitative analysis of methylation data. One specific goal is to identify differentially methylated cytosines (dmc) or regions (dmr), as methylation varies based on various biological and epigenetic factors. To capture dmrs, Ref. [20] (BSmooth, bsseq) used a binomial model with a local linear regression to smooth data. Ref. [21] (DSS) utilized a Bayesian hierarchical model (Poisson, Gamma, and log-normal) and the Wald test. Refs. [22,23] (DSS) followed a similar approach with different hierarchical models (Binomial, Beta, and log-normal). Ref. [24] (RADMeth) combined a beta-binomial regression with Stouffer–Liptak tests. Ref. [25] (methylKit) suggested using either logistic regression or Fisher’s exact test. Ref. [26] (BiSeq) used a weighted local likelihood with a triangular kernel assuming binomial probabilities. Ref. [27] (bumphunter) applied linear mixed models on methylation levels with the possibility of adding confounders. Several others (e.g., [28,29,30] (MethPipe), [31] (Bisulfighter), [32] (HMM-DM), and [33] (DMCHMM)) have focused on various hidden Markov models to profile methylation data and to identify dmcs/dmrs. Ref. [34] (WFMM) proposed a wavelet-based functional linear mixed model that accommodates (spatial) correlations across the genome and between samples through functional random effects. See the reviews by [19,35,36,37,38], among others.

Several data features, arising from assays like bs-seq and rrbs, lead to analytic and computational challenges. To illustrate these features, we explore a dataset containing samples from patients with apl and with several different cell types for comparison (Section 2.2). In these data, as in many other sequencing-derived methylation datasets, read depths—the numbers of individual methylated and unmethylated counts recorded via a sequencing platform—vary appreciably and often unsystematically across cytosine positions. cpgs are unevenly distributed [39]; both methylation autocorrelation between samples [40] and between cytosines [41] within a profile change irregularly across chromosomes. High heterogeneity has been observed in DNA methylation in cells from apl patients [8]. Also, a large percentage of positions with missing values exist, and the data magnitude leads to computational challenges for any kind of analysis.

In this work, we propose a new method, called DMCFB, built on a Bayesian functional regression model for the analysis of sequencing-based measures of DNA methylation. Despite a handful of existing methods in DNA methylation data analysis from sequencing experiments, DMCFB is more inclusive and addresses more of the data and computational challenges than any other method, as laid out below.

1. *Missing values and imputation*: Sequencing data often contain many missing values, e.g., in the apl data, 63% of the cpgs have missing values across samples. Almost all methods (except DMCHMM) remove all or most of the cpgs with missing values and then impute the rest. In DMCFB, we set the methylation read and read depth to zero (i.e., y=0,n=0) for missing values in binomial distribution, and we impute methylation level β using the information from *p* neighboring points via a functional regression model. This approach gives a more efficient imputation than other methods.
2. *Read depth*: Many measurements in sequencing data are based on only one or two reads in contrast to a few that have unrealistically high read depths. Several methods (e.g., BiSeq) filter cpgs with low and high read depths, whereas DMCFB keeps all available data and uses the read depth information to adjust their contribution in the model. Since there is a systematic relationship between read depths and methylation levels, i.e., cpgs with high read depth tend to be more hypermethylated (Supplementary Figure S3), DMCFB adds the extra covariate log(n+1) in the model to account for such a relationship.
3. *Raw methylation level vs. counts*: Existing methods model either methylation counts (y,n) or raw methylation levels (or fractions) $\beta^{\mathrm{raw}}=y/n$. Modeling raw data may not fully capture available information (of read depth). In DMCFB, we model (y,n) through a logit(β) link with a Binomial(y;n,β) distribution to account for read depth information.
4. *Transformation*: Those methods that model $\beta^{\mathrm{raw}}$ often tend to use a transformation (e.g., $\log(\beta^{\mathrm{raw}})$ or $\mathrm{logit}(\beta^{\mathrm{raw}})$). DMCFB does not require a transformation of the raw data since it is built on a binomial emission model for the underlying proportion rather than the observed proportion.
5. *Functional pattern*: Methylation proportions are highly correlated across nearby positions and samples. The most efficient methods tend to use smoothing techniques to capture these correlations. These techniques range from weighted local likelihood (e.g., BiSeq) to hmms (e.g., DMCHMM) to functional regressions (e.g., wavelet-based functional linear mixed model in WFMM). Here, DMCFB also builds on functional regression concepts.
6. *Distance between* c*p*g*s*: cpgs are distributed unevenly across the genome, and correlations between methylation levels decrease quickly with distance. DMCHMM and WFMM assume that all positions are equally spaced, which may under/overestimate autocorrelation; however, DMCFB incorporates the distance explicitly.
7. *Sample characteristics*: In addition to these characteristics of the methylation data, existing methods take different approaches to test the association between methylation levels and covariates. Some use a two-stage approach: smoothing each sample and testing the smoothed data (e.g., BiSeq). Others perform two-group comparisons, i.e., testing for differential methylation between cases and controls (e.g., HMMFisher). DMCFB, on the other hand, conducts smoothing and dmc calling in one run.
8. *Biological replicates*: Some methods (e.g., DMRcaller) tend to ignore biological variation across replicates, which may increase type 1 error [38]. Existing methods (e.g., WFMM, BiSeq, and now DMCFB) can utilize such replicates.
9. *Multiple Covariates*: Often, additional features (e.g., clinical information) are collected about subjects. Most widely used methods are not capable of including these covariates in their models. Most of the ones that do are incapable of accounting for multiple covariate types (i.e., categorical, continuous, and combination).

In Section 5, we show that our proposed solutions for these issues collectively create an efficient method for analyzing DNA methylation data which outperforms (in terms of detection capability) other competing methods. We have addressed several computational challenges, including memory management and parallel computation.

We organize the paper as follows. In Section 2, we give brief descriptions of two motivating datasets. In Section 3, we develop a Bayesian functional regression method to identify dmcs. Section 5 covers multiple simulation studies. We analyze both datasets in Section 6 and Section 7. Finally, Section 8 contains discussions.

## 2. Data

We use two different publicly available datasets. One, which we refer to as the wgbs dataset, is a proof-of-principle dataset containing clear signals and is used to develop our method and validate performance through simulations. The second dataset, apl, contains patients with leukemia and will be extensively analyzed using DMCFB, BiSeq, and DMCHMM.

### 2.1. *blk: wgbs* Data on Separated Whole Blood

To develop our method, we used a small part of a publicly available dataset derived from peripheral blood samples in healthy individuals. Cell types were separated, and methylation profiles were estimated with wgbs in cd4${\;\;}^{+}$ tcells (T helper cells), cd14${\;\;}^{+}$ monocytes, and cd19${\;\;}^{+}$ bcells. We extracted data for a small region near the BLK gene on human Chr 8, known to be hypomethylated in b-cells [33,42]. The selected region contains 30,440 cpgs spanning 2 mb (10,352,236–12,422,082). The methylation status of 23.39% of cpgs in these data is missing. We have previously studied this dataset extensively—see [33] and Supplement S1.1. Our choice of a small portion of the blk dataset allowed us to focus on a region known to contain established DMRs of different sizes and magnitudes, providing a thorough benchmark for our analysis. The presence of a reasonable amount of missing data and highly variable read depths, among other challenges, made the dataset particularly suited to testing the robustness of our method under challenging conditions.

### 2.2. *apl: rrbs* Data on Acute Promyelocytic Leukemia

The second dataset that we analyzed was collected from patients with acute promyelocytic leukemia (apl) [8]) and is publicly available on the Gene Expression Omnibus (accession no. GSE42119). These rrbs data contain information on 18 apl patients and 16 control samples, with eight bone marrow samples from patients in remission (rbm), four profiles of healthy cd34${\;\;}^{+}$ cells (cd34), and four profiles from promyelocytes (pmc). Questions of interest include identifying regions where methylation is different among patients versus controls and also looking at different cell types and/or disease status (“active” or “in remission”).

The dataset is challenging to analyze. It includes 9,335,693 cpgs for each of the 34 samples. Nearly 63% of cpgs have missing values across samples. Almost all existing methods will remove either all 63% of the cpgs or may impute a proportion of them. This approach leads to unreliable results due to the loss of much useful information as a result of cpg removal from the analysis. Three out of 16 samples in the control group and 12 out of 18 samples in the apl group are females. Age ranges from 20 to 83 years. On Chr 15, there are 285,437 cpgs available, and 64.27% of cpgs across the samples have at least one missing value. Respectively, 6.19% and 32.31% of cpgs in the control and apl groups have missing values in all samples. On Chr 17, there are 510,386 cpgs available, and among these, 59.81% of the cpgs have at least one missing value, and all samples are missing in 5.55% of cpgs in the control group and 28.91% of cpgs in the apl group—see Supplement S1.2.

A specific focus in our analysis will be the locations of binding sites of the protein *PML-RAR*α, for which Ref. [8] lists some 225 locations on Chr 17. Epigenetic variation in the vicinity of these sites might indicate modulation of the expression of this protein, and hence, detection of differential methylation in these sites may lead to key insights.

## 3. Model

We propose a Bayesian functional regression profiling method to identify dmcs. We describe the main aspects of our method including the introduction of a functional representation of the methylation profiles incorporated into a generalized linear model (glm), the possible statistical inference approaches, several numerical challenges in model fitting, obtained parameters, and the procedure by which a cpg is classified as a dmc.

We regard the methylation counts as realizations of a binomial(n(t),β(t)) process observed on the interval [0,T], which represents some large genomic region, where the read depth {n(t),t∈[0,T]} may vary as the result of the sequencing process or the underlying propensity for methylation and may be platform dependent. In this paper, we condition on the read depth data, thereby ignoring their stochastic properties. We specify that

E[B(t)]=β(t),t∈[0,T],

where B(t)=Y(t)/n(t), and we essentially consider a (functional) model for {B(t)}. We have access to replicate data $\{Y_{i}\left(t\right),t\in \left[0,T\right]\},i=1,\ldots ,m$, and potentially fixed covariates. We construct a functional representation of β(t) using a natural cubic spline basis in *t*; other spline bases may also be used. Our representation allows for further decomposition into group-specific effects when a grouping structure is present.

### 3.1. A Spline Model for Methylation Data

We respect the discrete nature of nucleotide positions and regard the observation locations as positive integers t=1,…,T. We denote the methylation data of a given sample profile by $\left\{\left(y_{t},n_{t}\right),t=1,\ldots ,T\right\}$, where $y_{t}$ and $n_{t}$, respectively, represent the methylation read count and read depth at the *t*th genomic position. A reasonable, if imperfect, model assumes that $y_{t}|n_{t},\beta_{t}∼\mathrm{Binomial}\left(n_{t},\beta_{t}\right),$ for t=1,…,T, where $\beta_{t}$ is the propensity for each site *t* to be methylated at each read instance. In this model, each read is assumed to yield a binary outcome that is conditionally independent of other reads. The methylation level $\beta_{t}$ changes due to different sources of variation. In the blk data, there are three cell types (b cells, t cells, and monocytes). In the apl data, there are four types of samples (cd34, pmc, rbm and apl), as well as the individual’s age and sex. Thus, there is individual-level variation, group variation, and possible variation influenced by additional covariates, and these can be incorporated into a binary regression model

(1) $$y_{t,g,i}|n_{t,g,i},\beta_{t,g,i}∼\mathrm{Binomial}\left(n_{t,g,i},\beta_{t,g,i}\right);g=1,\ldots ,G;i=1,\ldots ,m_{g};t=1,\ldots ,T,$$

where *G* is the number of groups in the variable of interest, $m_{g}$ is the number of samples (individuals) in the gth group, and *T* is the number of cytosines (cpgs) in a sample.

Due to the DNA methylation autocorrelation structure and the unknown functional form of methylation patterns in different regions, we propose a spline-based functional (logistic) regression model. Assume the vector x is formed from a *p*-dimensional (natural cubic) spline basis derived from methylation positions. We then decompose $\beta_{t,g,i}$ in (1) as

$$\begin{array}{c} \mathrm{logit}\left(\beta_{t,g,i}\right)=\log\left\{\beta_{t,g,i}/\left(1-\beta_{t,g,i}\right)\right\}=x\left(\gamma_{0}+\delta_{g}+\upsilon_{g,i}\right), \end{array}$$

where $\gamma_{0}$ represents the baseline group-level parameter, $\delta_{2},\ldots ,\delta_{G}$ are the group-specific contrasts by setting $\delta_{1}\equiv 0$, and $\upsilon_{g,i}$ are the individual-level variations. The $\gamma_{0},\delta_{g},$ and $\upsilon_{g,i}$ are all p×1 vectors. The design matrix for any individual is denoted as $X_{g,i}$ which is a T×3p block matrix. The entire design matrix X that includes all groups (*G*) and all individuals ($M=\sum_{g=1}^{G}m_{g}$) is of dimension MT×(G+M)p. The dimension of the spline basis, *p*, is termed the resolution and is equivalent to a bandwidth parameter. Note that other spline bases will produce fairly similar results. Finally, the model can be augmented to

(2) $$\mathrm{logit}\left(\beta_{t,g,i}\right)=x\left(\gamma_{0}+\delta_{g}+\upsilon_{g,i}\right)+z_{g,i}\eta$$

for some non-positional and non-spline-related fixed effects, where η is a q×1 vector.

### 3.2. Modelling the Impact of Read Depth

We emphasize the importance of adding an extra covariate based on read depth because read depth varies systematically with methylation levels and because higher read depths tend to result in larger methylation levels (Supplementary Figure S3). For instance, a plausible model for the blk data is given as

(3) $$\mathrm{logit}\left(\beta_{t,g,i}\right)=x\left(\gamma_{0}+\delta_{g}+\upsilon_{g,i}\right)+\eta_{1}\log\left(n_{t,g,i}+1\right)$$

which allows for the effect of read depth in an additive fashion on the linear predictor scale. We treat read depth as a deterministic quantity and perform a conditional analysis, even though the stochastic properties of read depth might also be of interest in other settings.

## 4. Bayesian Inference

We first consider maximum likelihood (ml) estimation and then a Bayesian approach. ml estimates are useful in full Bayesian analysis as they allow for the Bayesian computational strategy to be implemented more efficiently by providing initial estimates for the Bayesian procedure. The log-likelihood function is

$$\ell \left(\Psi \right)=\sum_{g=1}^{G}\sum_{i=1}^{m_{g}}\sum_{t=1}^{T}y_{t,g,i}\left[x\left(\gamma_{0}+\delta_{g}+\upsilon_{g,i}\right)+z_{g,i}\eta \right]-n_{t,g,i}\log\left(1+e^{x\left(\gamma_{0}+\delta_{g}+\upsilon_{g,i}\right)+z_{g,i}\eta }\right),$$

where $\Psi =(\gamma_{0},\delta_{2},\ldots ,\delta_{G},\beta_{1,1},\ldots ,\beta_{G,m_{G}},\eta )$ and $\beta_{t,g,i}/\left(1-\beta_{t,g,i}\right)=e^{x\left(\gamma_{0}+\delta_{g}+\upsilon_{g,i}\right)+z_{g,i}\eta }.$

One can derive a numerical solution $\hat{\Psi }=\arg\max_{\Psi }\ell \left(\Psi \right)$ for the ml estimates; however, the process involves solving a system of nonlinear equations and is computationally burdensome. As is usual in glms, one can adopt an iteratively re-weighted least squares (irls). Due to high-dimensionality, there are typically memory capacity issues; however, one can exploit the block-structure of the design matrix to speed up the process, or one can resort to minorization–maximization (mm) or quasi-irls, which simplifies the iterative estimation procedure. These procedures are appreciably faster to compute but can be slower to converge to mle. We prefer a fully Bayesian approach, as it yields a more complete representation of associated uncertainties in light of the data and model specification.

### 4.1. Bayesian Model

Denote the estimated logistic scale methylation levels by $\beta^{*}=\left[\beta_{t,g,i}\right]$. We propose the full Bayesian model characterized by the factorization

(4) $$p\left(y|n,\beta^{*}\right)p\left(\beta^{*}|x,z,\gamma_{0},\delta_{g},\upsilon_{g,i},\eta ,n\right)p\left(\gamma_{0},\delta_{g},\upsilon_{g,i},\eta |n\right).$$

We allow for a deterministic relationship between $\left(x,z,\gamma_{0},\delta_{g},\upsilon_{g,i},\eta \right)$ and $\beta^{*}$, specifically that at each site, $\mathrm{logit}(\beta_{t,g,i})$ follows the linear model in (2). By choosing suitable priors, inference for the collection of unobserved quantities $\left(\gamma_{0},\delta_{g},\upsilon_{g,i},\eta \right)$, and $\beta^{*}$ is attained using computational methods based on Markov chain Monte Carlo (mcmc). For full Bayesian inference, we perform block updates of the effect-specific parameters. We devise a Gibbs sampler algorithm by introducing independent Gaussian priors $N(0,\tau_{\theta }^{2})$ on the model parameters, although this can be easily relaxed [43]. The hyper-parameter $\tau_{\theta }$ can be specified subjectively, but it is also common to adopt an empirical Bayes approach and choose $\tau_{\theta }$ after inspection of the data. In Section 5.1.5, we study the effect of priors having common $\tau_{\theta }$ versus InverseGamma(a,b) on $\tau_{\theta_{j}}$, where (a,b) are estimated empirically.

The Gibbs sampler updates are structured as block updates for the term-specific parameters, $\gamma_{0},\delta_{g},\upsilon_{g,i},\eta$, conditional on the other parameters. In each update, full conditional posterior distribution corresponds to a posterior from a Bayesian binary regression model. There are several possible strategies for a full update for one parameter block:

1. Multiple ‘inner’ accept–reject Metropolis–Hastings steps to sample the full conditional posterior giving an exact block update leading to faster convergence in the ‘outer’ chain;
2. Single ‘inner’ accept–reject Metropolis–Hastings steps to sample the full conditional posterior, giving an exact block update but possibly leading to slower convergence in the ‘outer’ chain;
3. Gaussian data augmentation samplers [44]; these methods can be useful for logistic and probit models and for models based on scale mixtures of Gaussians, but for large data sets, they involve the introduction of a considerable number of augmenting variables that require storage and sampling;
4. An exact update using the approximate full conditional distribution given by the Normal approximation to the non-Normal exact version, i.e., from the standard approximation $N(\hat{\theta },{\left(X^{⊤}W\left(\hat{\theta }\right)X\right)}^{-1})$ inspired by the quadratic approximation of log-likelihood.

The fourth approach appeals to a Gaussian approximation of the full posterior distribution, and we have found that the Gaussian approximation works well in most cases. The Gibbs sampler approach—rather than a joint update of all parameters simultaneously—is usually more feasible in large-scale problems. Finally, it may not feasible to build a complete functional regression genome-wide in our method. Instead, we use a partitioning approach that fits the model in smaller genomic segments and which is tailored to the computational resources available. Alternative approaches for genome-wide analysis without partitioning are variational Bayes, which constructs an approximate Bayes solution based on a tractable approximating model, or Hamiltonian mcmc, which introduces further augmenting variables and exploits the energy configuration (of the log joint posterior) in the extended space. Our Gibbs sampler algorithm is given in Algorithm 1.
**Algorithm 1:** Gibbs Sampler Algorithm in DMCFB
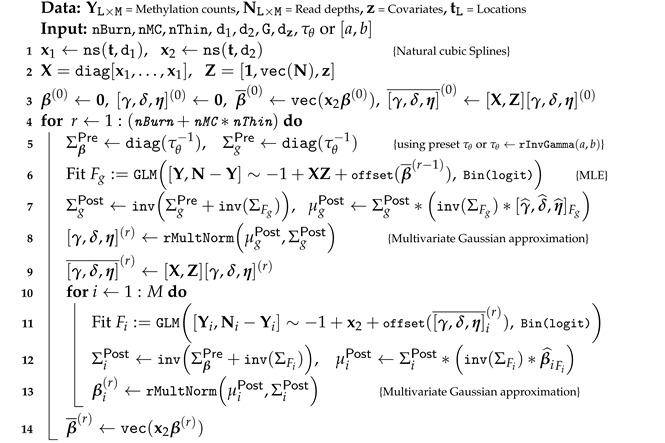


### 4.2. Identifying *dmc*s

Having mcmc samples for parameters at cpg *t*, we compute the 100(1−α)% credible interval for each (multiple) comparison(s) in the categorical variable of interest. If at least one of the intervals does not contain zero at cpg *t*, the position is classified as a dmc. The process is undertaken for all cpgs. In addition to (overall) dmc, the pairwise dmcs are also reported when there are more than two categories in the variable of interest. An alternative approach to detect dmc is to use global Bayesian *p*-values [45] or Bayesian fdrs [46]. See Section 8 for a discussion and comparison.

## 5. Simulation Study

We have assessed the performance of DMCFB in simulations. We carry out four simulation studies. The first uses the scenarios from [33] which are inspired by the cell-separated data described in Section 2.1, and the remaining three include, in Section 5.2, a study adapted from [47], in Section 5.3, a simulation inspired by data generated from hmm models, and in Section 5.4, a study adapted from [27]. In simulation studies, we set α=0.05 and collect a set of 10,000 mcmc samples thinning by taking one in every 10 samples after a burn-in of 5000 iterations.

### 5.1. Simulation Study Example 1

#### 5.1.1. Scenarios and Settings in Example 1

We use identical scenarios and settings as the simulation proposed in [33]. The methylation information available in the bc and monocyte samples were chosen to generate simulated data as follows:

1. First, the read depths the methylation counts of all bc samples were aggregated.
2. Next, the missing information was imputed using nearby count information.
3. Using ‘lowess’ in R, we fitted a smooth curve (span =0.05) denoted g1; this curve was used to specify a baseline profile.
4. Using Supplementary Table S2, we generated the second group curve called g2 by adding effect sizes to the chosen regions of g1.
5. By fitting a Normal distribution to the methylation difference between g1 and all bc samples, we obtained μ≈0 and σ≈0.18. We used these estimates to add extra variation to g1 and g2 for all samples.
6. Read depths of all bc and monocyte samples were chosen to generate R=500 simulated datasets; within each dataset, we generated 8 samples from g1 and 13 samples from g2 by completing the following steps:
  (a) Additional variation at each site was generated using N(0,0.18) random errors, which were added to the methylation levels in g1 and g2.
  (b) All values are truncated to fall in [0,1].
  (c) Methylation counts were generated by multiplying the generated methylation levels in the previous step. The integer parts of these data were chosen as the final methylation counts.

Supplementary Figure S4 presents the graph of the dmrs, a simulated dataset that compares it to the methylation in the real dataset.

We chose several prominent and widely used tools for comparison, including bsseq [20], bumphunter [27], BiSeq [26], DSS [23], DMRcaller [48], HMM-DM [32], HMM-Fisher [49], WFMM [34] and DMCHMM (non-weighted and weighted approaches) [33]—see Supplement S3 for a comparison. Selected results/figures are shown in the main paper, and full results/figures are in Supplements.

#### 5.1.2. Simulation Results in Example 1

Quantification of the performance of each method is based on the following definitions: true positive (tp, cpg is correctly identified as dmc); true negative (tn, cpg is correctly identified as non-dmc (ndmc)); false positive (fp, cpg is incorrectly identified as dmc); false negative (fn, cpg is incorrectly identified as ndmc). The ‘sensitivity’ (se) of dmrs and ‘specificity’ (sp) of ndmrs for the *r*th simulated dataset are defined as

$${\mathrm{SE}}_{j}\left(r\right)=\frac{\#\mathrm{TP}\;\mathrm{in}\;\mathrm{the}\;j\mathrm{th}\;\mathrm{DMR}\;}{\#\mathrm{CpGs}\;\mathrm{in}\;\mathrm{the}\;j\mathrm{th}\;\mathrm{DMR}},\;\;\mathrm{and}\;\;{\mathrm{SP}}_{k}\left(r\right)=\frac{\#\mathrm{TN}\;\mathrm{in}\;\mathrm{the}\;k\mathrm{th}\;\mathrm{NDMR}}{\#\mathrm{CpGs}\;\mathrm{in}\;\mathrm{the}\;k\mathrm{th}\;\mathrm{NDMR}},$$

j=1,…,10, k=1,…,11, and r=1,…,R=500. The average $SE_{j}$ of the jth dmr and the average ‘specificity’ of the kth ndmr are respectively calculated as ${\mathrm{SE}}_{j}=\sum_{r=1}^{R}{\mathrm{SE}}_{j}\left(r\right)/R$ and ${\mathrm{SP}}_{k}=\sum_{r=1}^{R}{\mathrm{SP}}_{k}\left(r\right)/R$. The accuracy is calculated as ACC={#TP+#TN}/#CpGs for each method. We also report the modified accuracy (macc) by assuming that dmr1 through dmr8 are the only real dmrs as given in [33].

We set the nominal false discovery (fdr) threshold at 5% for any applicable method. We modified the default parameters in each tool to match simulation settings and to increase their efficiency. WFMM does not impute missing data; instead, we used a naive approach based on neighboring information to impute them in the simulated datasets so as to obtain higher performance. In DMCFB, we choose normal priors, p=30, and α=0.05.

**Figure 1:**
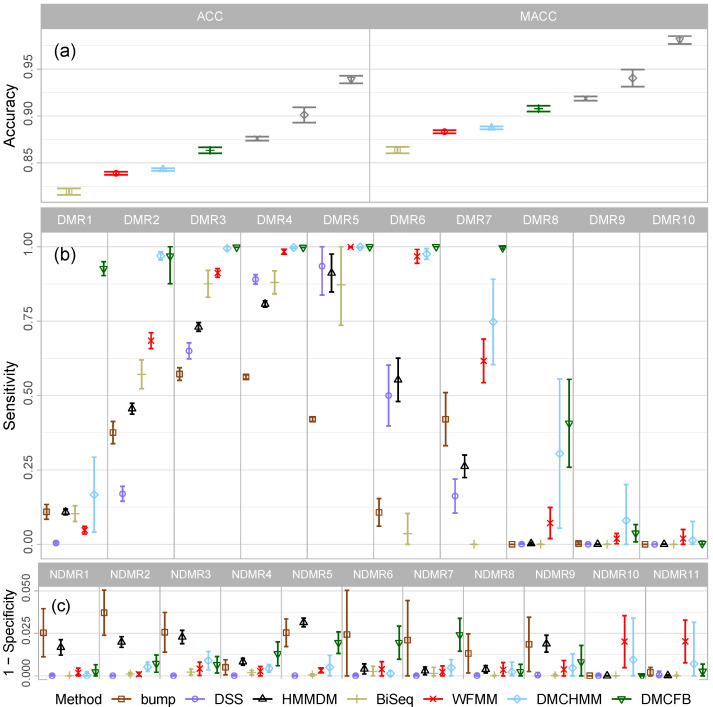
(**a**) Average accuracy (acc) and modified accuracy (macc) in Scenario 1 of Example 1; (**b**) average proportion of correctly identified dmcs (‘sensitivity’) and (**c**) incorrectly identified dmcs (‘1-specificity’) in Scenario 1 of Example 1, separated by dmrs and ndmrs in simulated data; errors generated from N(0,0.18). Relevant results are reproduced from [33]. (sd error bars are added).

Figure 1a depicts the acc and macc results for different methods. DMCFB performs better than the existing methods. Figure 1b,c illustrates the average ${\mathrm{SE}}_{j}$ and the average $1-{\mathrm{SP}}_{k}$, separated for each dmr and ndmr. DMCFB is superior in terms of ${\mathrm{SE}}_{j}$. The results for $1-{\mathrm{SP}}_{k}$ show that DMCFB is either better than or comparable to other methods. One advantage of DMCHMM is the ability to detect small differences in methylation profiles (dmr1, dmr8) and large differences with high precision (dmr2-7) [33]. Our proposed method outperforms DMCHMM in this regard. Supplementary Figure S10 presents Cohen’s Kappa. Our method outperforms the competing methods. Supplementary Figure S11 shows the proportions of the times that the start and end of a dmr are identified as dmcs. DMCFB’s results are higher than those of other methods. The empirical fdr (efdr) in Supplementary Figure S12 shows that DMCFB attains the nominal fdr.

#### 5.1.3. Sensitivity Analysis in Example 1

**Figure 2:**
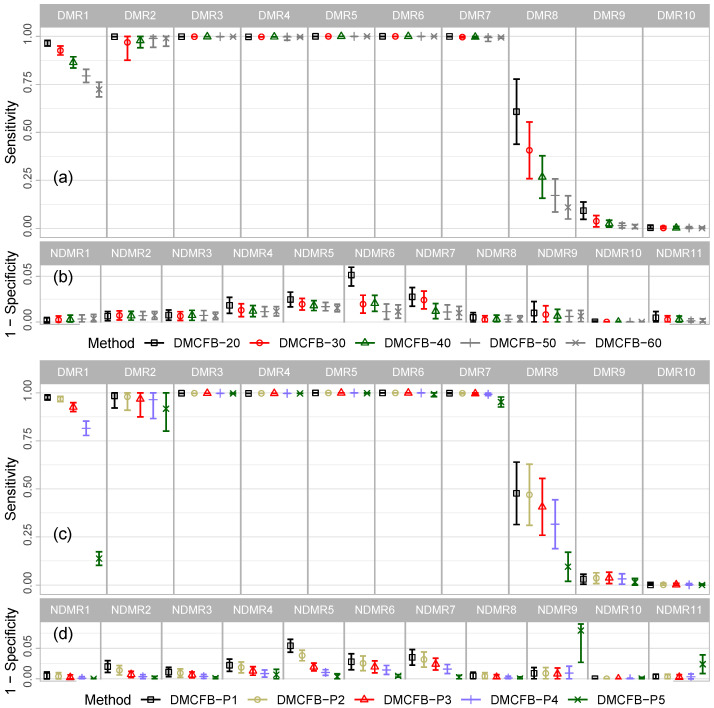
(**a**) Average proportion of correctly identified dmcs (‘sensitivity’) and (**b**) incorrectly identified dmcs (‘1-specificity’), and (**c**) average proportion of correctly identified dmcs and (**d**) incorrectly identified dmcs for each choice of bandwidth. Results are separated by dmrs and ndmrs in simulated data for Example 1 Scenario 1 using DMCFB. Errors are generated from N(0,0.18). (sd error bars are added).

Since our proposed Bayesian approach depends on the choices of bandwidth and prior, we performed sensitivity analyses concerning both specifications. For bandwidth, we have chosen a wide range of values in {20,25,…,60} to study its impact. Figure 2a,b compares ${\mathrm{SE}}_{j}$ and $1-{\mathrm{SP}}_{k}$ of DMCFB using different bandwidth lengths. From this figure and Supplementary Figures S23–S26, we conclude that the results are fairly robust for the bandwidth selection, specifically when the difference in methylation levels is large. Next, concerning prior precision, we examine Gaussian priors with different precisions, $\tau_{\theta }^{-1}\in \left\{30,20,10,1/0.18,1,0.3,0.2,0.1\right\}$, for sensitivity analysis. It transpires that, overall, better results are observed with respect to efdr if the precision is estimated from the data via empirical Bayes. In this case, the average empirical site-specific standard deviation proves to provide a reasonable scaling, leading to the specification $\tau_{\theta }=0.18$. Figure 2c,d and Supplementary Figures S32–S35 show the robustness of the results. An alternative approach is to use prior $p(\tau_{\theta })$ and estimate its parameters empirically (Section 5.1.5).

A summary of the conclusions drawn from several simulation studies is as follows:

1. DMCFB outperforms all considered methods in almost all criteria including accuracy, Cohen’s Kappa, ${\mathrm{SE}}_{j}$, and the percentage of detecting start and end of dmrs.
2. In terms of $1-sp_{k}$, the DMCFB method is either comparable or better than that of other methods.
3. Apart from bumphunter, HMMDM, and BiSeq, all other methods including DMCFB attained the nominal fdr at 5%.
4. Similar to DMCHMM, DMCFB’s performance is excellent in dmrs with large effect sizes (say 0.2) and does not depend on any other features.
5. For small effect sizes (say 0.1), our method’s performance is superior to other methods’.
6. DMCFB does a better job in smoothing the data, followed by DMCHMM and WFMM. Specifically, DMCFB is robust concerning the length of dmrs and the size differences; see dmrs 2–7.
7. Similar results are observed using larger variability for noise (σ=0.24) (see Supplement S4.3). The decline in performance using our method is negligible. This result shows that DMCFB is robust with regard to variation in the data, while other methods have noticeable performance loss.
8. Using a different simulation setting with dmrs of different lengths, we observed trends similar to those described above (see Supplement S4.4).
9. Sensitivity analysis shows that DMCFB is robust for bandwidth selection.
10. A sensitivity analysis is conducted by re-assembling regions to see how a regulatory landscape is altered by the grouping factor. The results are observed to be robust (see Supplement S5.4).
11. Sensitivity analysis concerning the precision of prior distribution shows the robustness of the results. More specifically, better efdr is observed when the prior precision is estimated from data using an empirical Bayes approach.

#### 5.1.4. Missing Value Imputation

**Figure 3:**
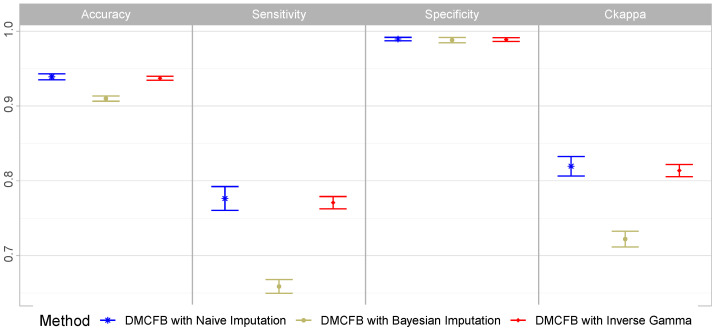
Comparing DMCFB with three settings: Naive imputation with a common hyperparameter, Bayesian imputation with a common hyperparameter, and Naive imputation with inverse-gamma priors on hyperparameters.

Missing value imputation in this type of methylation data demands extra caution due to high missingness (e.g., 63% in the apl data) and low read depth. A common Bayesian imputation technique is to use the full conditional posterior $p(y_{\mathrm{mis}},n_{\mathrm{mis}}|y_{-\mathrm{mis}},n_{-\mathrm{mis}},\beta^{*})$ (or for non-stochastic read depths $p(y_{\mathrm{mis}}|y_{-\mathrm{mis}},n,\beta^{*})$). However, here, the missingness is of a different nature to conventional missing outcome data; in almost all cases, the missingness is due to an absence of any reads at a given location. To this end, we propose a simple yet efficient method to impute missing values by setting (y=0,n=0) for missing values and predicting $\beta^{*}$. Such a method does not require read depth imputation, and the log-likelihood of missing data becomes zero, playing no role in total log-likelihood. A simulation (Figure 3, for Example 1) shows that our method (blue color) has substantially better performance over Bayesian imputation (khaki color). In addition, our method is faster.

#### 5.1.5. Choice of Priors

The choice of priors is of prime importance. In general, it is straightforward using simulation to study the impact of different prior specifications; elicitation can be readily carried out by simulation from the prior and then reconstructing the prior for the profiles by computing the β values in (3). To expedite the computations, we use conjugate priors and estimate the common uncertainty empirically. This assumption can be easily relaxed by using non-informative priors [43], although we do not recommend the use of improper priors. In a simulation study (Figure 3), we used inverse-gamma priors $p(\tau_{\theta_{j}};a,b)$ and estimated the hyperparameters (a,b) empirically. We observe that a conjugate prior with common uncertainty (blue color) gives a similar performance compared with inverse gamma (red color) but is slightly faster.

### 5.2. Simulation Study Example 2

In the second simulation study, different from Example 1, we adopted the simulation setup in [47]. To this end, we used 5000 genomic positions and created two groups each of 10 subjects. We then generated 50 dmrs for each of the R=100 simulated datasets. As stated in [47], a dmr is constructed by sampling a cluster of neighboring cpgs and simulating the number of methylated reads, conditional on observed coverage, for the samples from one population from a binomial distribution. The binomial probabilities are equal to the observed methylation proportions plus or minus an effect size that is randomly sampled from a distribution that represents small to moderate effect sizes (ranging from approximately 0.1 to 0.5).

**Figure 4:**
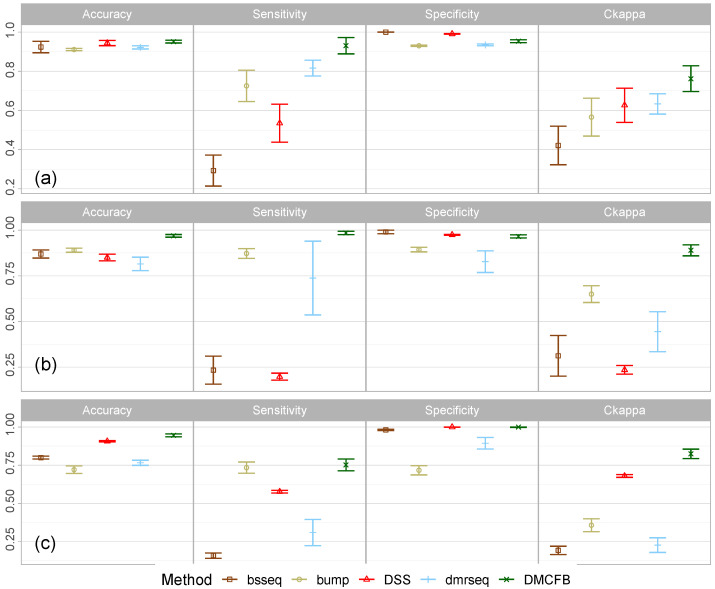
The performance of different methods in simulation study Example 2 (**a**), Example 3 (**b**), and Example 4 (**c**).

Since the lengths and locations of the dmrs were different from each other in each simulated dataset, we only calculated the overall accuracy, sensitivity, specificity, and CKappa. Due to crashing, we were not able to run the simulations for BiSeq and DMCHMM. We further added dmrseq to the methods for comparisons. We depicted the results in Figure 4a. From this figure, we observe that our proposed method outperforms all the rival methods. DMCFB was the only method with both sensitivity and specificity above 95%. Note that we run some other simulation settings using a similar approach, and in some of them, the performance of all methods was slightly lower, but in all of them, our method outperformed all other methods. Note that we changed the default options in bumphunter to increase its efficiency.

### 5.3. Simulation Study Example 3

In the third simulation study, different from Examples 1 and 2, we simulate 5000 positions from an hmm model with eight states spread over [0,1] and binomial emissions, where the read depths are generated from uniform [15,45] (or Poisson (λ=30)). This pattern represents Group 1 or baseline. We generate 10 random samples (subjects). We do the same for Group 2 except that we randomly select four regions of random sizes between [100,300] and add methylation differences {0.2,−0.2,0.3,−0.3}. These regions represent dmrs. In total, we simulate R=100 datasets and run the analysis. We present the results in Figure 4b. We can observe that DMCFB is again the dominant method.

### 5.4. Simulation Study Example 4

We follow the settings in Ref. [27]. Note that the settings are designed for microarray data and only simulate the logit of β values. Thus, we modified the code to generate single nucleotide data and used the read depths from the blk data. We set the parameters of the simulation as follows: the number of bumps as 10, the group size as 200, the number of probes in each sample as 5000, the parameter of bumpmaker as 0.3, the correlation structure as AR(1;ϕ=0.21,σ=0.2), and the outlier distribution as $t_{5}$. To this end, we simulate eight samples (representing b cells) as the baseline group and 13 samples (representing monocytes) as Group 2 having bumps with random sizes and effects and having random locations. In total, we generated R=100 datasets and presented the results in Figure 4c. Again, DMCFB outperforms other methods. It has the perfect specificity with the highest sensitivity, accuracy, and CKappa values compared to the other methods. Note that we set the smoothing to FALSE in DSS to increase its efficiency in both Examples 3 and 4.

## 6. Proof of Principle Analysis of Sorted Cells

The blk data resembles a dataset in which several challenges such as multiple groups, variable read depths, and missing values exist jointly. Almost none of the existing methods can efficiently handle such complexity by addressing all the known challenges. To this end, we have compared the methylation profiles between cell types near *BLK* described in Section 2.1 via DMCFB. The blk data have 8 bc, 13 monocyte, and 19 tc samples in which methylation counts and read depths for 30,440 cpgs are available. We use normal priors in which $\tau_{\theta }\approx 0.18$ is computed after inspecting the data. The bandwidth is set to 30. The fitted model is identical to (3), where bc is the baseline and $n_{t,g,i}$ is the read depth at position *t* of sample *i* in group *g* where t=1,…,T=30,440, g=1,2,3 and $i=1,\ldots ,m_{g}$ with $m_{1}=8,m_{2}=13$ and $m_{3}=19$.

To achieve genome-wide significance in genetics, the thresholds of $\alpha =10^{-5}$ and $\alpha =10^{-8}$ are commonly used [50]. Appropriate thresholds for epigenetic data such as methylation will depend on the platform, the coverage of the genome, and the correlations between signals across the genome. Although an appropriate threshold for wgbs data is not known, for real data analyses in this paper, we set $\alpha =0.05,10^{-5}$, and $10^{-8}$ and collect a set of 50,000 mcmc samples, thinning the output by taking one in every 10 samples, after a burn-in of 10,000 iterations.

We depict the trace plots of one parameter in the model in Supplementary Figure S44 for five selected cpgs, which shows that the convergence occurred. Similarly, all other parameters of all positions converged quickly. We constructed the credible intervals for each α (Supplementary Table S5). A cpg site is classified as a dmc if at least one of the (1−α)% credible intervals for group comparisons does not include zero.

**Figure 5:**
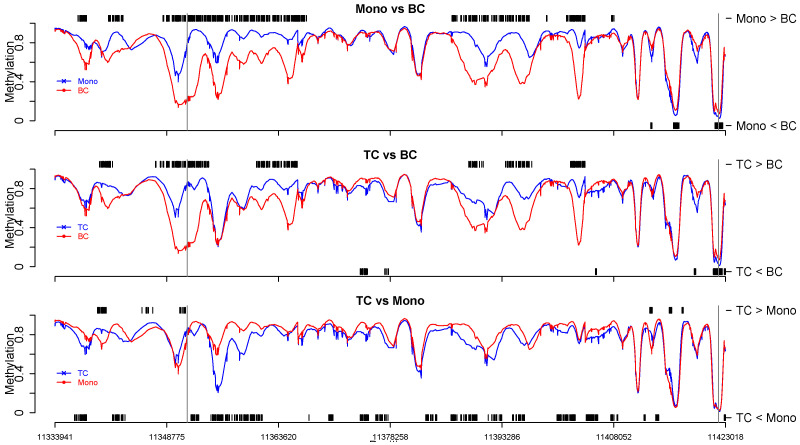
blk data. Identified dmcs for three pairwise comparisons of cell-type-specific methylation data near the *BLK* gene promoter. Short black vertical lines indicate cpgs where one cell type was significantly different from the other at credible interval level $\alpha =10^{-8}$. The average methylation level for each cell type is plotted. Long vertical lines are the start and end of the *BLK* gene.

For $\alpha =10^{-8}$, we observed, in total, 28.6% of cpgs were identified as dmc (Supplementary Table S5). Among these, approximately 13.6%, 11.4%, and 18.5% of cpgs were dmcs, respectively, for pairwise comparisons bc vs. monocyte, bc vs. tc, and monocyte vs. tc. Figure 5 depicts the analysis results for the region near the *BLK* promoter, where we can observe that the three cell types are differentially methylated specifically at the *BLK* promoter. The results are in concordance with the literature [42]. It is worth noting that most analytical tools are incapable of imputing missing values efficiently and analyzing all three groups in the blk data at once, i.e., performing three simultaneous comparisons.

## 7. Analysis of Patients with Acute Promyelocytic Leukemia vs. Control Cell Types

Ref. [8] described differences in methylation between patients with apl and three types of normal cells: mononuclear cells from remission bone marrow, cd34${\;\;}^{+}$ cells from healthy donors, and promyelocytes derived in vitro from cd34${\;\;}^{+}$ cells. apl is known to be caused by a Chr 15 and Chr 17 translocation affecting promyelocytic leukemia-retinoic acid receptor α, and these authors explored, in rrbs data, how methylation patterns differed across the genome. Using rrbs profiling, Ref. [8] showed widespread hypermethylation in apl samples, not only restricted to the location of the translocation.

We use DMCFB to reanalyze the data on Chr 15 and 17 to see if additional sensitivity can be achieved. From the point of view of building an analytic approach for these data, the apl data present several significant challenges:

1. A large proportion of the data is missing. Most methods cannot handle such complexity and will remove either all or big chunks of positions.
2. Additional covariates such as age and sex are of interest, and this may force users to choose among a few methods that can handle multiple covariates.
3. Read depths vary dramatically in different regions; few methods account for such information.
4. It can be seen from Supplementary Figure S2 that the methylation pattern changes dramatically in different regions; simple smoothing techniques cannot efficiently capture such complexity.

We can conclude that the approaches that are taken in [8] (i.e., removal of most positions with missing values and low read depth and not including additional sources of variation in age and sex) have probably led to a set of weak or unreliable results. Hence, we reanalyzed the apl dataset using DMCFB and compared the results with those of BiSeq.

We conduct two reanalyses of the data. First, we perform a simple case–control comparison without considering any additional covariates and grouping the three types of controls (cd34, pmc, and rbm) together. This allows for a direct comparison of the results from DMCFB with the results from BiSeq presented by [8] (Section 7.1). The second analysis compares the four groups of samples and includes covariates (Section 7.2).

### 7.1. Reanalysis of the *apl* Data by Comparing DMCFB and BiSeq

The model

$$\mathrm{logit}\left(\beta_{t,g,i}\right)=x\left(\gamma_{0}+\delta_{g}+\upsilon_{g,i}\right)+\eta_{1}\log\left(n_{t,g,i}+1\right),$$

is used in DMCFB, where $\gamma_{0}$ is the baseline group level (control); $\delta_{2}$ (apl) is the group-specific contrast by setting; $\delta_{1}\equiv 0$, $v_{g,i}$ are the individual-level variations; x is the (natural cubic spline transformation) design matrix; $n_{t,g,i}$ is the read depth at position *t* of individual *i* in the group *g* for t=1,…,T (T=285,437 and 510,386 for Chr 15 and 17, respectively); g=1,2; and $i=1,\ldots ,m_{g}$ with $m_{1}=16$ control samples and $m_{2}=18$ apl patients.

We used normal priors in which $\tau_{\theta }\approx 0.12$ after inspecting the data. We depict the trace plots of one parameter in the model in Supplementary Figure S45 for five selected cpgs in Chr 17, which shows that the convergence occurred. Similarly, all other parameters of all positions converged quickly. Here, we choose a threshold of $\alpha =10^{-8}$ and note that very similar numbers of dmcs were identified if we used the more liberal threshold. For BiSeq, we first set $\alpha =10^{-8}$, but it led to only a very small number of dmcs. Therefore, in BiSeq, we set α=0.1 and 0.05 for testing and trimming clusters, respectively.

Table 1 (A, B) shows how and when DMCFB and BiSeq agree or disagree for Chrs 15 and 17, respectively. The tables show the numbers of dmcs called for the two methods along with the percentages conditional on an annotation (islands, shores, and deserts). First, in Table 1 (A), we observe that a much smaller number of sites are called dmc by BiSeq relative to DMCFB. The significance thresholds used for BiSeq will affect this proportion, but the level we have chosen is already quite liberal (α=0.05). Second, we can see that if BiSeq calls a site a dmc, then DMCFB will call the same site a dmc 76.1% of the time. This proportion is fairly consistent across the three site annotations, although it is a little lower for desert regions than for islands and shores. In contrast, we can see substantial differences in performance among the sites that BiSeq does not call dmcs. Overall, the DMCFB method calls 27.49% of these sites dmcs. However, this percentage ranges from 25.6% for cpg deserts to 44.5% for the cpg islands. In Table 1 (B), similar findings are obtained. The agreement with BiSeq calls is a little higher—80.89% instead of 76.1%—and a similar trend is seen among sites not called by BiSeq, such that DMCFB is calling more dmcs in islands and shores than in desert regions. Supplementary Figures S50 and S51 look at coherence within islands. If an island is identified as differentially methylated by DMCFB, then it is more likely that most of the cpgs inside the island are identified as dmc compared to BiSeq, and this finding indicates better smoothing by DMCFB, as we saw in simulation studies.

We now address the detection of differential methylation in the vicinity of *PML-RAR*α binding sites. Supplementary Table S8 presents which of the known binding sites listed in Ref. [51] are dmc, when comparing apl and controls, by either method. We observe that DMCFB identified more binding sites as dmc than did BiSeq. On Chr 15, DMCFB identified 12 sites, while BiSeq identified only 2 sites as dmc; on Chr 17, DMCFB identified 33 sites, while BiSeq identified only 2 sites. We also display the read depth of the known binding sites that are not dmc by either method or identified by only DMCFB in Supplementary Figures S46A,B on Chr 15 and S47A,B on Chr 17. From these figures, we observe that when neither method detects a dmc (at known binding sites), often the read depth is very low, and so the capability to detect dmcs is compromised. However, when the read depth is moderate or high, DMCFB identifies appreciably more binding sites as dmcs. In addition, DMCFB exhibits more sensitivity for capturing the documented widespread dysregulation.

Additional results are found in Supplement S8.1. Some highlights are:

- Supplementary Table S9 examines the agreement between adjacent islands and shores and shows whether they agree. On both analyzed chromosomes, if an island contains at least one dmc, its adjacent shore is more likely to be identified as differentially methylated by DMCFB than BiSeq, suggesting increased sensitivity of DMCFB. For example, on Chr 15 with DMCFB, 56.0% of sites with at least one dmc in an island also identified at least one dmc in the adjacent shore. In contrast, for BiSeq, this percentage was only 32.6%. Similarly, if an island was not differentially methylated, its adjacent shore was also more likely to be not called differentially methylated by DMCFB than BiSeq.
- Supplementary Table S10 addresses the direction of the methylation changes overall and by annotation. When both methods identify a cpg as dmc, they mostly agree on the direction of the methylation, and if they do not agree on the direction, BiSeq tends toward hypermethylation. If a cpg site is detected as dmc by only DMCFB, the direction is mostly hypomethylation in islands and hypermethylation in shores and deserts. If a cpg site is detected as dmc by only BiSeq, the direction is mostly hypomethylation in both Islands and Shores. These findings are similar for both Chrs 15 and 17.
- Supplementary Figures S48 and S49 represent read depth for dmcs captured by DMCFB, BiSeq, or both. Clearly, the median read depth is lower for DMCFB, which speaks to our method’s efficient use of information. When both methods call a dmc, read depth tends to be very high in the apl samples, i.e., a strong clear signal for differential methylation.
- Supplementary Figures S52–S55 illustrate the difference between the raw and smoothed data and show how apl’s methylation levels are often much higher than those among controls. The differences between patients and controls become far more apparent after smoothing, and overall, DMCFB captures far more sites as differentially methylated than BiSeq.

We present a comparison between DMCFB and DMCHMM in Supplement S8.3.

### 7.2. Reanalysis of *apl* Data by Accounting for All the Information Using DMCFB

In this section, we reanalyze the apl data using DMCFB but account for the additional information of sex, age, and the four groups (cd34, pmc, rbm, and apl). The full model is set to

$$\mathrm{logit}\left(\beta_{t,g,i}\right)=x\left(\gamma_{0}+\delta_{g}+\upsilon_{g,i}\right)+\eta_{1}\log\left(n_{t,g,i}+1\right)+\eta_{2}z_{g,i}+\eta_{3}w_{g,i},$$

where $\gamma_{0}$ is the baseline group level (cd34); $\delta_{1}\equiv 0$, $\delta_{2}$ (pmc), $\delta_{3}$ (rbm), and $\delta_{4}$ (apl) are the group-specific contrasts; $v_{g,i}$ are the individual-level variations; x is the (natural cubic spline transformation) design matrix; $n_{t,g,i}$ is the read depth at position *t* of individual *i* in group *g* with its effect $\eta_{1}$; $z_{g,i}$ is the sex of individual *i* in group *g* with its effect $\eta_{2}$; and $w_{g,i}$ is the age of individual *i* in group *g* with its effect $\eta_{3}$. Recall t=1,…,T (T= 285,437 and 510,386 for Chr 15 and 17, respectively), g=1,…,4 and $i=1,\ldots ,m_{g}$ with $m_{1}=m_{2}=4$, $m_{3}=8$, and $m_{4}=18$ for the four groups cd34, pmc, rbm, and apl, respectively.

We set $\alpha =0.05,10^{-5}$ and $10^{-8}$ in constructing the credible intervals. Table 2 shows the results for Chr 15 and 17. The patterns in both Chrs and all three α levels are almost similar, specifically for $\alpha =10^{-5}$ and $\alpha =10^{-8}$. This analysis leads to six age- and sex-adjusted pairwise comparisons across the four sample types. The greatest number of dmcs are found for the contrast apl vs. cd34. Overall, about 5% of cpgs were identified as dmc using α=0.05.

It is worth noting that almost all of the analytical tools are incapable of analyzing the apl data by comparing all six contrasts in one run and considering all additional information on subjects while keeping all the cpgs in the analysis and imputing the missing values.

## 8. Discussion

We have proposed an efficient analysis method, DMCFB, for the identification of epigenetic features including dmc identification based on a functional data model and a Bayesian estimation and inference procedure. We demonstrated its superiority over existing methods via exhaustive simulation studies and its robustness via sensitivity analyses concerning both bandwidth and prior selection. DMCFB is flexible in terms of adding any source of variation and is robust with respect to the true underlying methylation pattern. Missing values are automatically imputed. The method is capable of adding discrete or continuous covariates or combinations. Most existing methods ignore read depth information in the analysis; by adding the read depth as an additional source of variation, our proposed method adjusts the methylation levels for better estimation. Similar to DMCHMM, our proposed method shows consistent behavior; the results depend on the difference in methylation between groups and not other aspects of dmrs such as length, location, autocorrelation pattern, read depth, etc. One drawback of DMCHMM is that the efficiency deteriorates if the distances between cpgs are explicitly included in hmm. Our proposed method is not restricted in this sense. In DMCFB, we reduced the need for the imputation of read depths and methylation counts by setting them to zero and letting the functional pattern estimates the missing methylation levels. This approach has reduced the computation time and increased the performance.

Among others, Ref. [52] has shown that using centered parameterization is a better approach for mcmc efficiency and speed when there are parameters unidentifiable from observed data, such as missing values. They proposed the ancillarity–sufficiency interweaving strategy (asis). As a future work, we intend to examine the utility of, and implement, asis in DMCFB whenever missingness is a significant factor.

There are multiple approaches for controlling family-wise errors. One may use SimBaS and global Bayesian *p*-values [45] or Bayesian fdrs [46]. Our simulation study showed that our proposed method works better than SimBaS (Supplementary S6). We intend to add SimBaS as an alternative approach in DMCFB. Note that Ref. [53] observed inflation in fdr in their simulations. In our settings, we observed better fdrs for SimBas but better accuracy, sensitivity, and CKappa with DMCFB.

While reanalyzing the apl data, we noted that BiSeq’s default significance threshold maintains a very stringent control over the false discovery rate, and only a small number of dmcs are identified; in fact, DMCFB identifies overall 6 (Chr 15) to 10 (Chr 17) times more dmcs than BiSeq. However, it is possible to adjust the BiSeq package options slightly, and we ran another series of analyses where the BiSeq’s call rate was 7.5% (Supplementary Table S11 (A)) instead of 6.5% (Table 1 (A)) on Chr 15. However, the results of this sensitivity analysis showed less agreement with DMCFB, i.e., among dmcs called by BiSeq, the percentage also called by DMCFB dropped from 76.12% (Table 1 (A)) to 62.87% (Supplementary Table S11 (A)) on Chr 15. In contrast, among cpgs not called by BiSeq, the percentage of dmcs called by our method remained essentially unchanged from Table 1. This suggests that when BiSeq is less confident in a call, the two methods are using nearby reads and methylation patterns differently. Furthermore, the ability to capture more dmcs in cpg islands reflects DMCFB’s ability to use information from adjacent cpgs via our functional smoothing. Given the density of cpgs in the islands, we obtain much more sensitivity in these regions. DMCFB, due to the functional regression and efficient imputation, seems to better capture a signal that persists across several cpgs. DMCFB often identifies all cpgs in an island as differentially methylated, whereas BiSeq tends to capture less than half. Similarly, we have shown that if an island displays differential methylation, that DMCFB more often also finds differential methylation in the adjacent shores. Similarly, the additional sensitivity of DMCFB is also visible when examining the known binding sites of *PML-RAR*α; the performance of our approach was particularly notable when the read depths were moderate. The two methods are both smooth, but they use the information differently.

Let us elaborate on several computing challenges and the techniques implemented in DMCFB. (1) *Partitioning*: We partition data for two reasons: one, to avoid using a big dimensional design matrix which results in very high-dimensional computation and memory problems; two, to utilize multi-cores to increase the speed. We recommended partitioning the data into regions of size 500 and using a common resolution of 30. (2) *Parallel computing*: Implementing parallel computing results in faster computation. One can (i) run the computation on several partitions simultaneously or (ii) use further parallel computing while estimating $\beta_{t,g,i}$. (3) *Multi-resolution*: To speed up the process, one can use a multi-resolution model choosing different bandwidths for the parameters $\left(\gamma_{0},\delta_{g},\upsilon_{g,i}\right)$. For example, one may choose a larger bandwidth for $\upsilon_{g,i}$ and a smaller one for $\left(\gamma_{0},\delta_{g}\right)$.

In summary, our proposed method provides an efficient dmc/dmr identification method.

## Figures and Tables

**Table 1:** Comparing DMCFB and BiSeq in identifying dmcs in the apl data.

*(A) Chromosome 15*							
		BiSeq					
	DMCFB	ndmc	% of 2 × 2 table	%ndmc(BiSeq)	dmc	% of 2 × 2 table	%dmc(BiSeq)
All cpgs	ndmc	193,554	67.81		4421	1.55	
	dmc	73,370	25.70	27.49	14,092	4.94	76.12
cpg Island	ndmc	33,956	45.28		3280	4.37	
	dmc	27,235	36.32	44.51	10,516	14.02	76.22
cpg Shore	ndmc	16,236	57.70		614	2.18	
	dmc	9133	32.45	36.00	2158	7.67	77.85
cpg Desert	ndmc	143,362	78.64		527	0.29	
	dmc	37,002	20.30	25.60	1418	0.78	72.90
*(B) Chromosome 17*							
		BiSeq					
	DMCFB	ndmc	% of 2 × 2 table	%ndmc(BiSeq)	dmc	% of 2 × 2 table	%dmc(BiSeq)
All cpgs	ndmc	343,989	67.40		3576	0.70	
	dmc	147,687	28.94	30.04	15,134	2.97	80.89
cpg Island	ndmc	74,238	53.46		2887	2.08	
	dmc	50,483	36.35	40.48	11,260	8.11	79.59
cpg Shore	ndmc	53,027	61.89		531	0.62	
	dmc	29,091	33.96	35.43	3026	3.53	85.07
cpg Desert	ndmc	216,724	75.82		158	0.06	
	dmc	68,113	23.83	23.91	848	0.30	84.29

**Table 2:** Reanalysis of the apl data using DMCFB by accounting for all available information.

			Chromosome 15				Chromosome 17			
α	**Pairwise Contrasts**		ndmc **%**	dmc **%**	**Hyper%**	**Hypo%**	ndmc **%**	dmc **%**	**Hyper%**	**Hypo%**
0.05	pmc	cd34	98.338	1.662	56.08	43.92	98.93	1.07	65.05	34.95
	rbm	cd34	99.614	0.386	59.22	40.78	99.54	0.46	51.09	48.91
	apl	cd34	98.025	1.975	80.60	19.40	97.69	2.31	72.19	27.81
	rbm	pmc	99.872	0.128	70.41	29.59	99.78	0.22	22.81	77.19
	apl	pmc	99.587	0.413	47.84	52.16	99.30	0.70	51.03	48.97
	apl	rbm	99.452	0.548	60.29	39.71	99.06	0.94	47.74	52.26
	All positions		95.557	4.443	–	–	95.18	4.82	–	–
$10^{-5}$	pmc	cd34	99.999	0.001	0.00	100.0	100.0	0.00	0.00	0.00
	rbm	cd34	99.998	0.002	100.0	0.00	99.999	0.001	0.000	100.0
	apl	cd34	99.934	0.066	86.70	13.30	99.96	0.04	74.41	25.59
	rbm	pmc	100.00	0.000	0.00	0.00	100.0	0.00	0.00	0.00
	apl	pmc	99.999	0.001	100.0	0.00	99.99	0.01	13.21	86.79
	apl	rbm	99.993	0.007	38.10	61.90	99.99	0.01	0.00	100.0
	All positions		99.926	0.074	–	–	99.94	0.06	–	–
$10^{-8}$	pmc	cd34	99.999	0.001	0.00	100.0	100.0	0.00	0.00	0.00
	rbm	cd34	99.998	0.002	100.0	0.00	99.9998	0.0002	0.00	100.0
	apl	cd34	99.935	0.065	88.04	11.96	99.96	0.04	73.91	26.09
	rbm	pmc	100.00	0.000	0.00	0.00	100.0	0.00	0.00	0.00
	apl	pmc	99.999	0.001	100.0	0.00	99.99	0.01	13.21	86.79
	apl	rbm	99.993	0.007	38.10	61.90	99.99	0.01	0.00	100.0
	All positions		99.927	0.073	–	–	99.95	0.05	–	–

## Data Availability

Web-based supplements including a list of notations and acronyms, tables, figures, and extra data analyses referenced here are available with this paper at the *Biomolecules* website. Our open-source package DMCFB [59] is available at https://bioconductor.org/packages/DMCFB/, accessed on 24 May 2024. An early version of this paper was published in bioRxiv [60].

## Supplementary Materials

The following supporting information can be downloaded at: https://www.mdpi.com/article/10.3390/biom14060639/s1. References [54,55,56,57,58] are cited in the Supplementary Materials.
