# Supplementary material for: Identifying Differential Methylation in Cancer Epigenetics via a Bayesian Functional Regression Model
Source: Biomolecules. 2024 May 29;14(6):639. doi: 10.3390/biom14060639 (PMC11201607; doi:10.3390/biom14060639)
Supplement: Supplementary file 1 [file biomolecules-14-00639-s001.zip › biomolecules-2968843-supplementary.pdf]

Supplementary Materials: Identifying Differential Methylation in Cancer Epigenetics via a Bayesian Functional Regression Model

Farhad Shokoohi [1,*], David A. Stephens [2] and Celia M. T. Greenwood [3,4,5]

Outline:

We present Supplementary Materials to "Identifying Differential Methylation in Cancer Epigenetics via a Bayesian Functional Regression Model" which contains the following sections:

- Section S1 gives brief information about the two methylation data sets discussed in the main paper.
  - We plot the methylation patterns in both data sets and we show that the patterns are too complex for almost all methods to handle.
- Section S2 displays a figure related to the BLK data set.
  - We show that, in general, methylation level has a systematic relationship with read-depth.
- Section S3 compares the selected methods in the main paper.
  - We presented a table in which a few of the capabilities of existing methods are compared.
- Section S4 presents the results of the simulation study discussed in the main paper.
  - We show the simulation setups for different scenarios and plot the actual DMRs along with simulated data and real data for one sample (Section S4.1).
  - We present the rest of the results for simulation study Scenario 1 with $\sigma = 0.18$ (Section S4.2). We show that the results indicate that our proposed method, i.e. `DMCFB`, is superior to the existing methods by using different criteria.
  - We present all the results related to Scenario 1 with $\sigma = 0.24$ (Section S4.3) which indicate results similar to the previous scenario. Although there is a drop in the performance in all methods, the decline in our method is negligible.
  - We present all the results for simulation study Scenario 2 (Section S4.4). We show that similar to previous results, `DMCFB` outperforms all the existing methods.
- Section S5 gives the sensitivity analysis for `DMCFB`.
  - We show that the results are generally robust to the choice of bandwidth (Section S5.1 and S5.2), although precision decreases slightly as the bandwidth increases.
  - We show that the results are generally robust to the choice of prior (Section S5.3), although an empirical approach gives better performance.
  - We show that the results are generally robust to the simultaneous choice of band-with and partitioning (Section S5.4).
- Section S6 give a comparison between `DMCFB` and `SimBas` methods in detecting DMCs
- Section S7 gives the rest of the details of the BLK data analysis discussed in the main paper.
  - We show that there is widespread hypomethylation in B-cells compared with T-cells and monocytes.
- Section S8 provides details of the APL data analysis and comparisons between `DMCFB`, `BiSeq`, and `DMCHMM`.
  - In Section S8.1, we give additional results from the reanalysis of the APL data in the main paper.

- In Section S8.2, another comparison between `DMCFB` and `BiSeq` using the APL data is given which confirms the results in the main paper.
- In Section S8.3, a comparison between `DMCFB` and `DMCHMM` using the APL data is given which shows less agreement between the two methods in some criteria and more agreement in some other criteria compared with those of `BiSeq`.

Notation and Acronyms

The following notation and acronyms are used in the main paper and the supplementary materials.

- Plat. : Platform based on which methylation data are generated;
- r.NA : Missing values are removed;
- filter : Data are filtered to remove positions with low read-depth;
- Clustering : Clustering sites prior to analysis;
- Smoothing : Smoothing methylation levels;
- Parallel : Parallel Computing;
- Stats. : Providing Statistics such as p-values, FDRs, etc;
- BSS : BS-Seq technology for retrieving methylation status;
- BiSeq : The `BiSeq` method
- bsseq : The `bsseq` method
- bump : The `bumphunter` method
- DMCFB : The `DMCFB` method
- DMCHMM : The `DMCHMM` method without weights
- DMCHMMw : The `DMCHMM` method with weights
- DMRca : The `DMRcaller` method
- DSS : The `DSS` method
- HFish : The `HMM-Fisher` method
- HMMDM : The `HMM-DM` method
- bed : A file type for storing methylation data
- NDMR, NonDMR : Not a DMR
- NDMC : Not a DMC
- STR, END : Start and end position of a DMR
- *RARA*: Retinoic Acid Receptor Alpha gene.
- *RARα*: retinoic acid receptor alpha protein.
- *PML-RARα* : Promyelocytic leukemia/retinoic acid receptor alpha.
- BC-Seq : Bisulphite Conversion followed by Capture and Sequencing
- BSPP : Bisulphite Padlock Probe
- RRBS: Reduced-Representation Bisulfite Sequencing
- 5hmC : 5-hydroxymethylcytosines

S1. Two methylation data sets

Two publicly available data sets are used in the main paper. The BLK data set is used to develop our method (DMCFB) and to perform simulation studies. For further assessment of the proposed method, we use APL data. The data sets are described in detail in the following.

S1.1. BLK *data*

A WGBS data set from whole blood was collected on a cohort of healthy individuals from Sweden. The data set is obtained from the Genome Québec Innovation Centre (Cheung et al., 2017). There are 19 T-cell samples, 13 monocyte samples and 8 B-cell samples in the data. The Illumina HiSeq2000/2500 system was used to sequence the data. Shokoohi et al. (2019) used these data as motivation and to run simulation studies for comparison of DNA methylation analytical pipelines. We follow Shokoohi et al. (2019) and use part of the data as a motivation. We examine the data near the *BLK* gene on human chromosome 8. The region contains 30,440 CpG sites spanning 2 MB (10,352,236 - 12,422,082) in which approximately 23.39% of the positions have missing information in at least one of the samples and 21% have at least 20 missing values across all samples. An illustration of the data is given in Figure S1. For each cell type, smooth curves were obtained by: first, aggregating the data (summing read-depths and summing methylation counts over cell type samples at each position), second, imputing the missing values (using a naive method), and third, fitting a smooth curve.

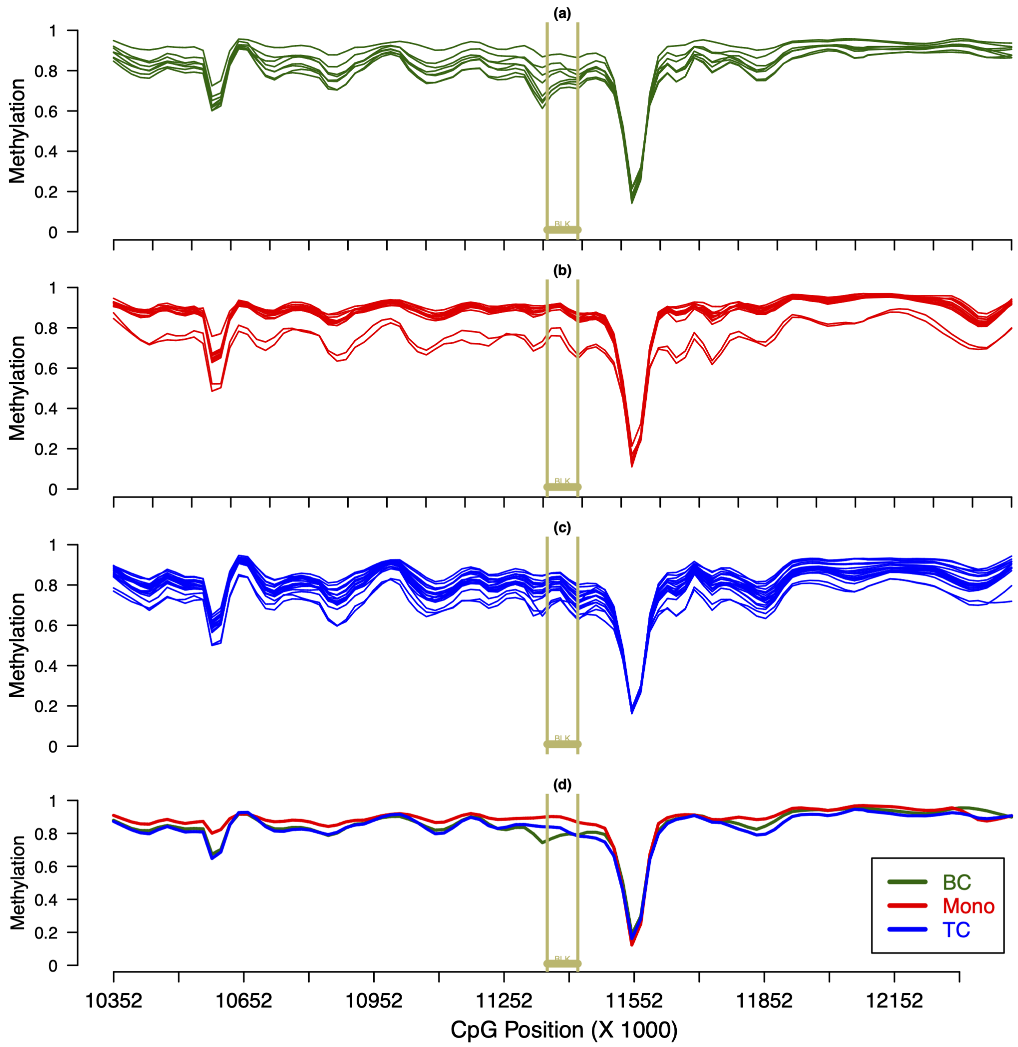

Figure S1. Lowess smoothed profiles of methylation levels near the *BLK* gene for 3 cell types are shown for (a) 8 B-cell samples, (b) 13 monocyte samples and (c) 19 T-cell samples. (d) Smooth aggregated profiles for each cell type are overlaid. The smooth curves were obtained by first aggregating (summing) the read-depths and aggregating methylation counts for each cell type at each position, imputing the missing values, obtaining the methylation levels and finally smoothing using lowess (span = 0.05).

S1.2. APL *data*

An RRBS data set is collected from patients with Acute Promyelocytic Leukemia (APL). The data set is publicly available on the Gene Expression Omnibus (accession no. GSE42119) (Schoofs et al., 2013). These data contain 18 APL patients, and 16 control samples. The control samples include 8 bone marrow samples from patients in remission, four healthy CD34$^+$ cells and four promyelocytes. These data have been analyzed in several articles including Hebestreit et al. (2013). An illustration of the data on chromosome 17 is given in Figure S2. We have plotted smooth curves of each sample in both APL and control groups. We also plotted the smooth average of methylation levels for groups using lowess (span = 0.00001).

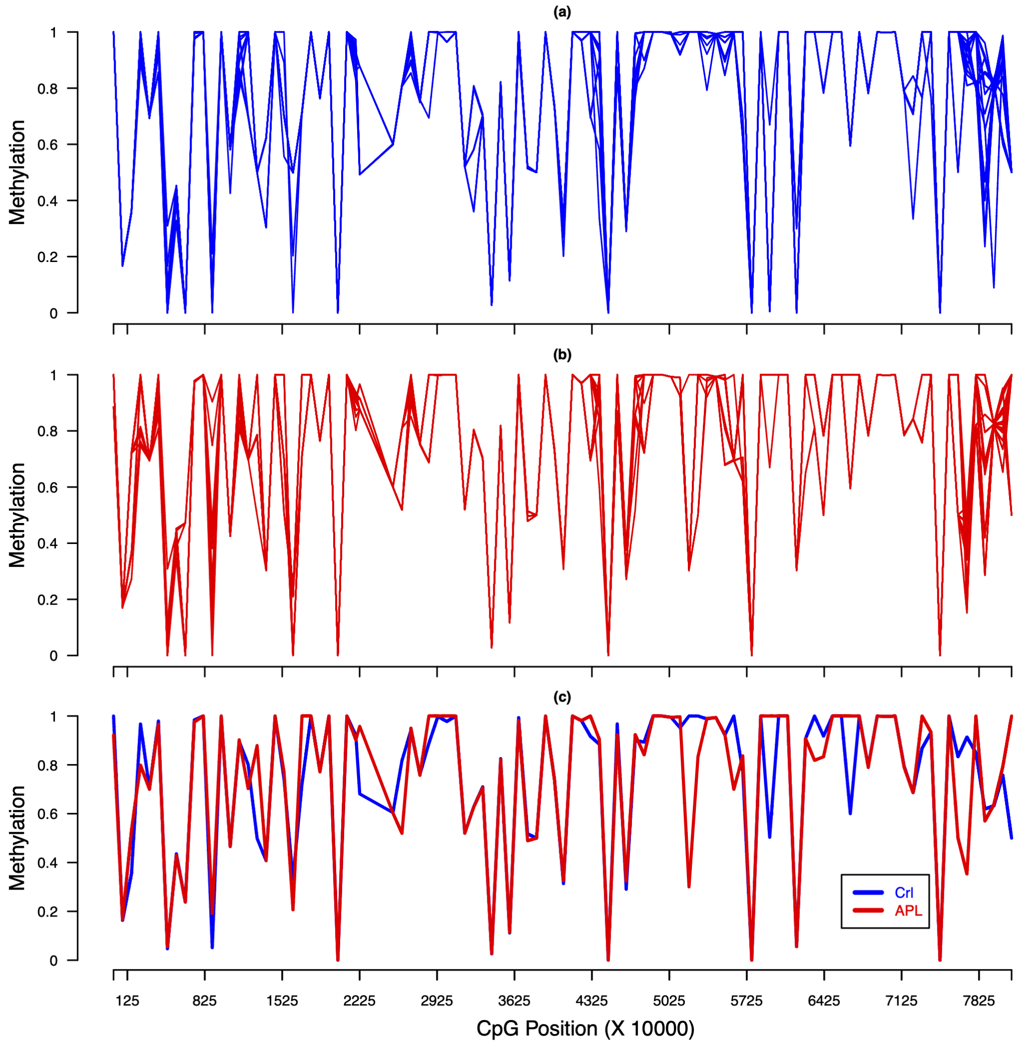

Figure S2. Lowess smoothed profiles of methylation levels for chromosome 17 for two groups are shown for (a) 16 control samples, and (b) 18 APL patients. (c) Smooth aggregated profiles for each group are overlaid. The smooth curves were obtained by first aggregating (summing) the read-depths and aggregating methylation counts for each group at each position, imputing the missing values, obtaining the methylation levels, and finally smoothing using lowess (span = 0.00001).

S2. Plots

In this section, we provide some preliminary plots to examine the data.

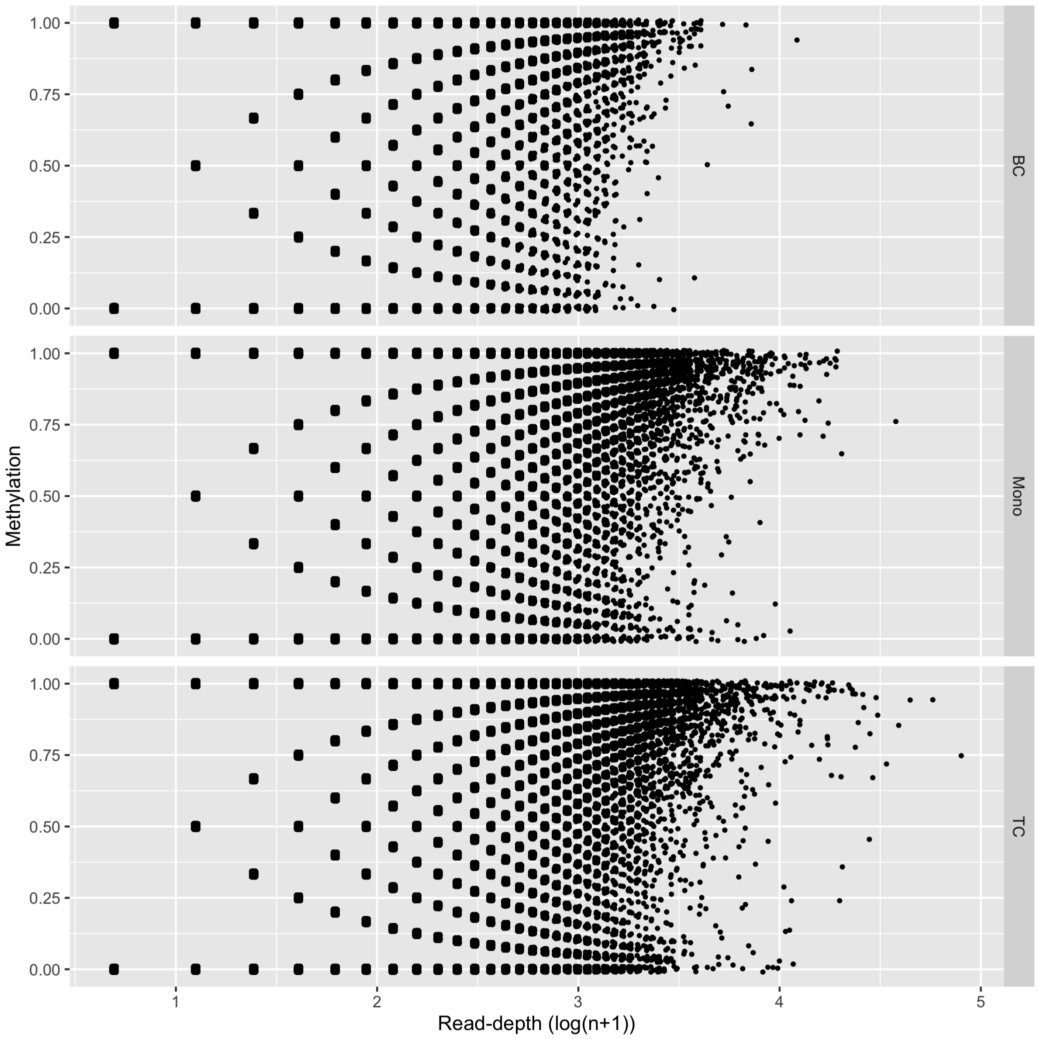

Figure S3. Methylation level versus log(read-depth + 1) in BLK data separated for each cell type; A small amount of jitter is added to the points.

S3. List of Selected Methods for Comparison

The chosen methods are presented in Table S1. The overall performance of DSS-Single, for instance in terms of accuracy, was close to that of DSS, although different for individual DMRs and non-DMRs, thus its results are omitted. For bumphunter, all positions, even those with missing values, were included in the analysis to improve the performance. In bsseq, we have removed positions where all the samples in one group were missing at that position. In HMM-Fisher, distances between CpGs were set to 1 to avoid crashing. See Table S1 for the remaining modifications.

We were not able to include some methods in simulation studies such as MethPipe (Song et al., 2013), methylPipe (Kishore et al., 2015), methylKit (Akalin et al., 2012), and ComMet (Saito and Mituyama, 2015) since these tools require specific file formats, or we were unable to implement them. In any case, these methods have been reported to be inferior to some of the methods that were used for comparison in this study (Klein and Hebestreit, 2015; Wu et al., 2015; Yu and Sun, 2016).

Table S1. List of DNA methylation analytical tools with their features and capabilities, and the changes made to increase their efficiency

Package	Model	Platform	Data	r.NA	filter	Clustering	Smoothing	Parallel	Notes
BiSeq	beta regression	BSS	bed	✓	✓	✓	✓	partly	The default "perc.samples" is changed from 4/5 to 1/2.
bsseq	binomial & linear	BSS	bed	✓		✓	✓	✓	"abs(meanDiff)" is changed to 0.1.
bumphunter	linear mixed model	BSS	reads	✓	✓	✓	✓	✓	"cutoff" is changed to 0.05. No position was removed.
DMCHMM	HMM	BSS	reads bed			✓	✓	✓	—
DMRcaller	Kernel smoothing	BSS	custom	✓	✓	✓	✓	✓	"minProportionDifference" is changed from 0.4 to 0.1.
DSS	Bayesian hierarchical	BSS RNA	reads	✓	✓				"p.threshold" is set to 0.05.
DSS-Single	Bayesian hierarchical	BSS RNA	reads	✓	✓		✓		"p.threshold" is set to 0.05.
HMM-DM	HMM	BSS	reads	✓	✓		✓		"meanDiff.cut", "max.distance" and "max.EM" are changed from 0.3, 100 and 1 to 0.1, 1000 and 300, respectively.
HHM-Fisher	HMM	BSS	reads				✓		Distances between CpGs are set to 1 to avoid crashing.
WFMM	Wavelet-based functional mixed model		custon	✓	✓		✓	✓	Only Windows; no R package; difficult to implement parallel computing.

S4. Simulation Study from the Main Paper

S4.1. Simulation Set-ups

In the following table we present the simulation set-ups for the first and second scenarios in Tables S2 and S3, respectively.

Table S2. Simulation setup parameters for the first scenario

DMR	Chromosome	Start position	End position	Effective size	Length
DMR1	8	10431839	10511940	0.1	1620
DMR2	8	10671903	10751815	0.2	1094
DMR3	8	10911977	10991817	0.3	1205
DMR4	8	11231950	11311987	0.4	1267
DMR5	8	11631990	11641956	0.4	126
DMR6	8	11671950	11681894	0.3	102
DMR7	8	11714976	11725000	0.2	223
DMR8	8	11783926	11793886	0.1	224
DMR9	8	11870570	11924576	0.05	924
DMR10	8	12084164	12191969	0.02	433

Table S3. Simulation setup parameters for the second scenario

DMR	Chromosome	Start position	End position	Effective size	Length
DMR1	8	10431839	10441997	0.1	172
DMR2	8	10701967	10729553	0.2	363
DMR3	8	10951995	10971974	0.3	274
DMR4	8	11281992	11301965	0.4	358
DMR5	8	11584972	11629892	0.4	903
DMR6	8	11679894	11714976	0.3	643
DMR7	8	11764964	11794972	0.2	703
DMR8	8	11874947	11924642	0.1	669
DMR9	8	12044993	12061576	0.05	307
DMR10	8	12204790	12232000	0.02	120

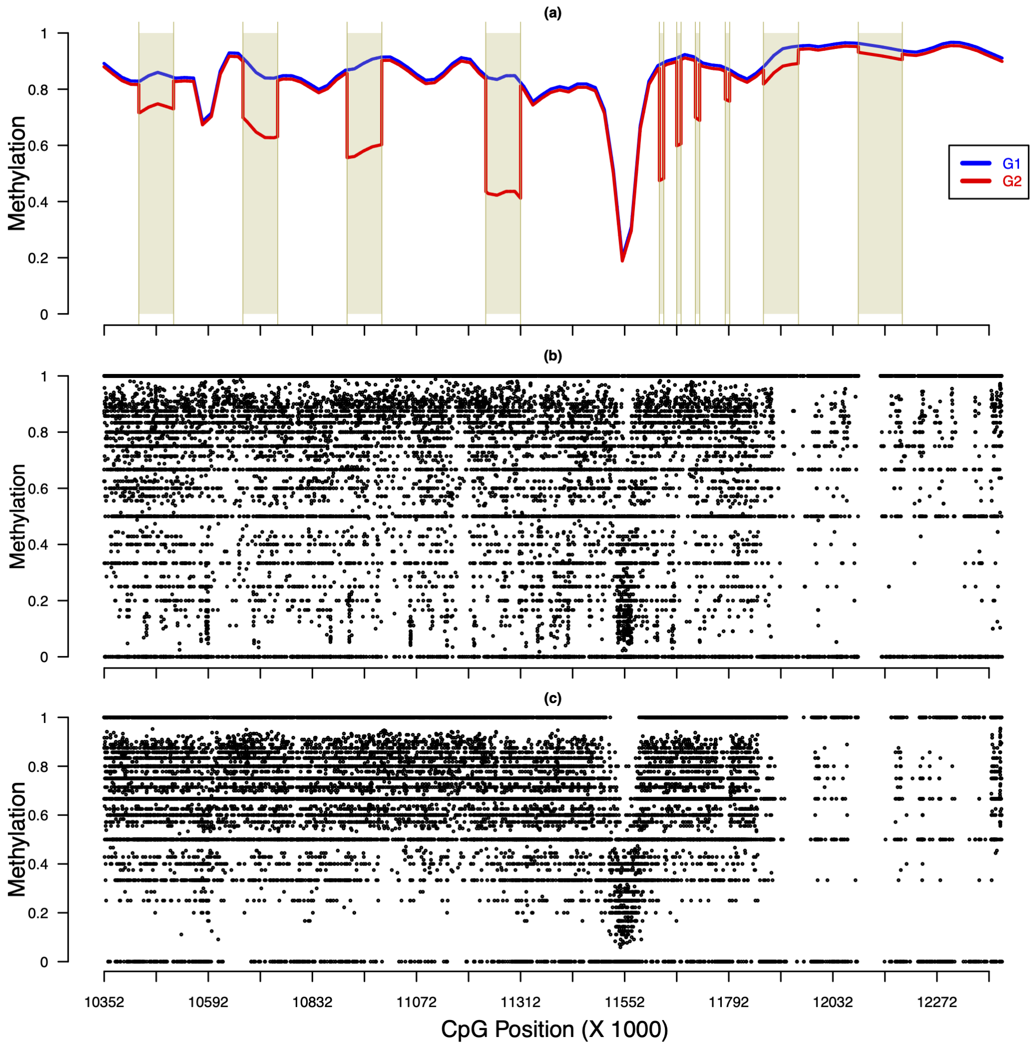

Figure S4. Methylation profiles illustrating simulation design for the first scenario. (a) The smooth methylation profile (G1), obtained by smoothing aggregated B-cell samples, from which the true mean methylation levels in the simulated data sets are generated. (b) Actual methylation proportions for one B-cell sample. (c) A set of simulated methylation data for the same sample as in (b), with error generated from a normal distribution with standard deviation equal 0.18.

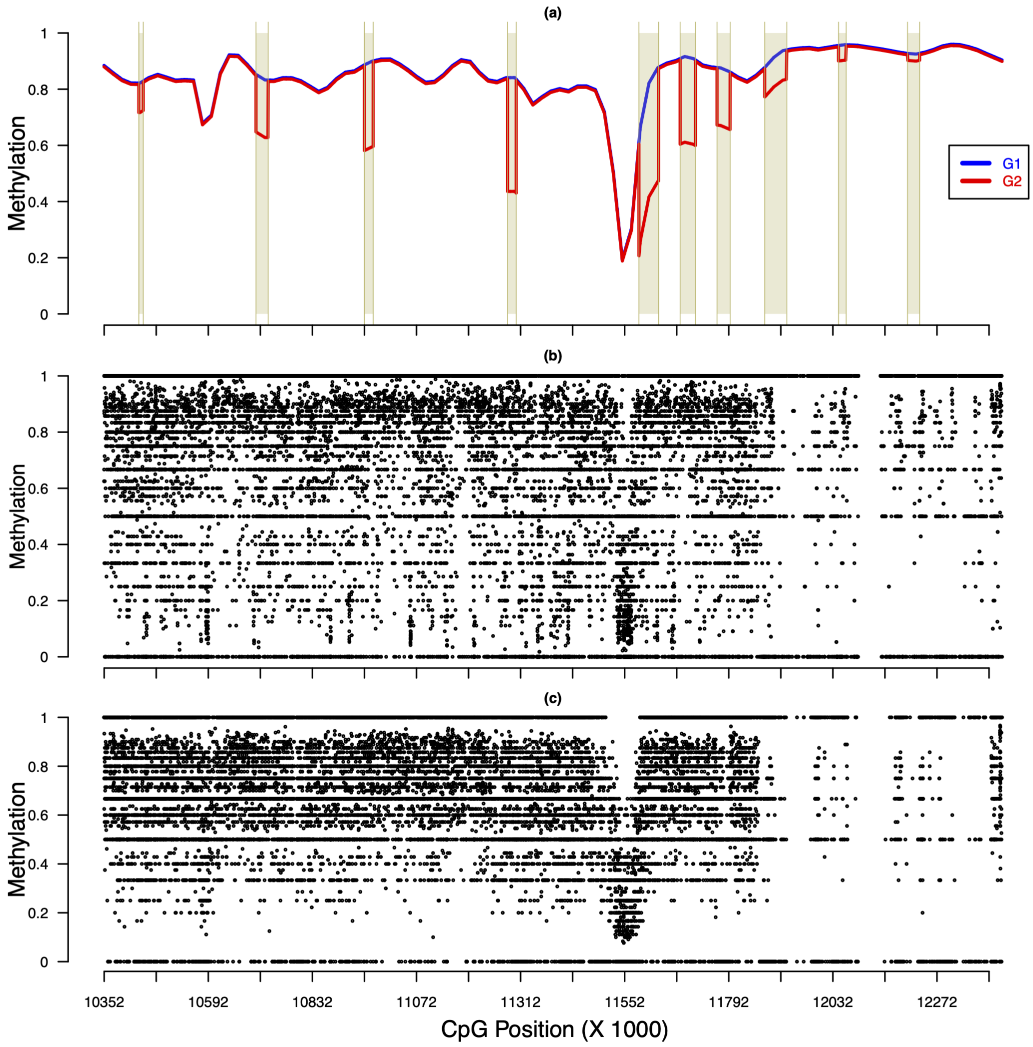

Figure S5. Methylation profiles illustrating simulation design for the second scenario. (a) The smooth methylation profile (G1), obtained by smoothing aggregated B-cell samples, from which the true mean methylation levels in the simulated data sets are generated. (b) Actual methylation proportions for one B-cell sample. (c) A set of simulated methylation data for the same sample as in (b), with error generated from a normal distribution with standard deviation equal 0.18.

S4.2. Figures and Tables of Simulation Setup Scenario 1, $\sigma = 0.18$

In this section we provide the results of simulation study for the first scenario when errors are generated from a normal distribution with standard deviation equal to 0.18.

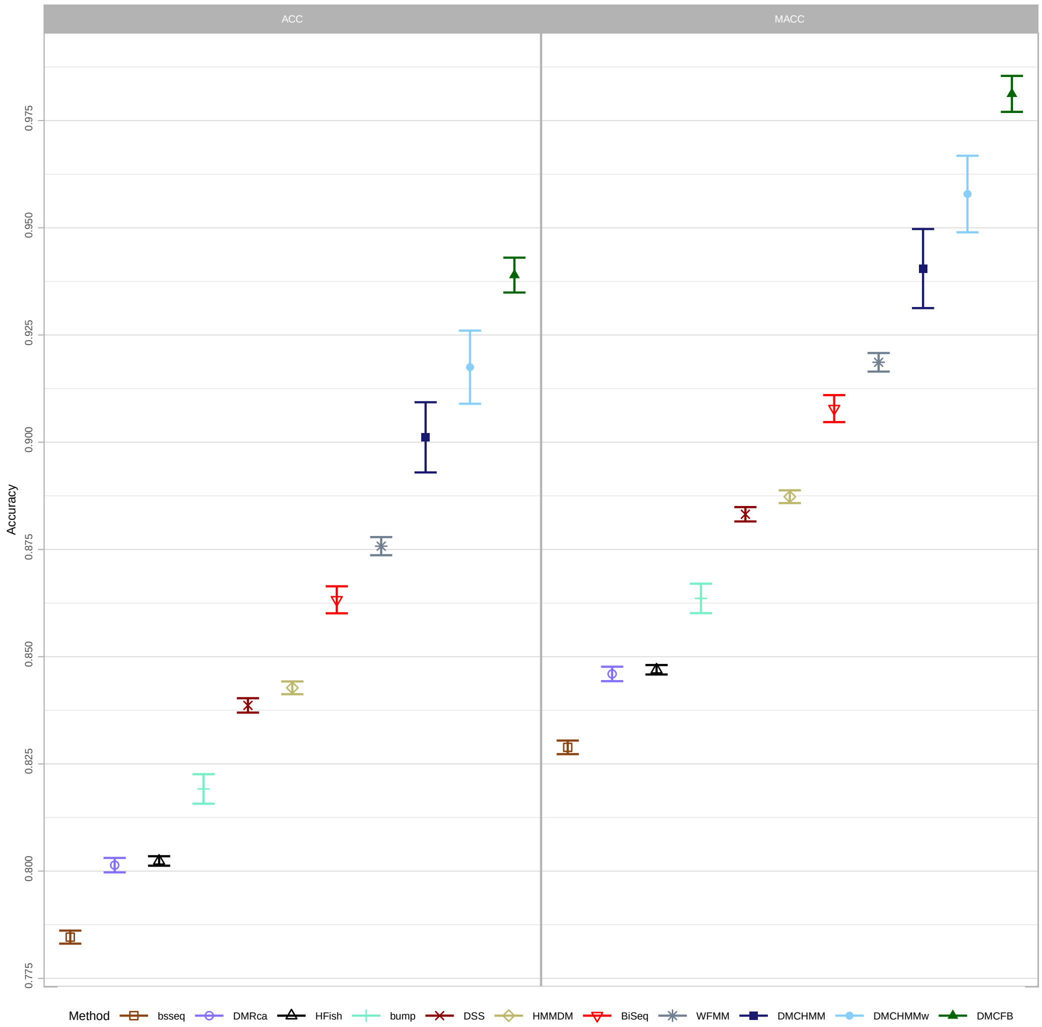

Figure S6. The average overall accuracy (ACC) and average overall modified accuracy (MACC) for different methods in simulated data for the first scenario; Errors are generated from $N(\mu = 0, \sigma = 0.18)$. Relevant results are reproduced from Shokoohi et al. (2019). (sd error bars are added.)

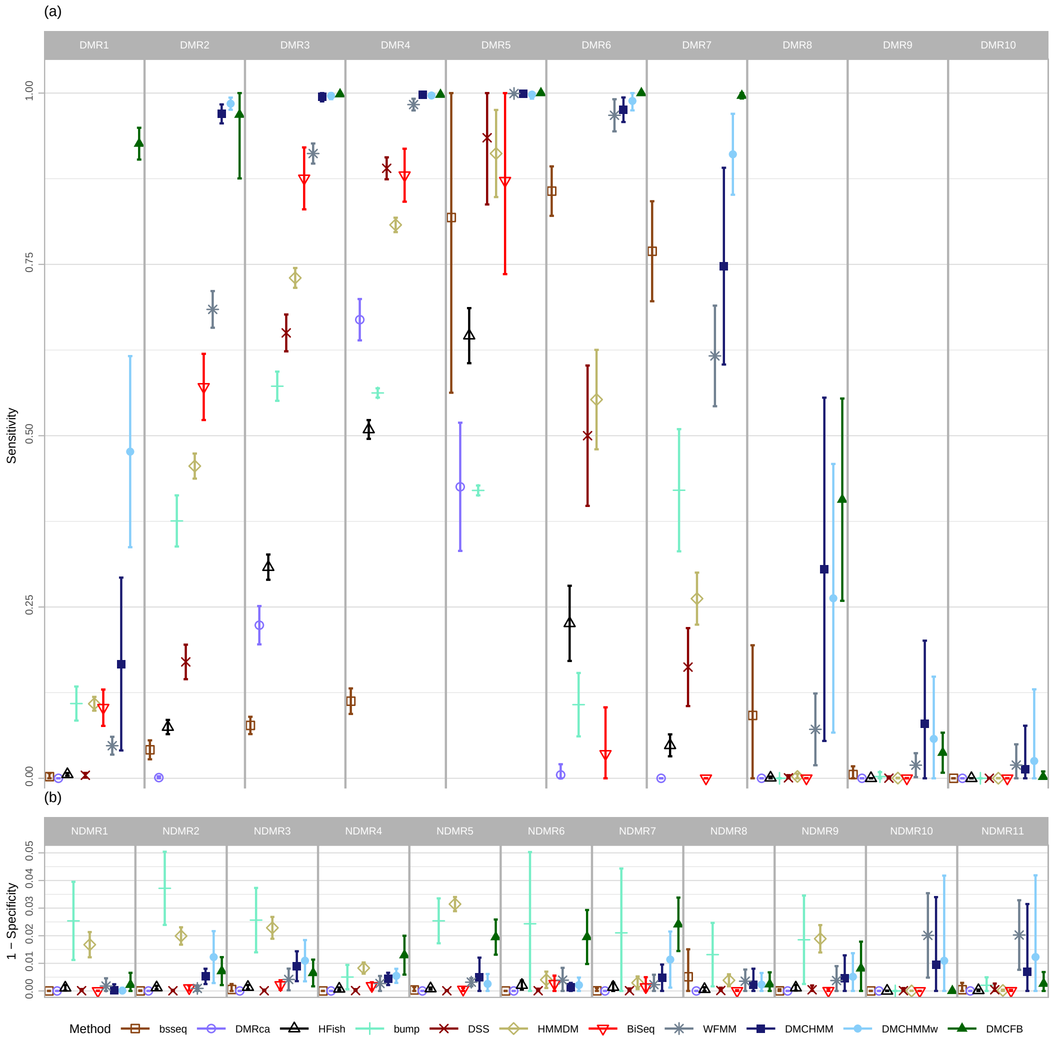

Figure S7. (a) The average proportion of correctly identified DMCs (SE_j) for each method separated by DMRs; (b) The average proportion of incorrectly identified DMCs ('1-Specificity') for each method separated by NDMRs (The axis is truncated) in simulated data for the first scenario; Errors are generated from $N(\mu = 0, \sigma = 0.18)$. Relevant results are reproduced from Shokoohi et al. (2019). (sd error bars are added.)

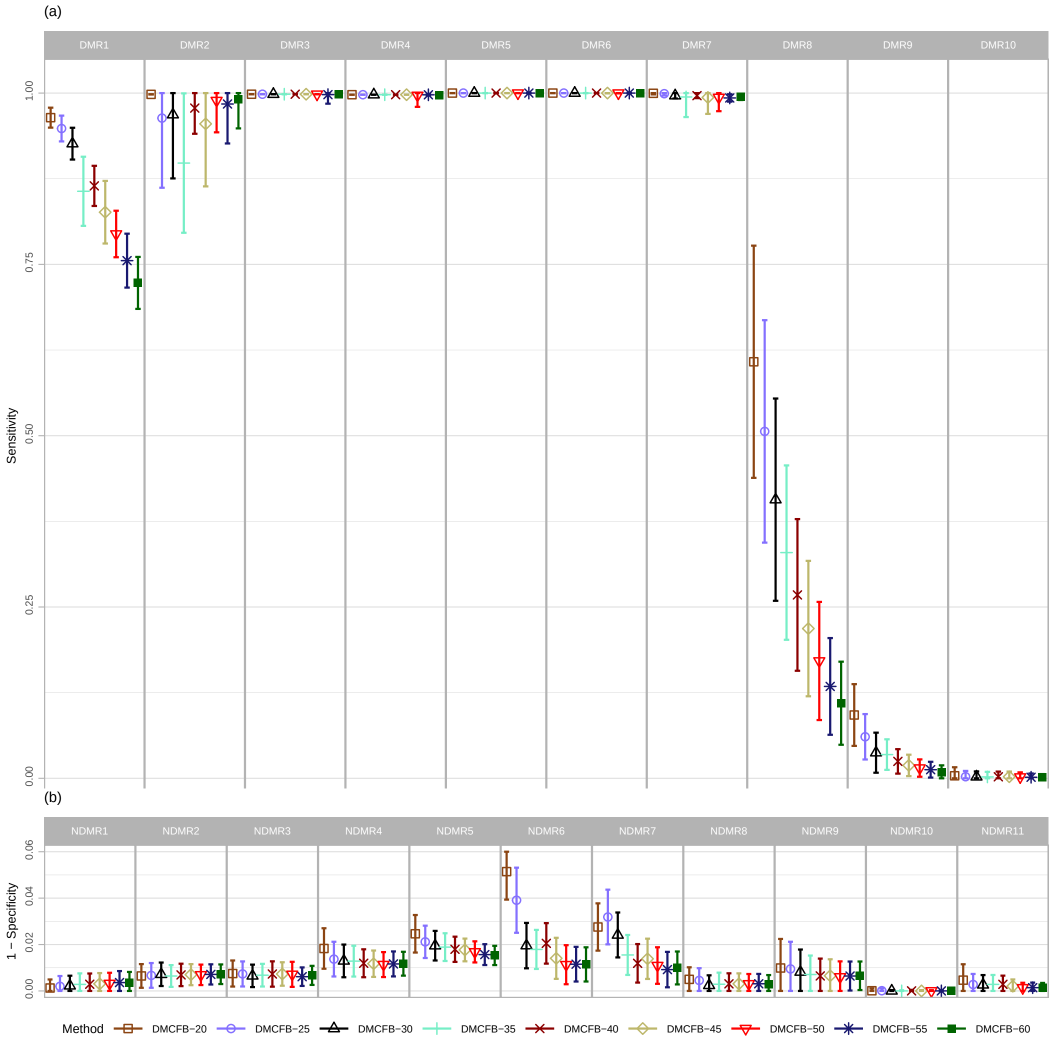

Figure S8. (a) The average proportion of correctly identified DMCs ('Sensitivity') for each choice of band-width separated by DMRs DMCFB; (b) The average proportion of incorrectly identified DMCs ('1-Specificity') for each choice of band-width separated by NDMRs in DMCFB (The axis is truncated) in simulated data for the first scenario; Errors are generated from $N(\mu = 0, \sigma = 0.18)$. (sd error bars are added.)

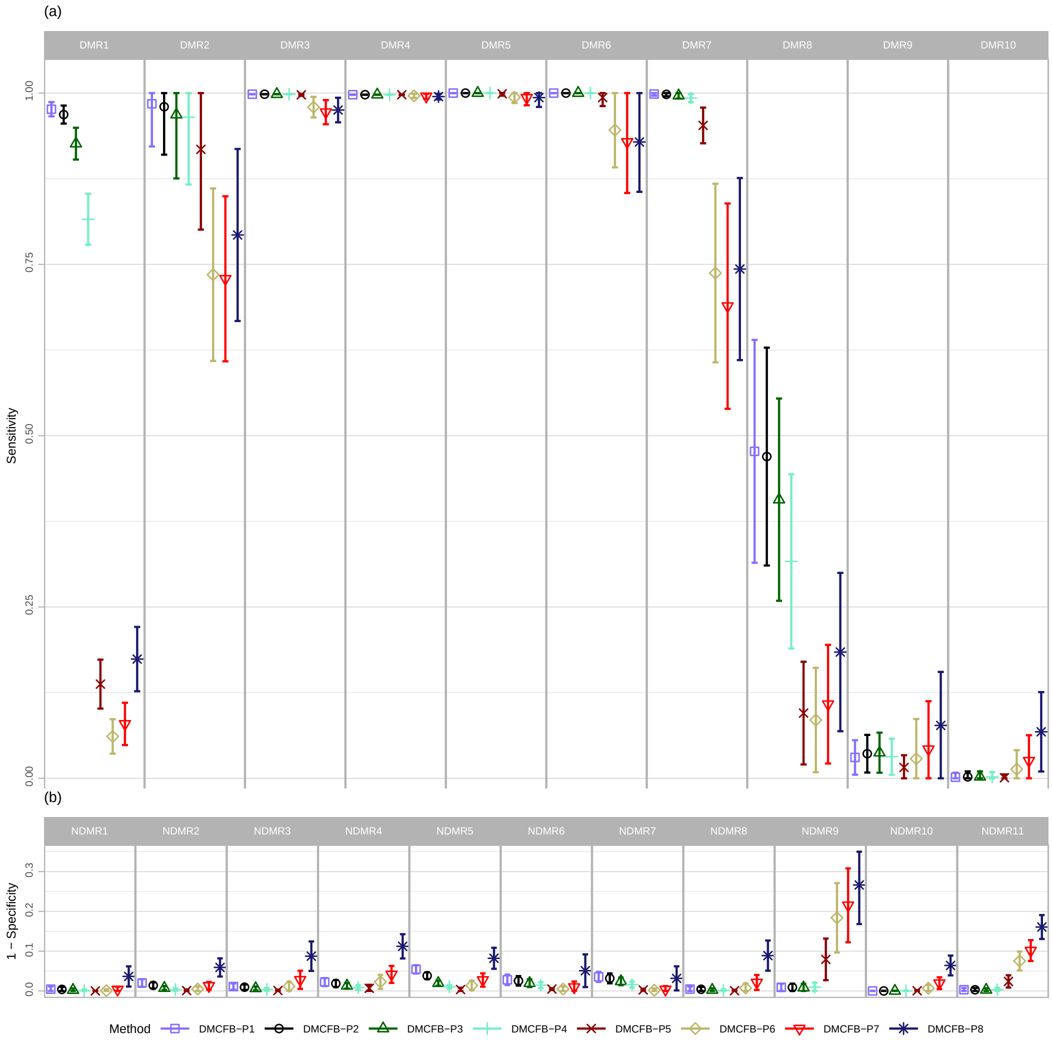

Figure S9. (a) The average proportion of correctly identified DMCs ('Sensitivity') for each choice of precision of prior separated by DMRs in DMCFB; (b) The average proportion of incorrectly identified as DMCs ('1-Specificity') for each choices of precision of prior separated by NDMRs in DMCFB (The axis is truncated) in simulated data for the first scenario. Errors are generated from $N(\mu = 0, \sigma = 0.18)$. The precision is chosen, in order, in $\{30, 20, 10, 1/0.18, 1, 0.3, 0.2, 0.1\}$. (sd error bars are added.)

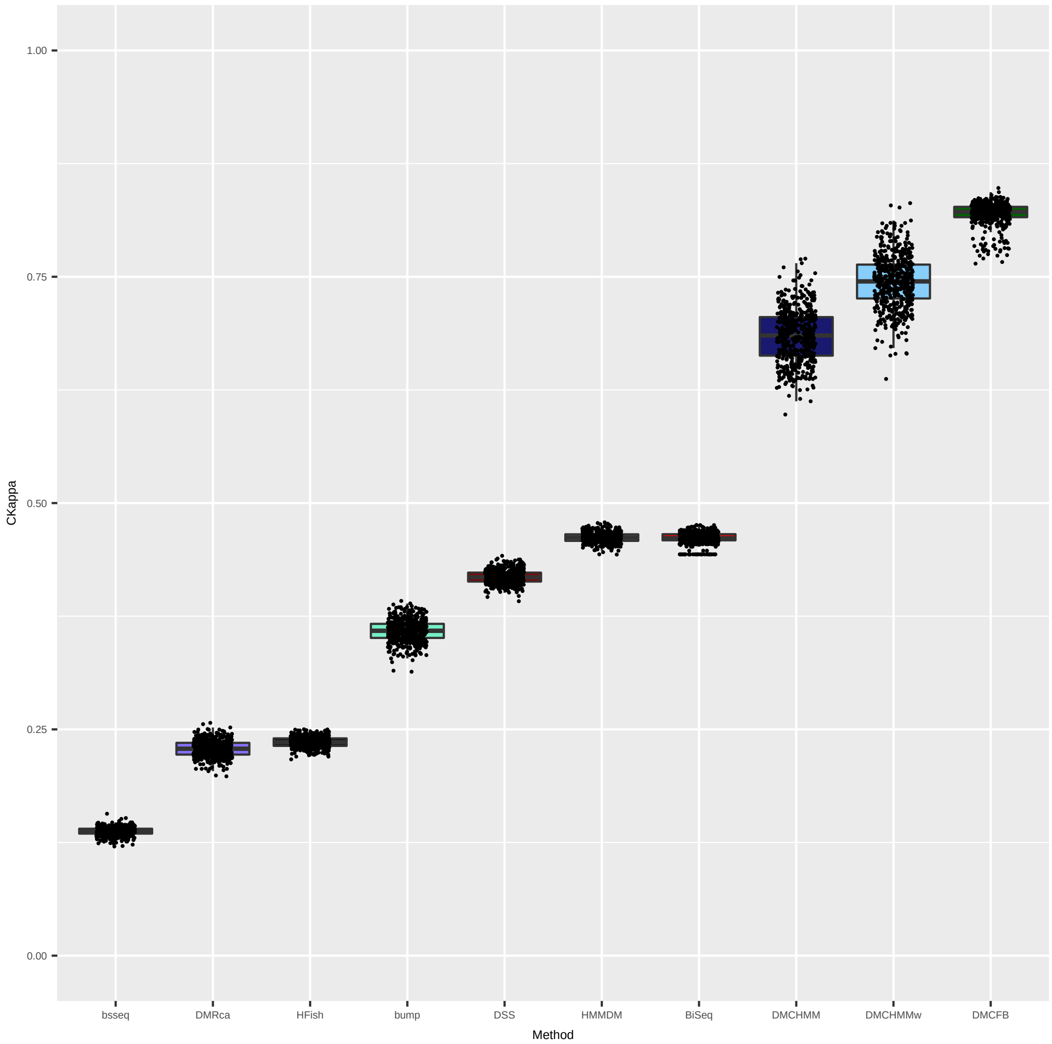

Figure S10. The box-plots of Cohen's Kappa for each method in simulated data for the first scenario; Errors are generated from $N(\mu = 0, \sigma = 0.18)$.

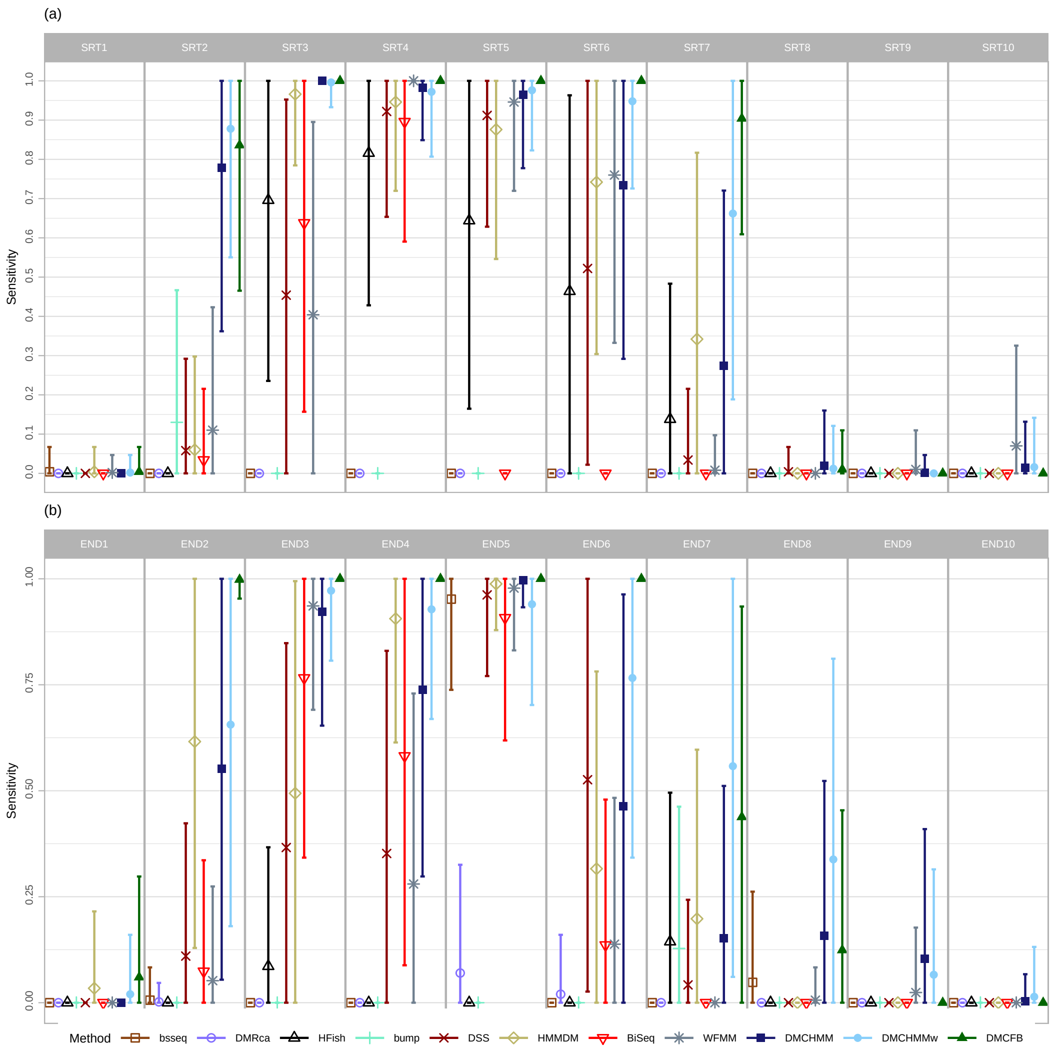

Figure S11. Average number of times that the start (a) and end (b) positions of DMRs are identified as DMCs for different methods in simulated data for the first scenario; Errors are generated from $N(\mu = 0, \sigma = 0.18)$.

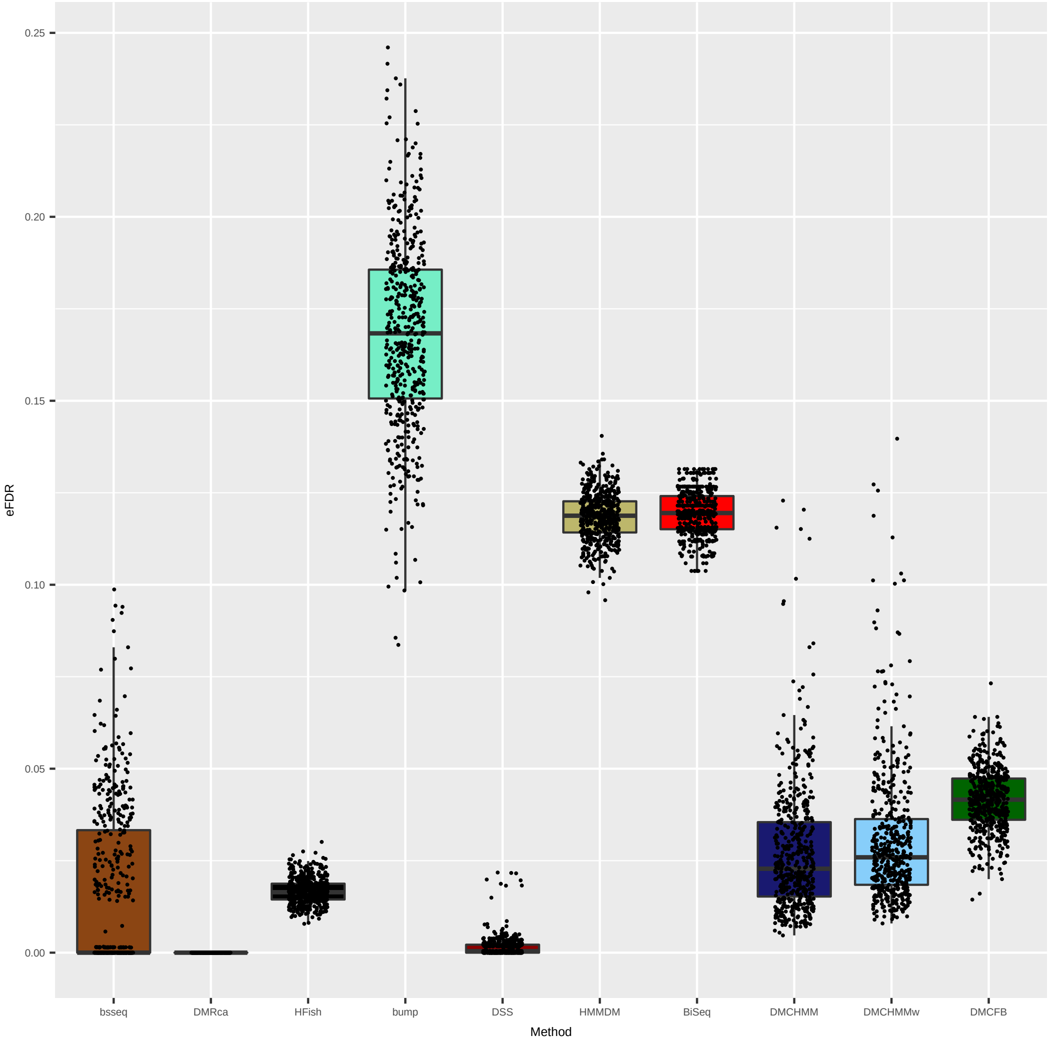

Figure S12. Average empirical FDR for different methods in the first scenario; Errors are generated from $N(\mu = 0, \sigma = 0.18)$. The vertical axis is truncated.

S4.3. Figures and Tables of Simulation Setup Scenario 1, $\sigma = 0.24$

We have used a second set of simulation scenarios to assess the performance of our proposed method. In this section we present the results of the first scenario in which errors were generated from $N(0, 0.24)$ to add to methylation levels of G1 and G2 curves.

The average overall accuracy (ACC) and average overall modified accuracy (MACC) is plotted in Figure S13 for different methods. Figure S14 shows Cohen's Kappa values. The average 'Sensitivity' and the average '1-Specificity' are illustrated in Figure S15. The proportions of the times the start and the end of DMRs are identified are depicted in Figure S16. Finally, Figure S17 shows the empirical FDR for different methods. In general, we see even better results with a larger noise in the data using DMCFB. This observation shows that our method is robust with respect to the variation in the data.

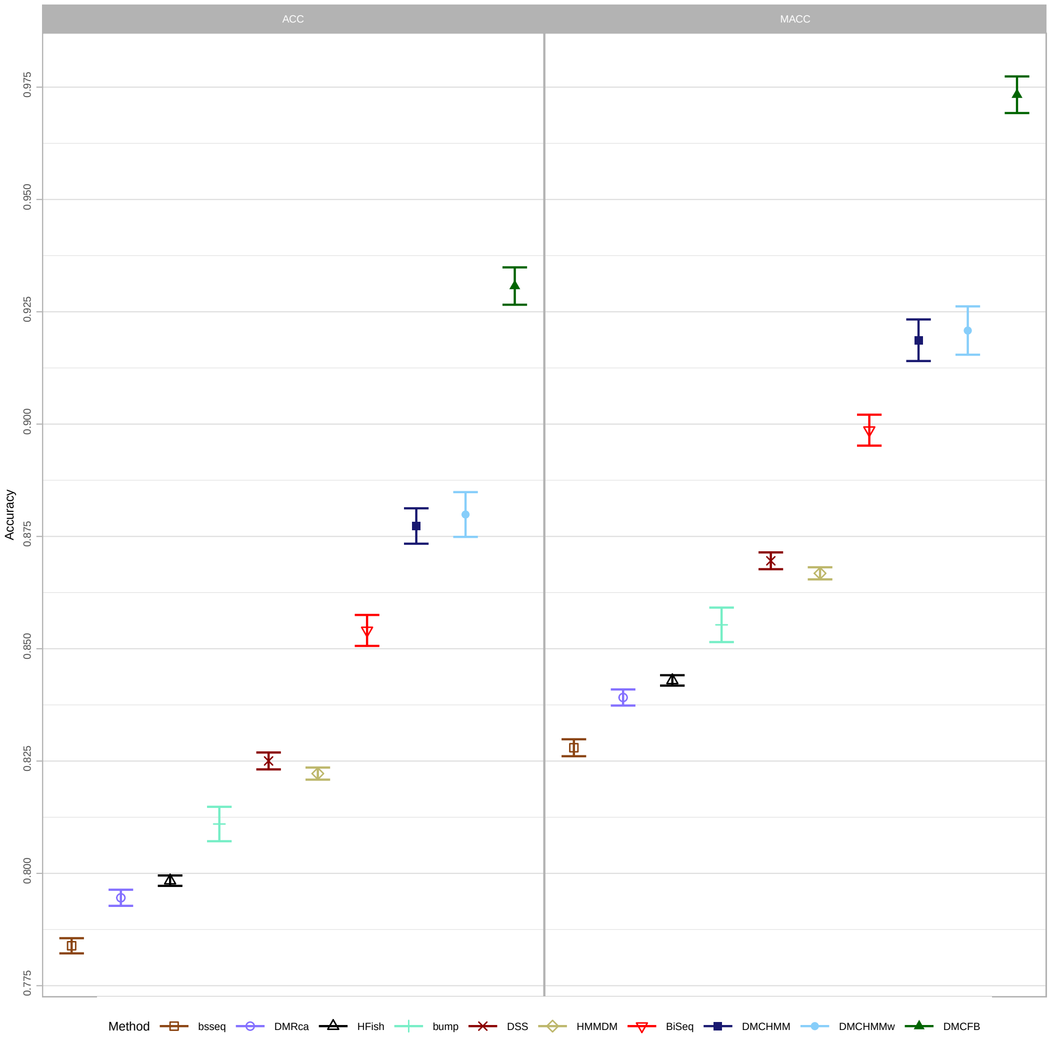

Figure S13. The average overall accuracy (ACC) and average overall modified accuracy (MACC) for different methods in simulated data for the first scenario; Errors are generated from $N(\mu = 0, \sigma = 0.24)$. The vertical axis is truncated.

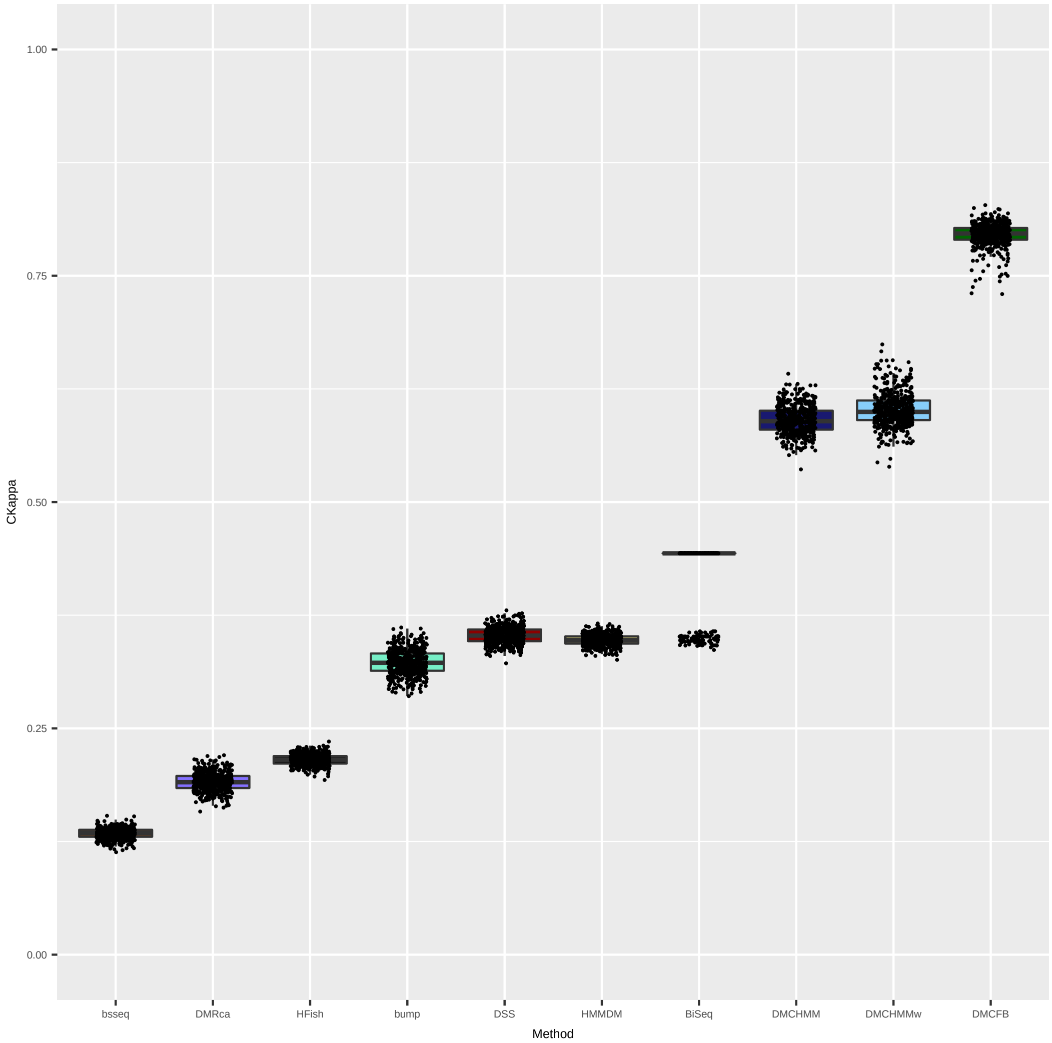

Figure S14. The box-plots of Cohen's Kappa for each method in simulated data for the first scenario; Errors are generated from $N(\mu = 0, \sigma = 0.24)$. The vertical axis is truncated.

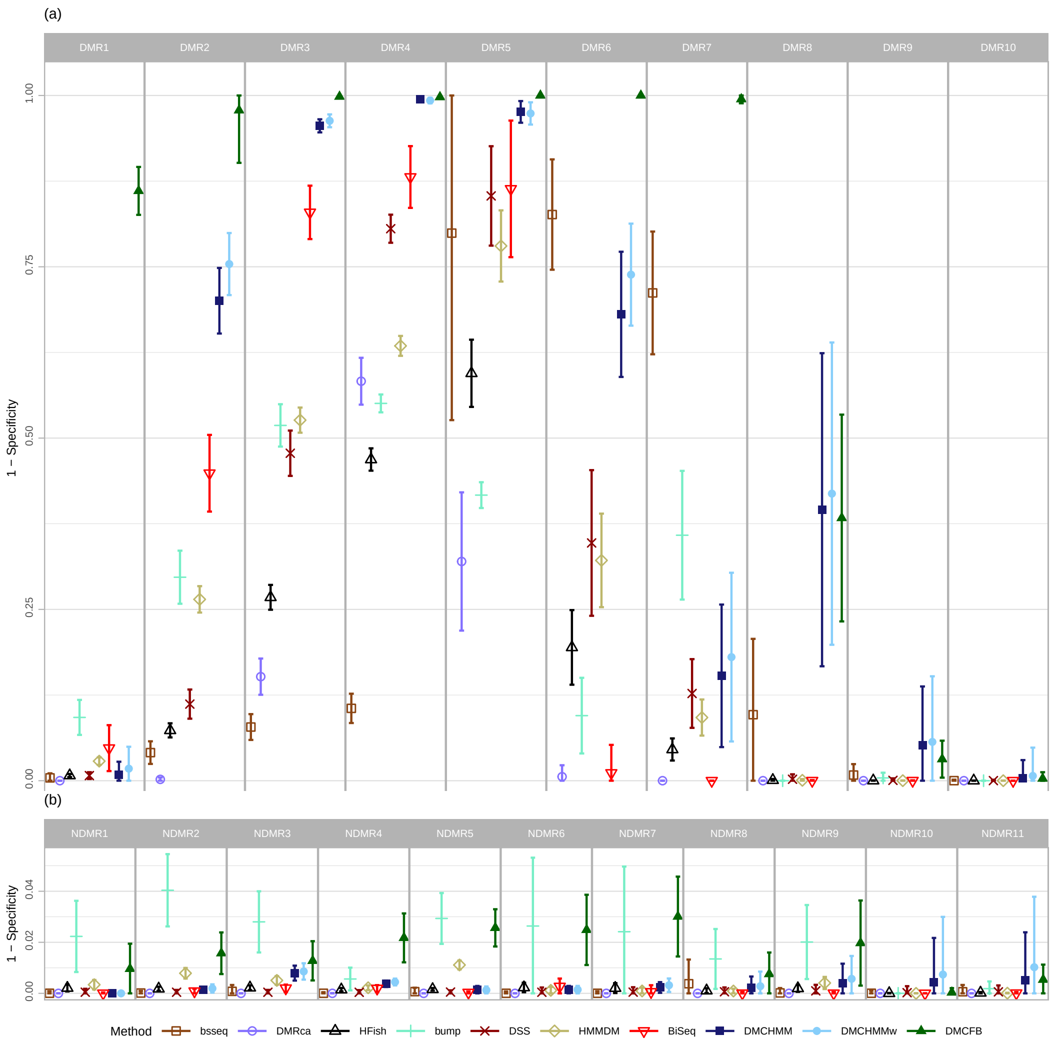

Figure S15. (a) The average proportion of correctly identified DMCs (Sensitivity) for each method separated by DMRs (sd error bars are added); (b) The average proportion of incorrectly identified DMCs (1 - Specificity) for each method separated by NDMRs (The axis is truncated) in simulated data for the first scenario; Errors are generated from $N(\mu = 0, \sigma = 0.24)$.

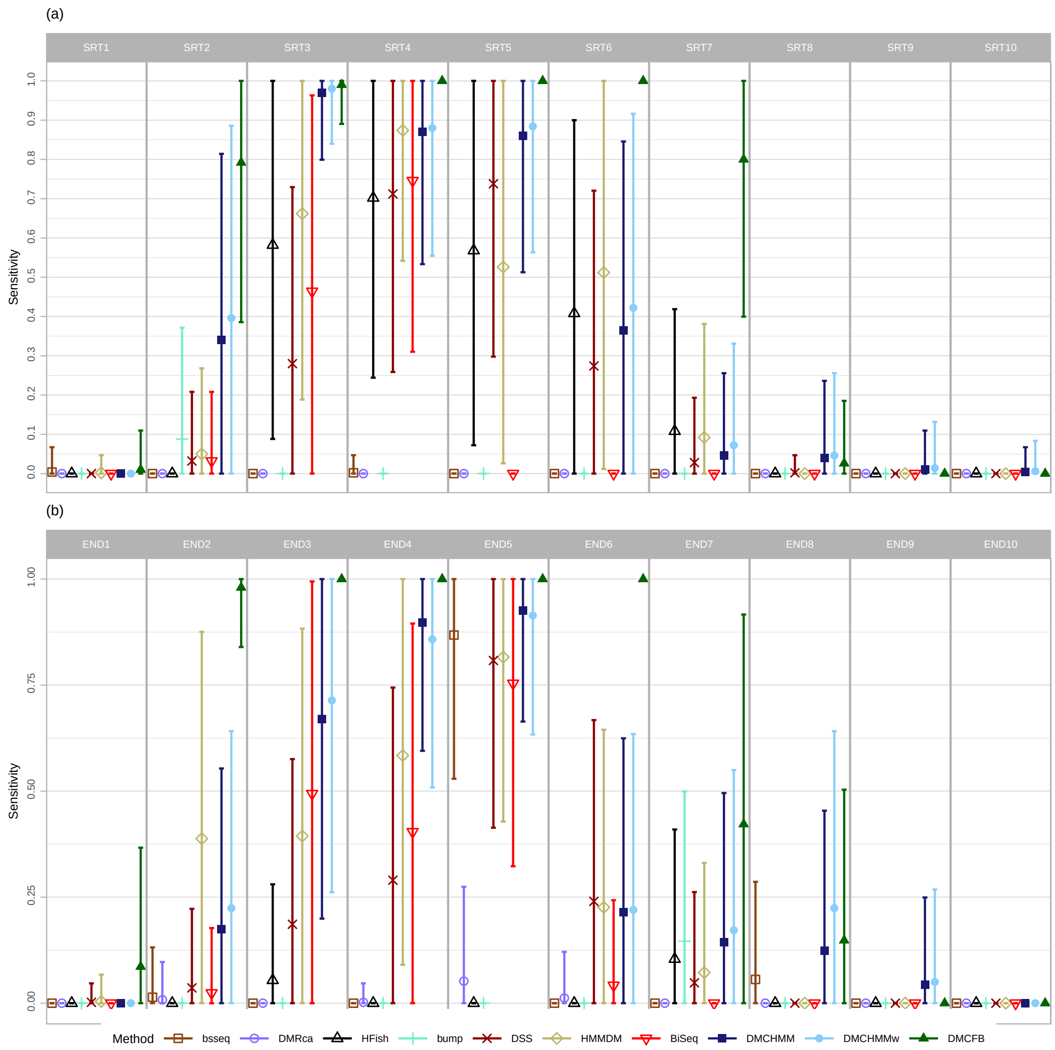

Figure S16. Average number of times that the start (a) and end (b) positions of DMRs are identified as DMCs for different methods in simulated data for the first scenario; Errors are generated from $N(\mu = 0, \sigma = 0.24)$.

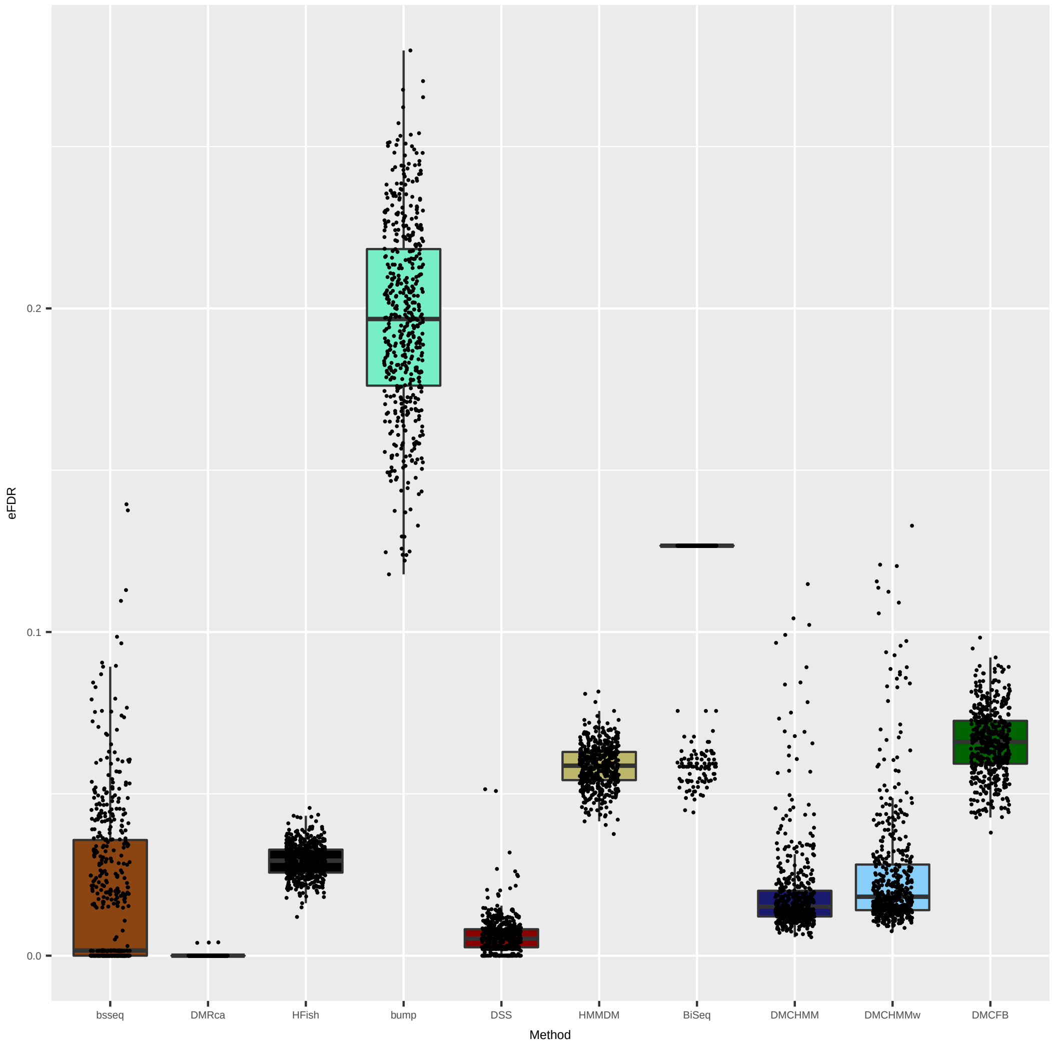

Figure S17. Average empirical FDR for different methods in simulated data for the first scenario; errors are generated from $N(\mu = 0, \sigma = 0.24)$. The vertical axis is truncated.

S4.4. Figures and Tables of Simulation Set-up Scenario 2, $\sigma = 0.18$

In this section we consider the second scenario given in Table S3 in Section S4.1 with DMRs of different lengths and locations to further assess the performance of our method.

The overall ACC and MACC are depicted in Figure S18. Comparison of methods in terms of the Cohen's Kappa criterion is provided in Figure S19. Figure S20 depicts 'Sensitivity' and '1 - Specificity' (and their standard deviation error-bar) for each method separated by DMRs and non-DMRs, respectively. The proportion of the start and the end of each DMR using different methods are shown in Figure S21. Finally, the empirical FDR for different methods is illustrated in Figure S22. All in all, the results are aligned with what we observed for the first simulation set-up.

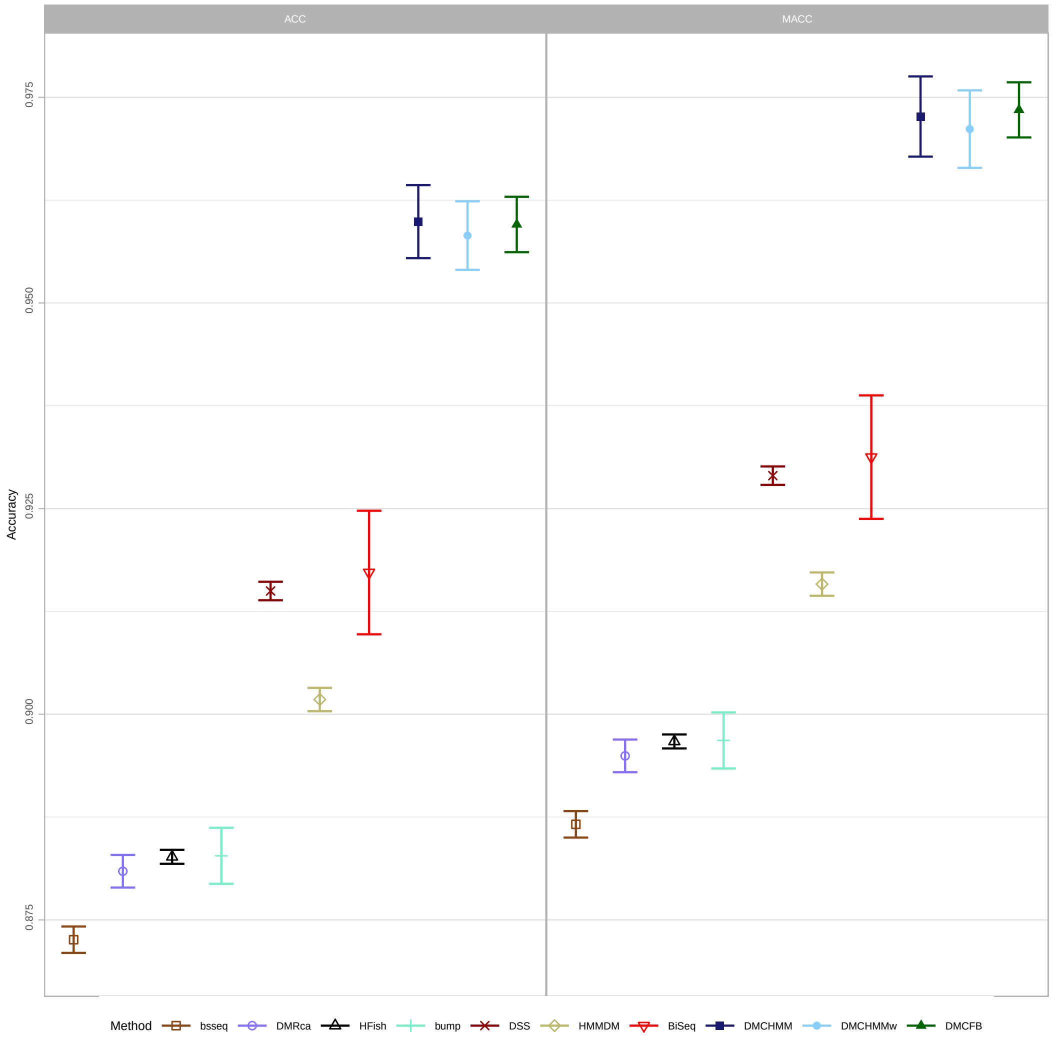

Figure S18. The average overall accuracy (ACC) and average overall modified accuracy (MACC) for different methods in simulated data for the second scenario; Errors are generated from $N(\mu = 0, \sigma = 0.18)$.

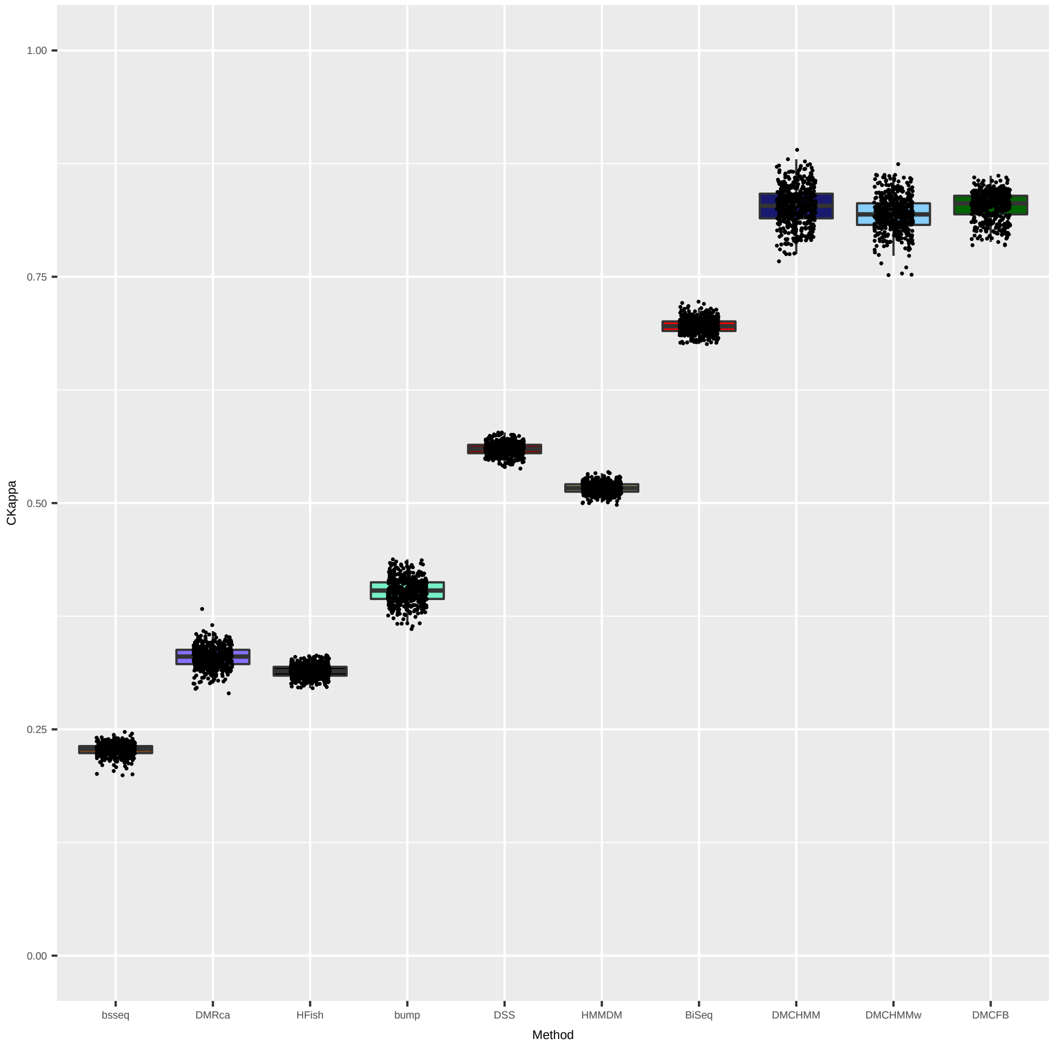

Figure S19. The box-plots of Cohen's Kappa for each method in simulated data for the second scenario; Errors are generated from $N(\mu = 0, \sigma = 0.18)$. The vertical axis is truncated.

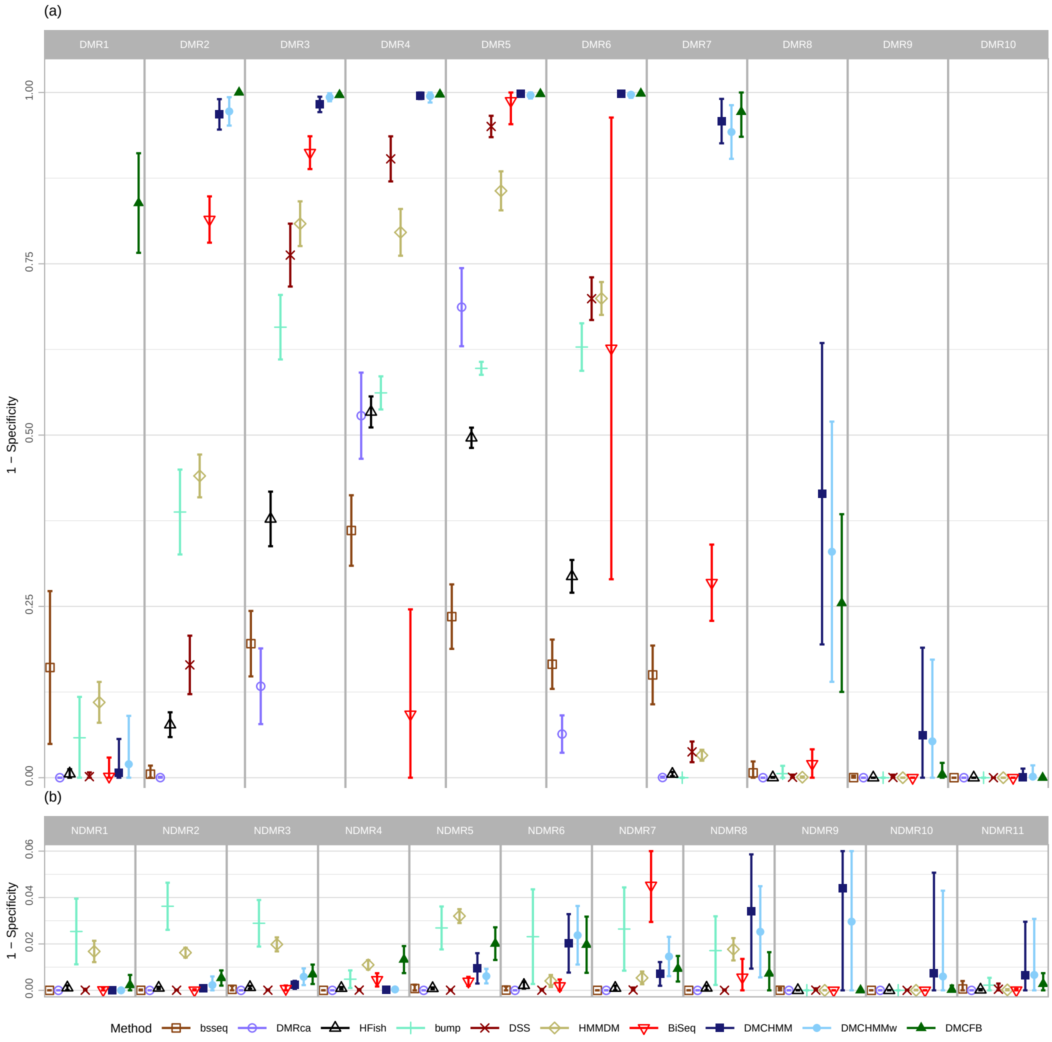

Figure S20. (a) The average proportion of correctly identified DMCs (Sensitivity) for each method separated by DMRs (sd error bars are added); (b) The average proportion of incorrectly identified DMCs (1 - Specificity) for each method separated by NDMRs (The axis is truncated) in simulated data for the second scenario; Errors are generated from $N(\mu = 0, \sigma = 0.18)$.

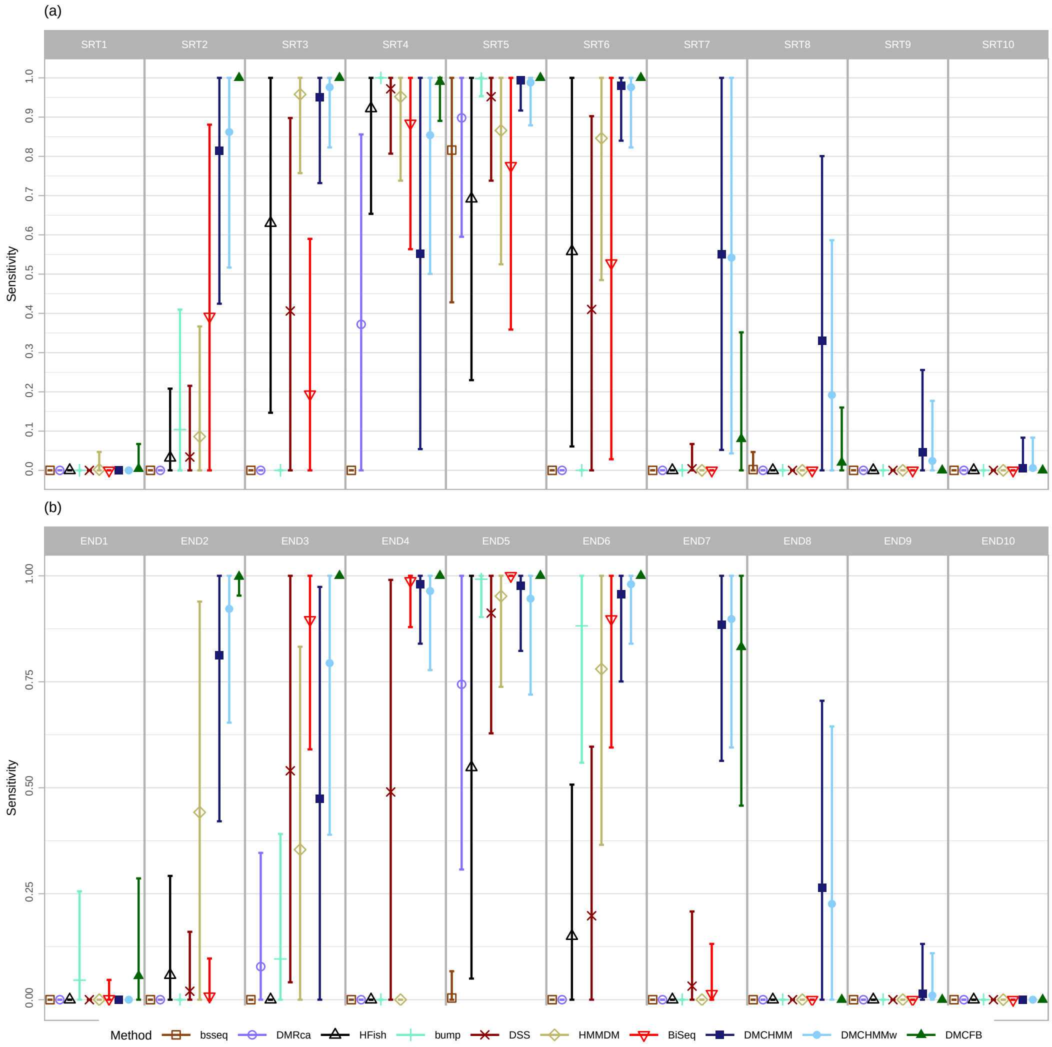

Figure S21. Average number of times that the start (a) and end (b) positions of DMRs are identified as DMCs for different methods in simulated data for the second scenario; Errors are generated from $N(\mu = 0, \sigma = 0.18)$.

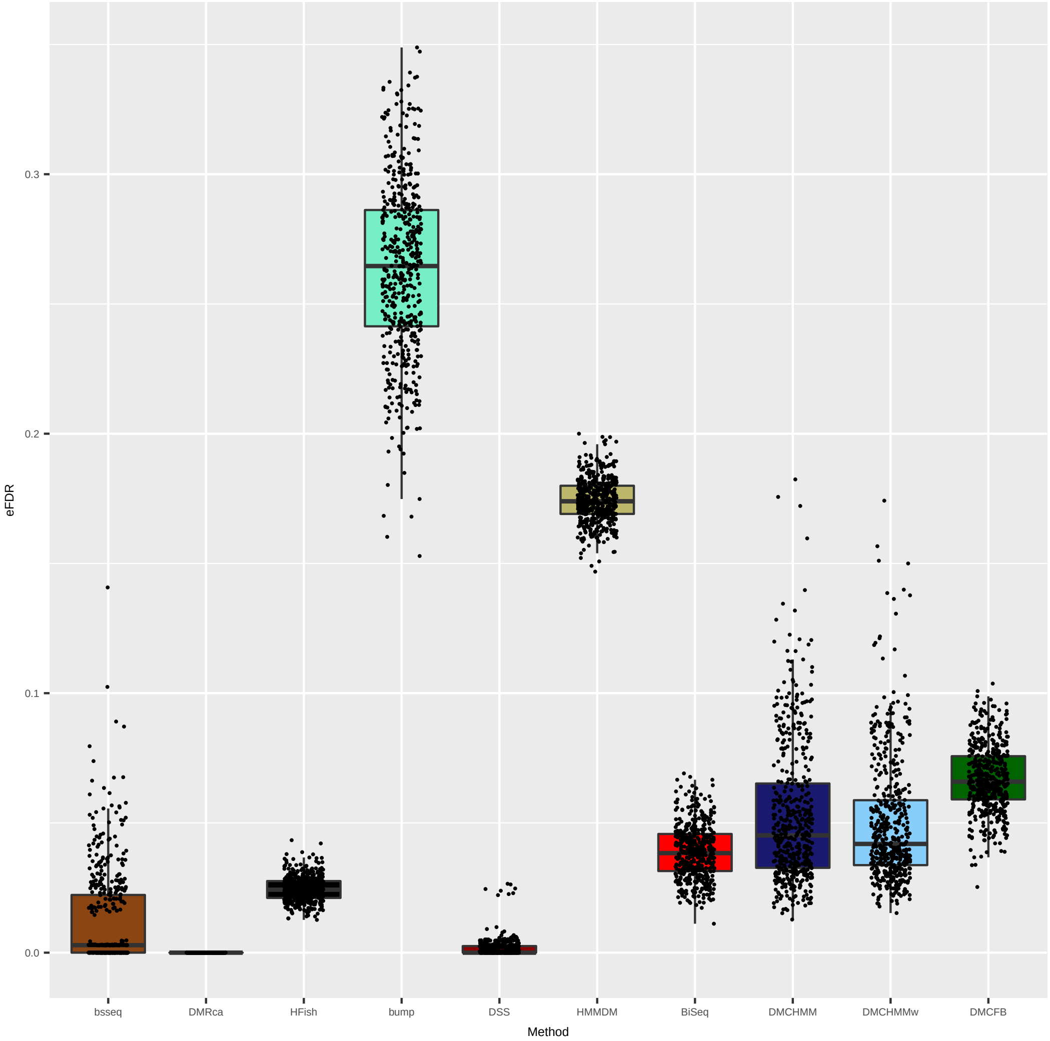

Figure S22. Average empirical FDR for different methods in simulated data for the second scenario; Errors are generated from $N(\mu = 0, \sigma = 0.18)$. The vertical axis is truncated.

S5. Sensitivity Analysis

Sensitivity analyses with respect to the choices of band-width and precision of prior are provided here.

S5.1. Sensitivity analysis with respect to band-width; Scenario 1, $\sigma = 0.18$

A set of band-width values in $\{20, 25, \ldots, 60\}$ is chosen for sensitivity analysis. In the following figures we provide the results.

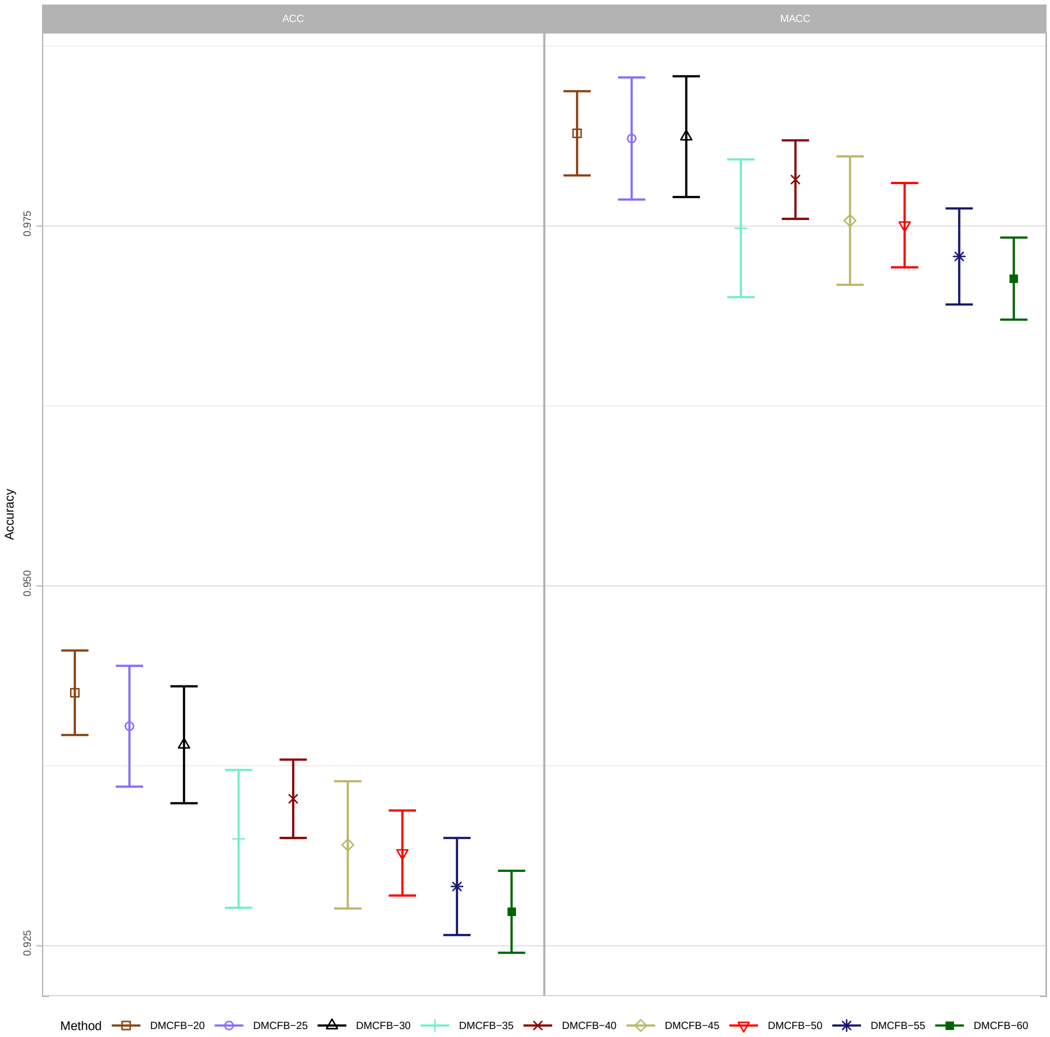

Figure S23. The average overall accuracy (ACC) and average overall modified accuracy (MACC) for each choice of band-width in `DMCFB` in simulated data for the first scenario; Errors are generated from $N(\mu = 0, \sigma = 0.18)$. The vertical axis is truncated.

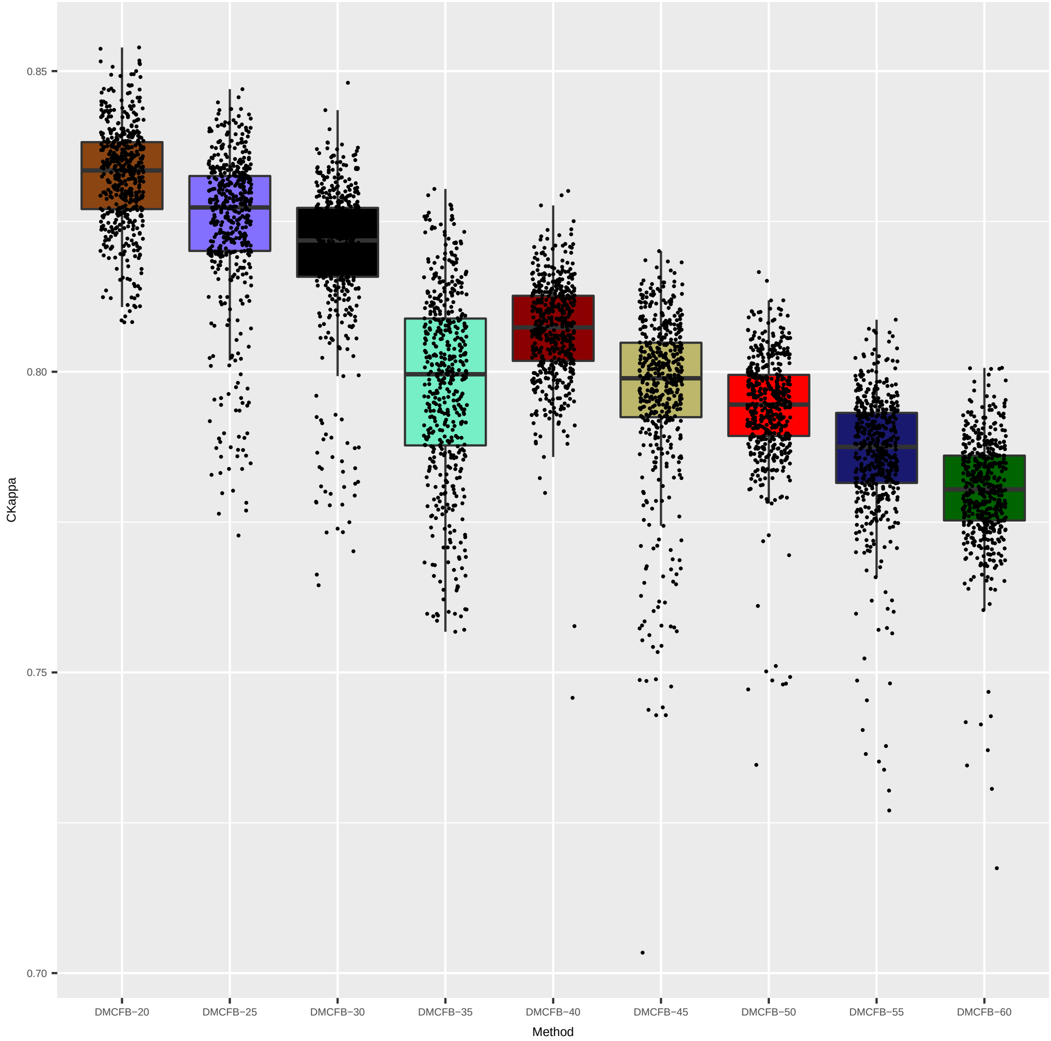

Figure S24. The box-plots of Cohen's Kappa for each choice of band-width in `DMCFB` in simulated data for the first scenario; Errors are generated from $N(\mu = 0, \sigma = 0.18)$. The vertical axis is truncated.

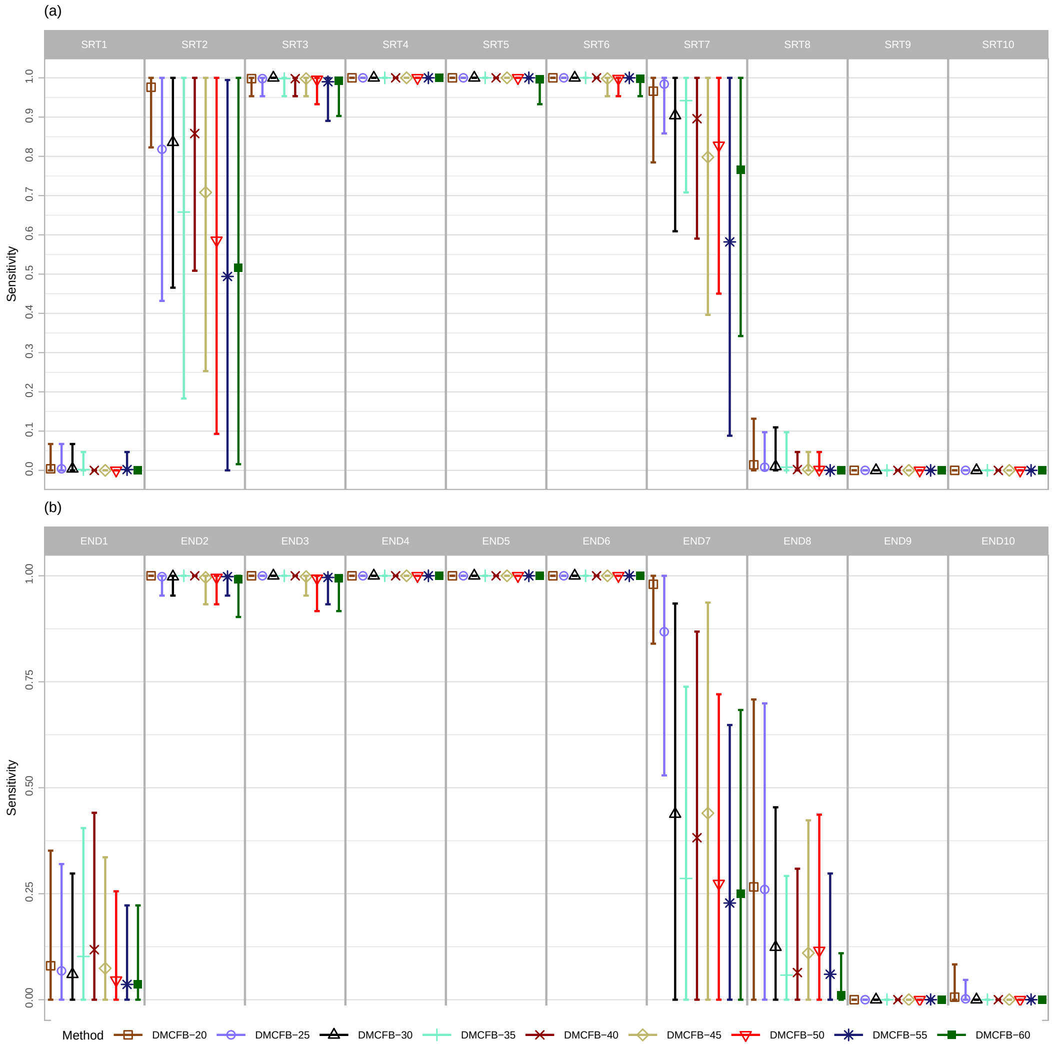

Figure S25. Average number of times that the start (a) and end (b) positions of DMRs are identified as DMCs for each choice of band-width in DMCFB in simulated data for the first scenario; Errors are generated from $N(\mu = 0, \sigma = 0.18)$.

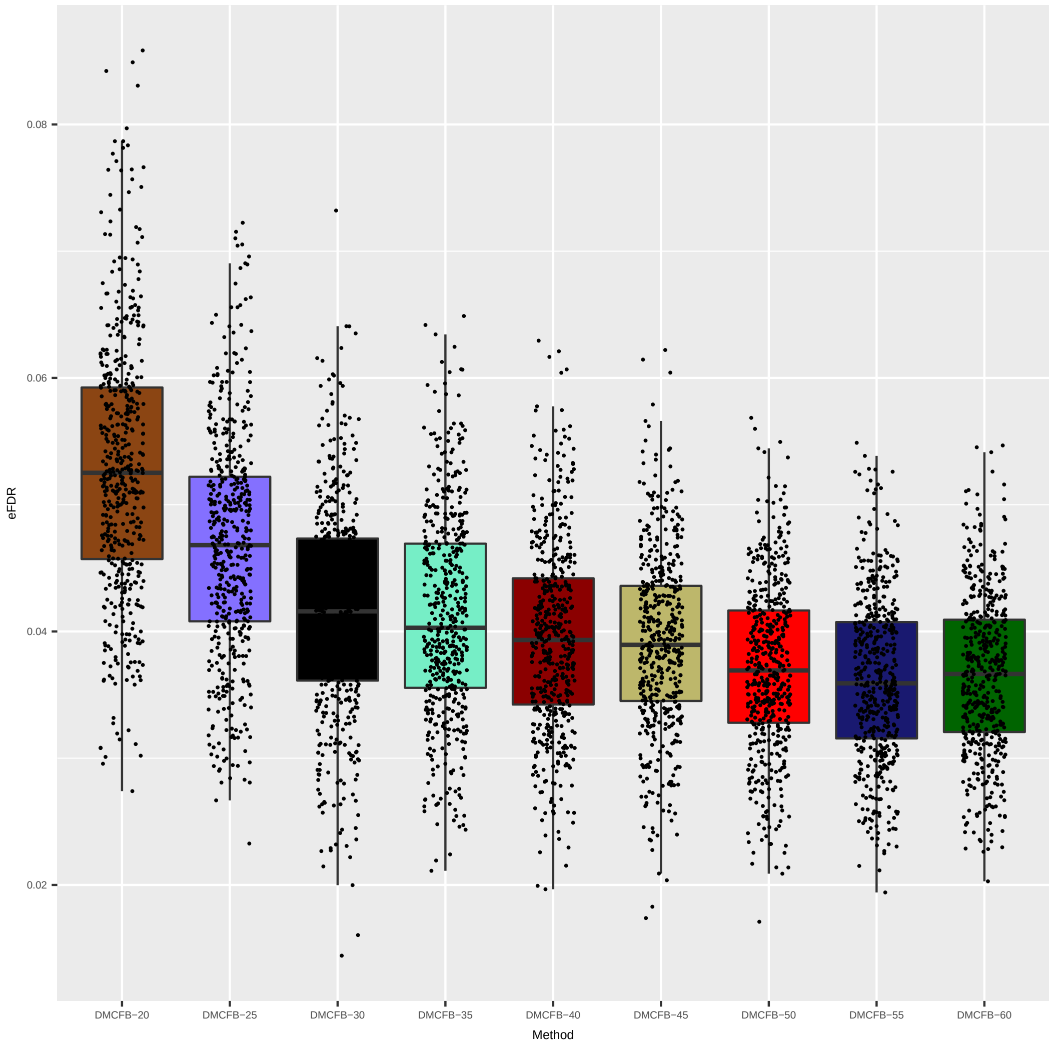

Figure S26. Average empirical FDR for each choice of band-width in DMCFB in simulated data for the first scenario; Errors are generated from $N(\mu = 0, \sigma = 0.18)$. The vertical axis is truncated.

S5.2. Sensitivity analysis with respect to band-width; Scenario 2, $\sigma = 0.18$

A set of band-width values in $\{20, 25, \ldots, 60\}$ is chosen for sensitivity analysis. In the following figures we provide the results.

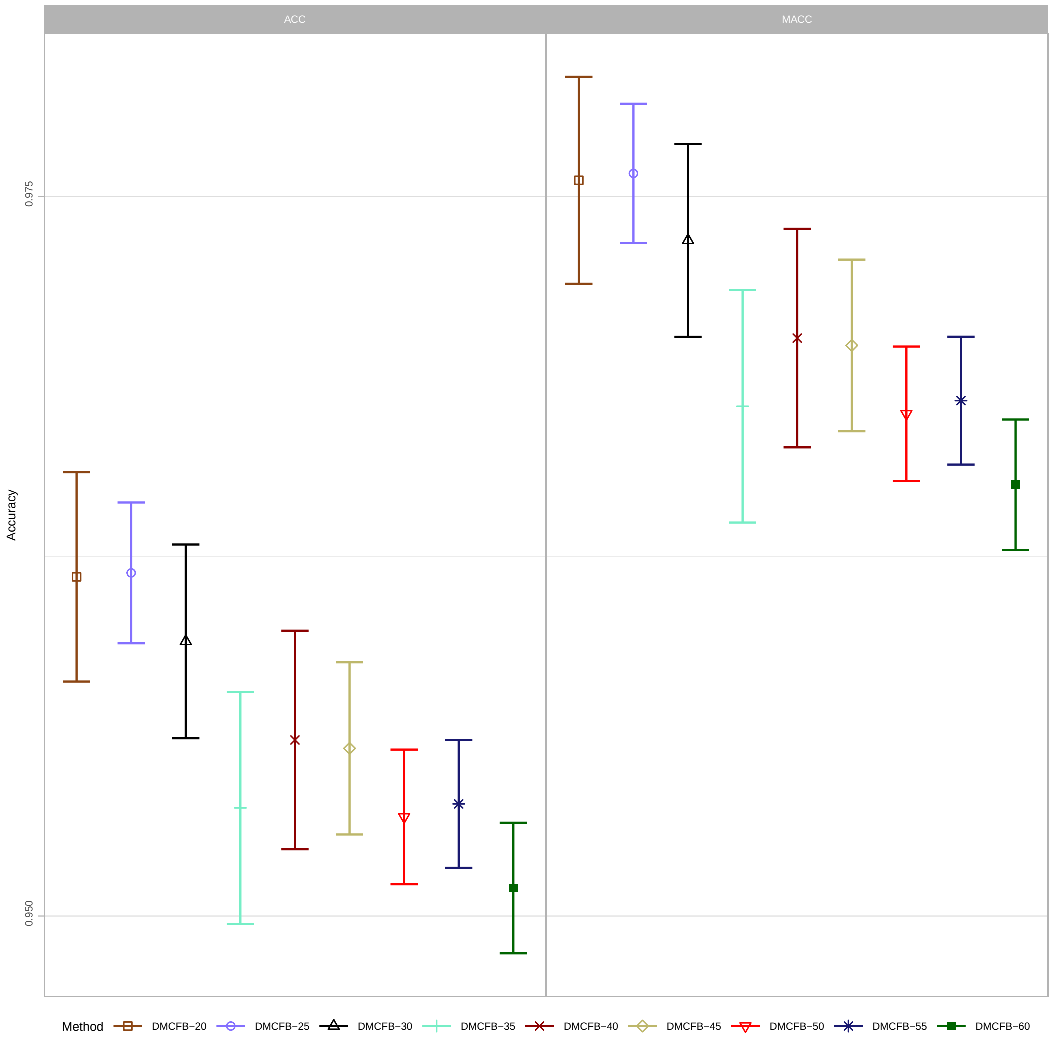

Figure S27. The average overall accuracy (ACC) and average overall modified accuracy (MACC) for each choice of band-width in `DMCFB` in simulated data for the second scenario; Errors are generated from $N(\mu = 0, \sigma = 0.18)$. The vertical axis is truncated.

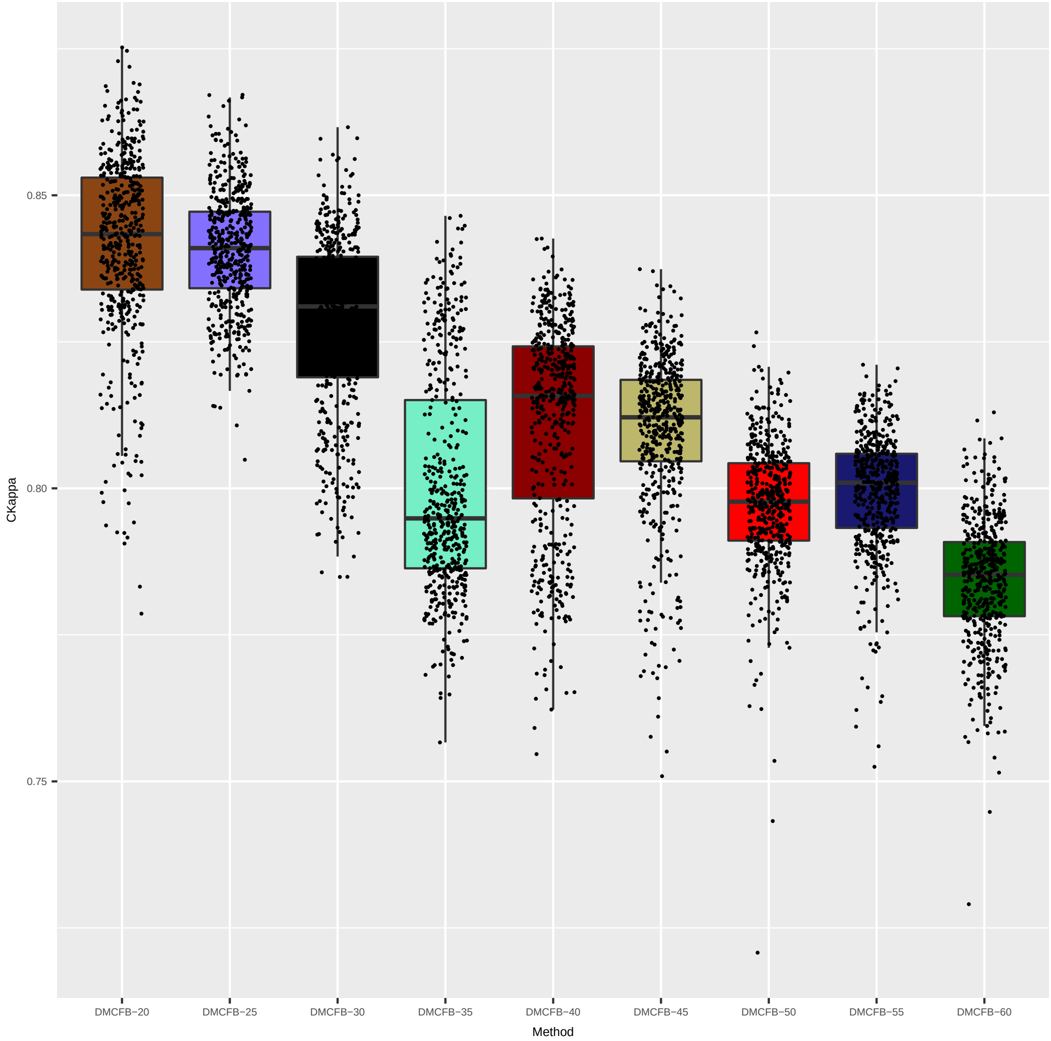

Figure S28. The box-plots of Cohen's Kappa for each choice of band-width in DMCFB in simulated data for the second scenario; Errors are generated from $N(\mu = 0, \sigma = 0.18)$. The vertical axis is truncated.

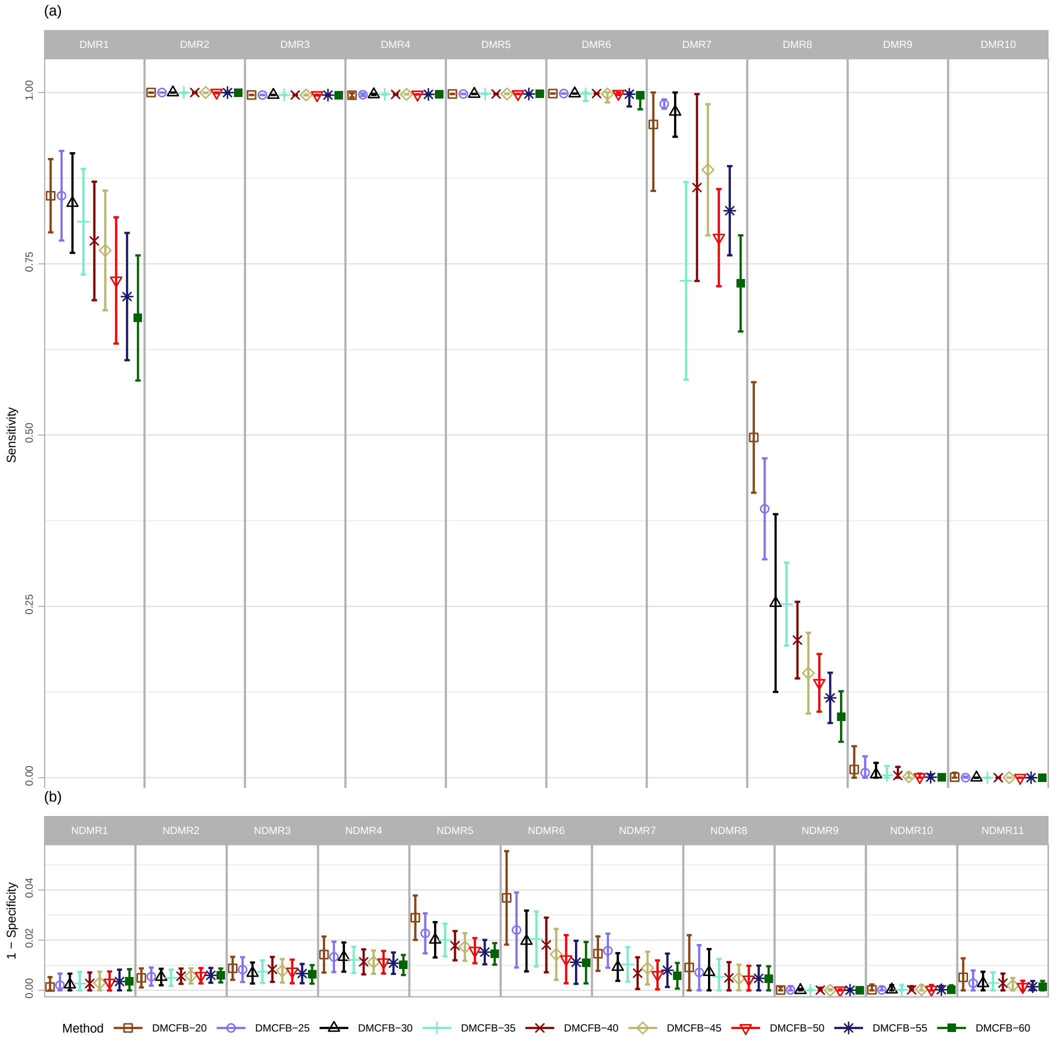

Figure S29. (a) The average proportion of correctly identified DMCs (Sensitivity) for each choice of band-width separated by DMRs (sd error bars are added); (b) The average proportion of incorrectly identified DMCs (1 - Specificity) for each choice of band-width separated by NDMRs (The axis is truncated) in `DMCFB` in simulated data for the second scenario; Errors are generated from $N(\mu = 0, \sigma = 0.18)$.

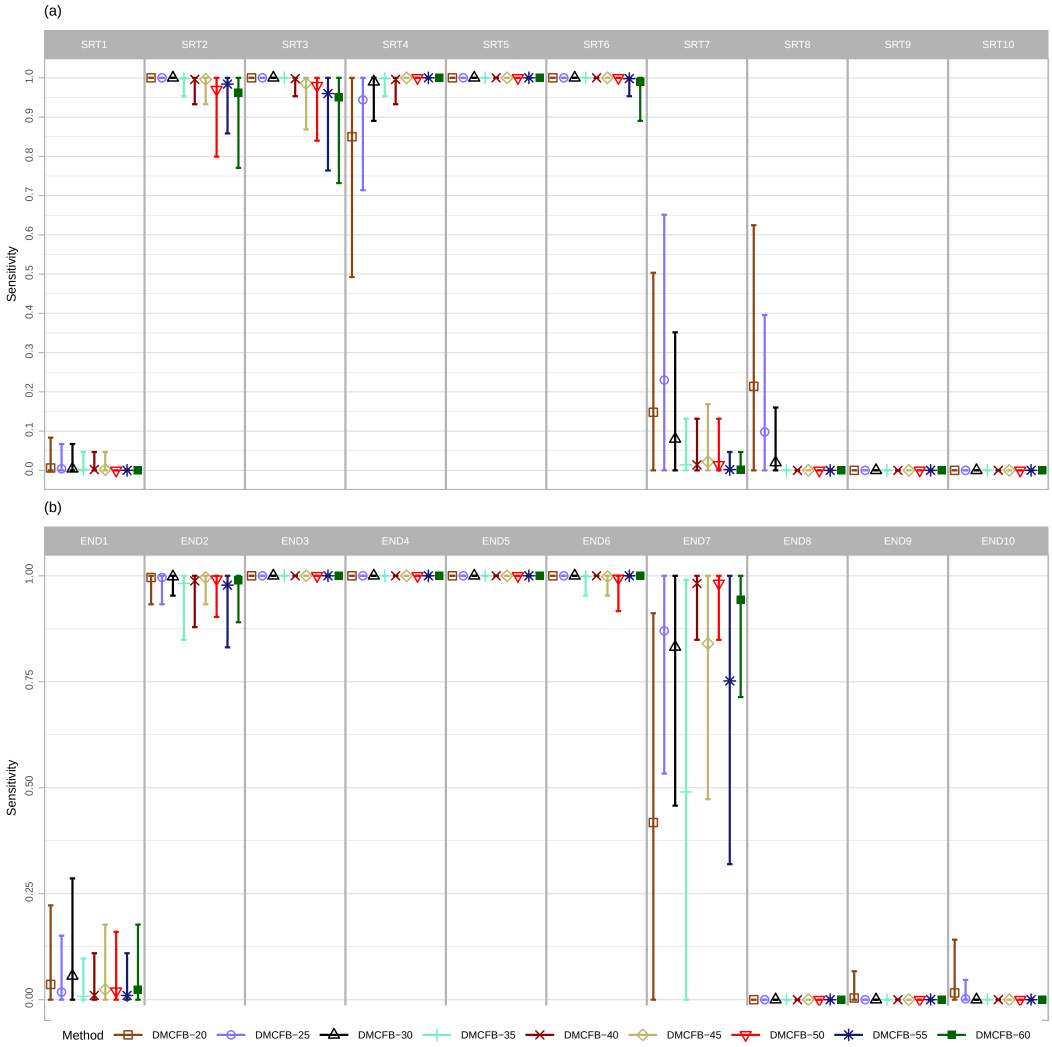

Figure S30. Average number of times that the start (a) and end (b) positions of DMRs are identified as DMCs for each choice of band-width in DMCFB in simulated data for the second scenario; Errors are generated from $N(\mu = 0, \sigma = 0.18)$.

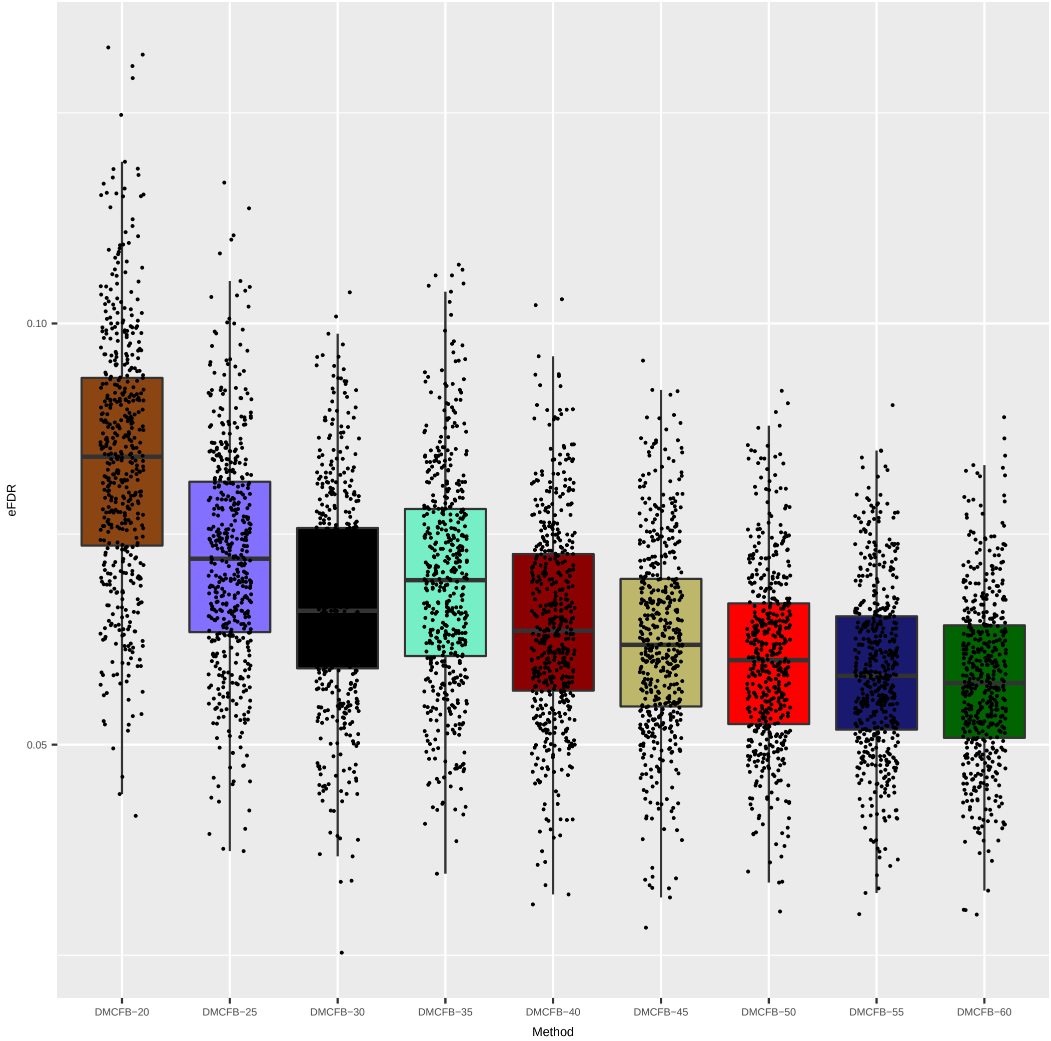

Figure S31. Average empirical FDR for each choice of band-width in `DMCFB` in simulated data for the second scenario; Errors are generated from $N(\mu = 0, \sigma = 0.18)$. The vertical axis is truncated.

S5.3. Sensitivity analysis with respect to prior precision; Scenario 1, $\sigma = 0.18$

A set of precision values in $\{30, 20, 10, 1/0.18, 1, 0.3, 0.2, 0.1\}$ is chosen for sensitivity analysis. In the following figures we provide the results.

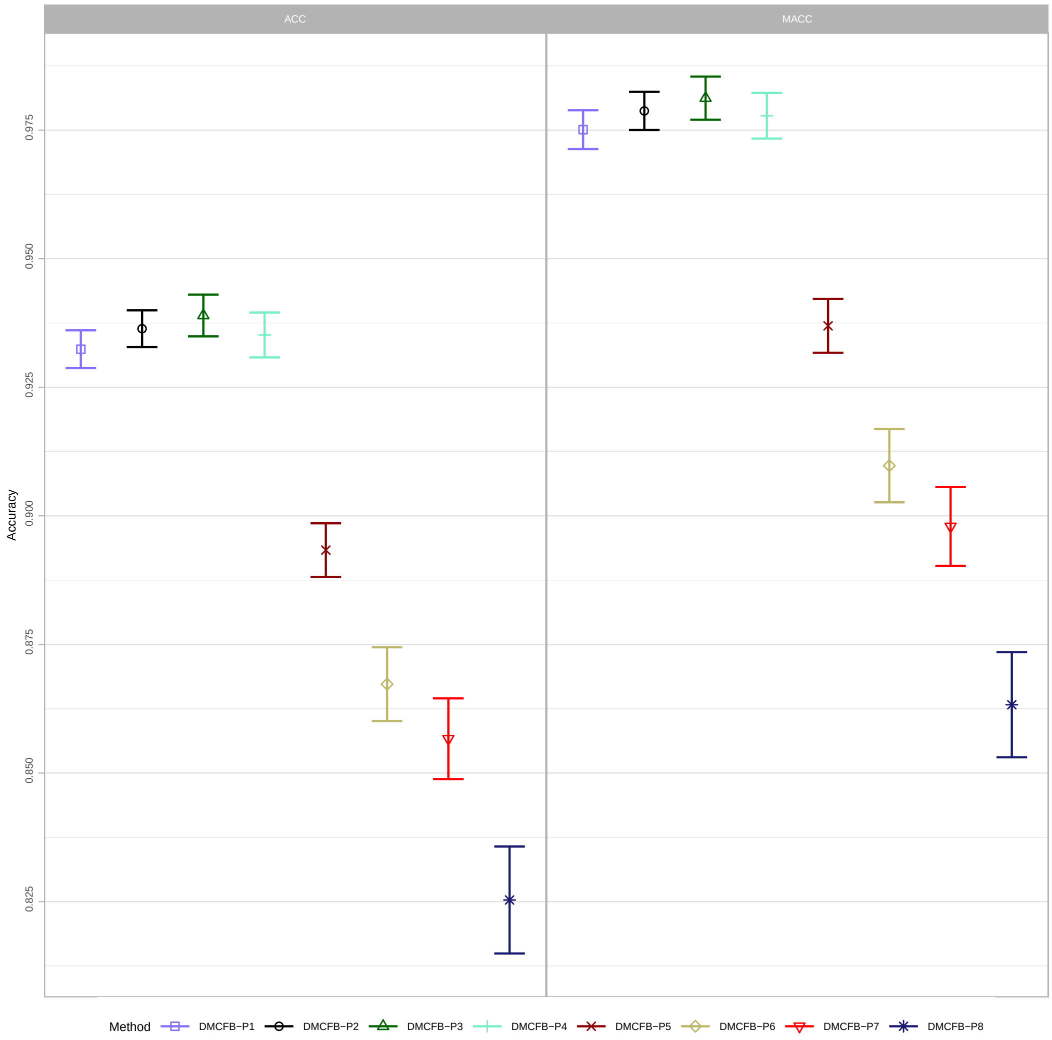

Figure S32. The average overall accuracy (ACC) and average overall modified accuracy (MACC) for each choice of precision in `DMCFB` in simulated data for the first scenario; Errors are generated from $N(\mu = 0, \sigma = 0.18)$. The vertical axis is truncated.

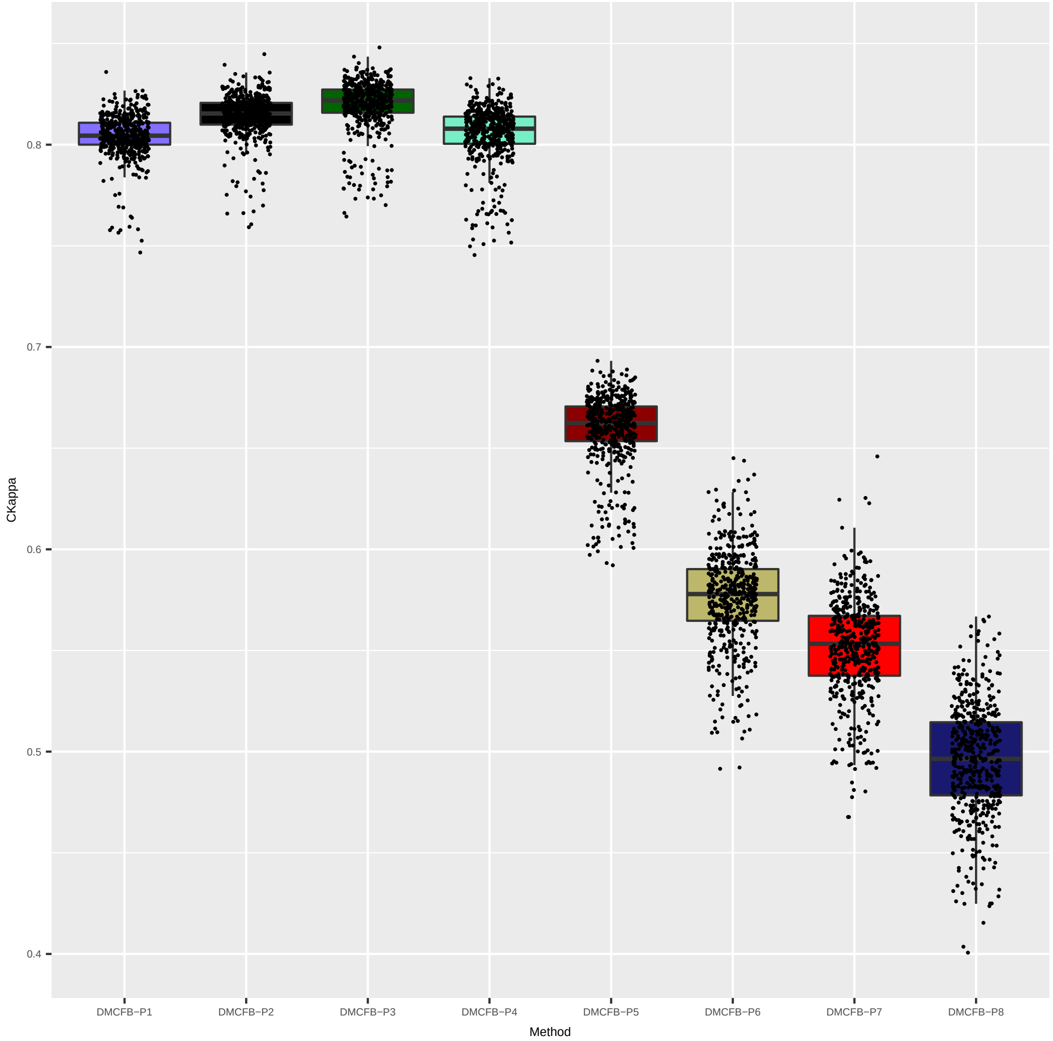

Figure S33. The box-plots of Cohen's Kappa for each choice of precision in `DMCFB` in simulated data for the first scenario; Errors are generated from $N(\mu = 0, \sigma = 0.18)$. The vertical axis is truncated.

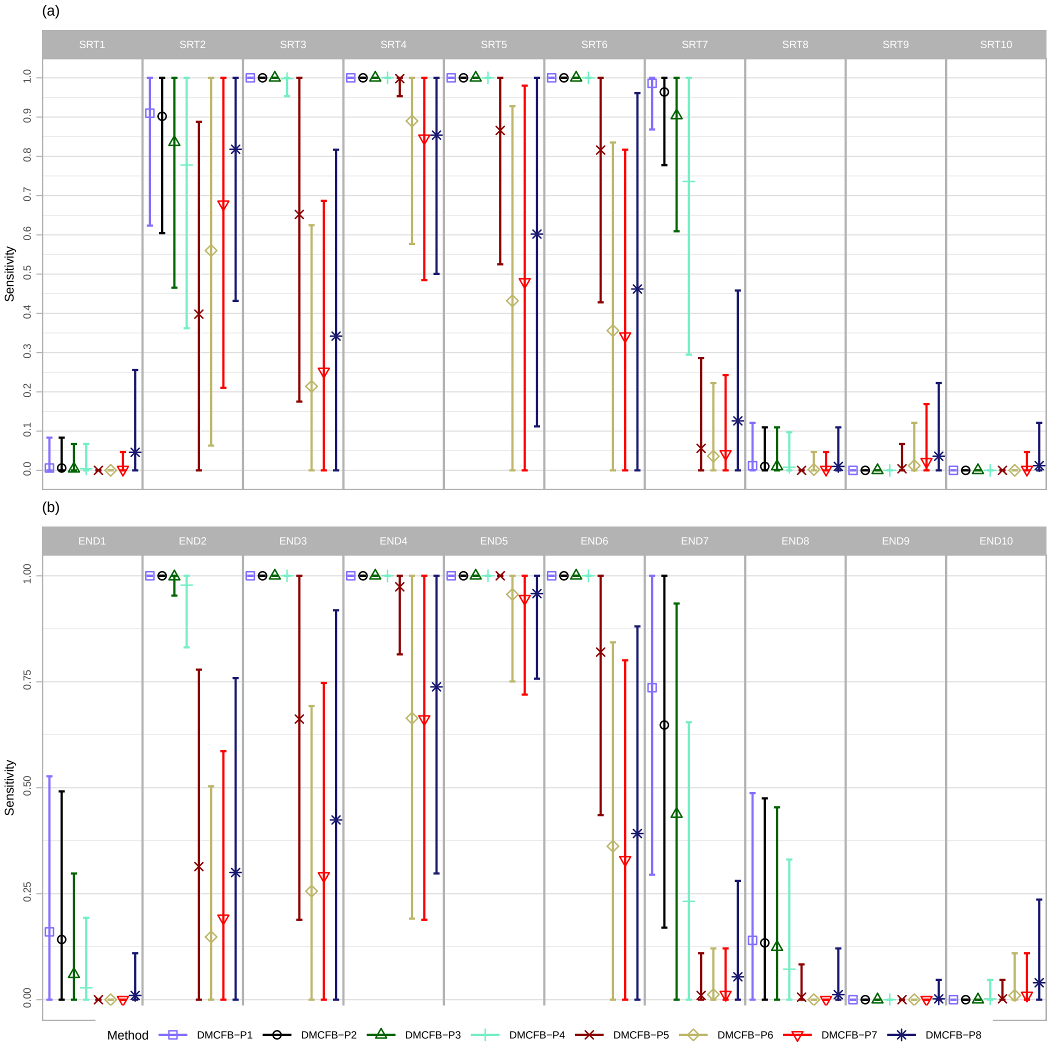

Figure S34. Average number of times that the start (a) and end (b) positions of DMRs are identified as DMCs for each choice of precision in DMCFB in simulated data for the first scenario; Errors are generated from $N(\mu = 0, \sigma = 0.18)$.

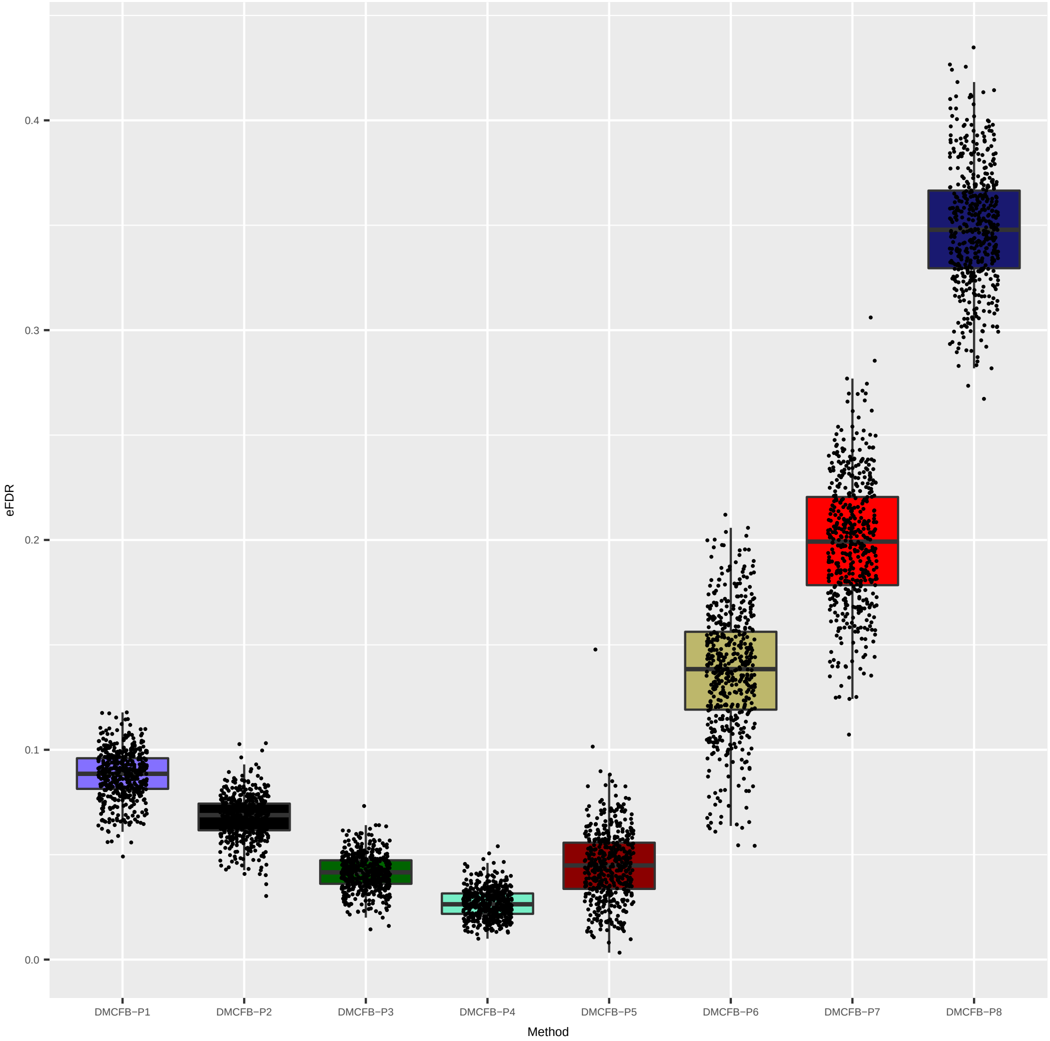

Figure S35. Average empirical FDR for each choice of precision in DMCFB in simulated data for the first scenario; Errors are generated from $N(\mu = 0, \sigma = 0.18)$. The vertical axis is truncated.

S5.4. Sensitivity analysis with respect to pair band-width and partition size; Scenario 1, $\sigma = 0.18$

Suppose a key region (with interesting associations with a grouping factor) is divided arbitrarily before the analysis. By re-assembling it afterwards, one may see or identify more clearly how a regulatory landscape is altered by the grouping factor. To study this we have chosen a set of pairs of band-width and partition size values as follows to assess its sensitivity in `DMCFB`. In the following table we provide the values.

Table S4. Simulation set-up parameters for the first scenario

Set	Band-width size	Partition size
1	30	500
2	45	750
3	60	1000
4	75	1250
5	90	1500
6	105	1750
7	120	2000
8	135	2250

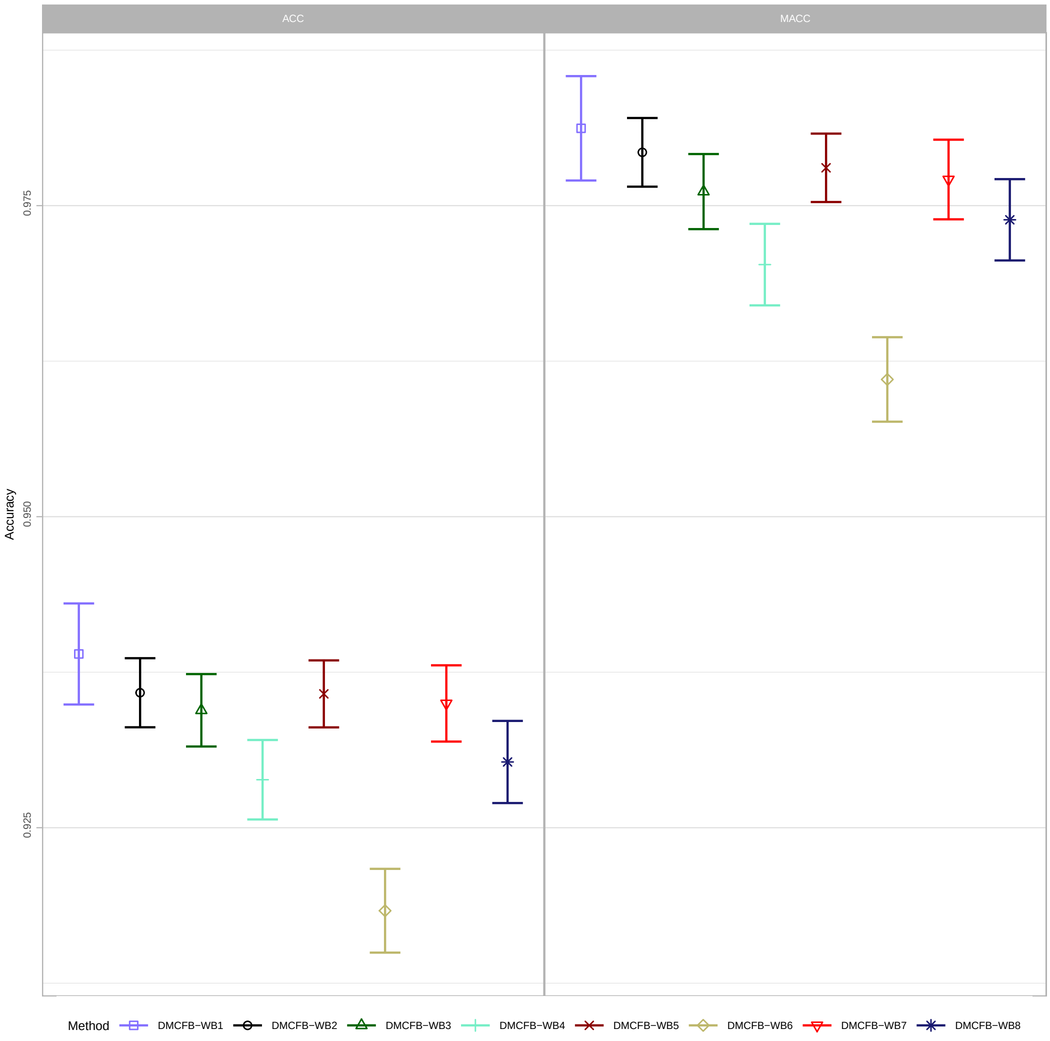

Figure S36. The average overall accuracy (ACC) and average overall modified accuracy (MACC) for each choice of joint band-width and partition size in `DMCFB` in simulated data for the first scenario; Errors are generated from $N(\mu = 0, \sigma = 0.18)$. The vertical axis is truncated.

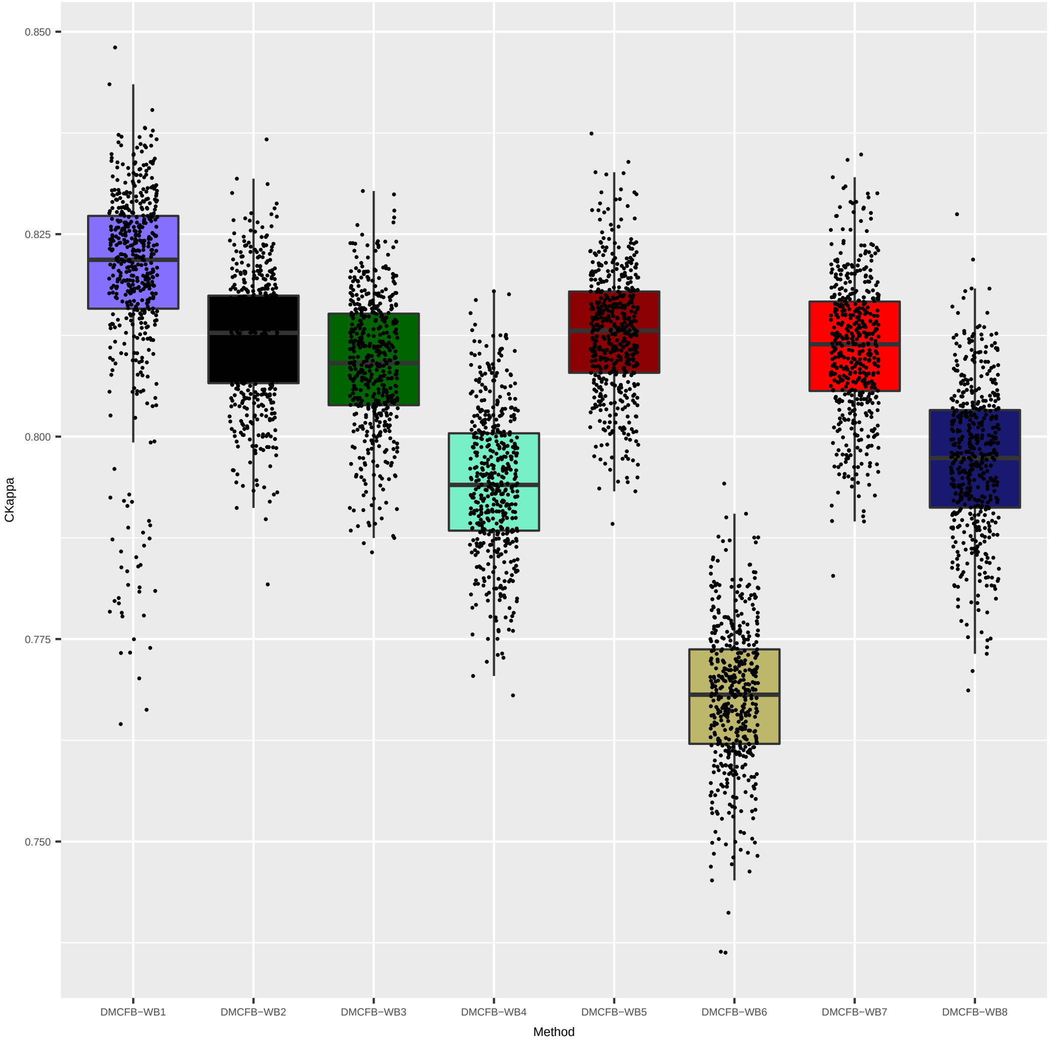

Figure S37. Box-plots of Cohen's Kappa for each choice of joint band-width and partition size in `DMCFB` in simulated data for the first scenario; Errors are generated from $N(\mu = 0, \sigma = 0.18)$. The vertical axis is truncated.

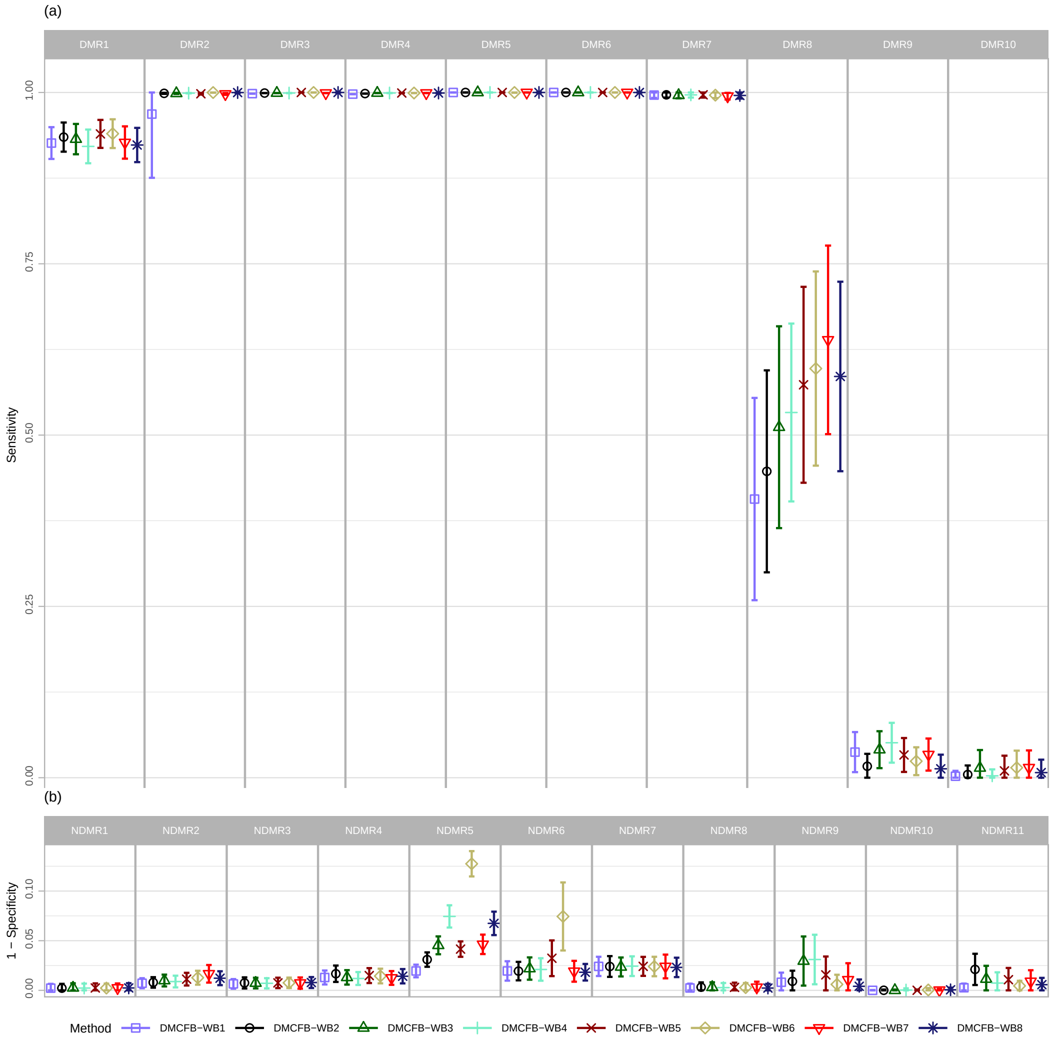

Figure S38. (a) The average proportion of correctly identified DMCs (Sensitivity) for each choice of joint band-width and partition size separated by DMRs (sd error bars are added); (b) The average proportion of incorrectly identified as DMCs (1 - Specificity) for each choice of joint band-width and partition size separated by NDMRs (The axis is truncated) in `DMCFB` in simulated data for the first scenario; Errors are generated from $N(\mu = 0, \sigma = 0.18)$.

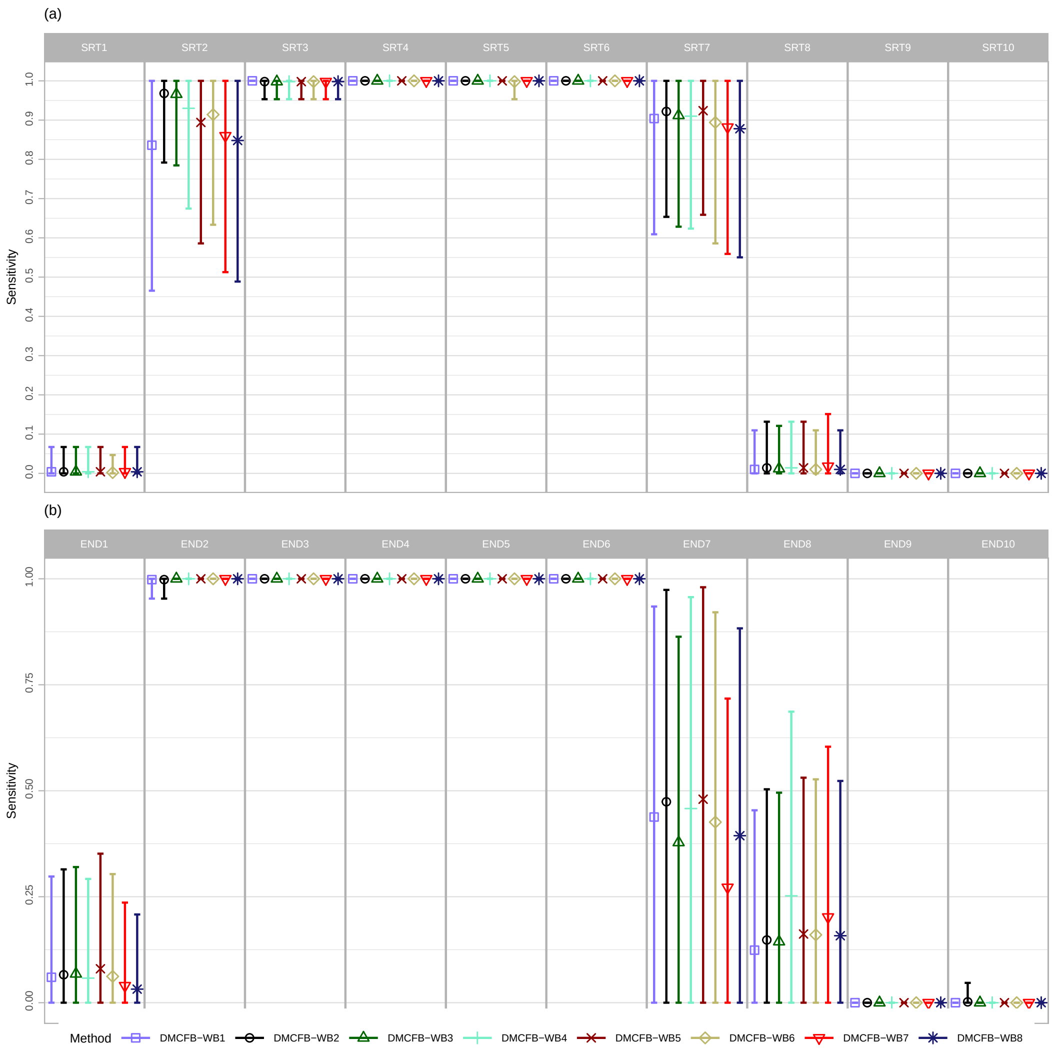

Figure S39. Average number of times that the start (a) and end (b) positions of DMRs are identified as DMCs for each choice of joint band-width and partition size in DMCFB in simulated data for the first scenario; Errors are generated from $N(\mu = 0, \sigma = 0.18)$.

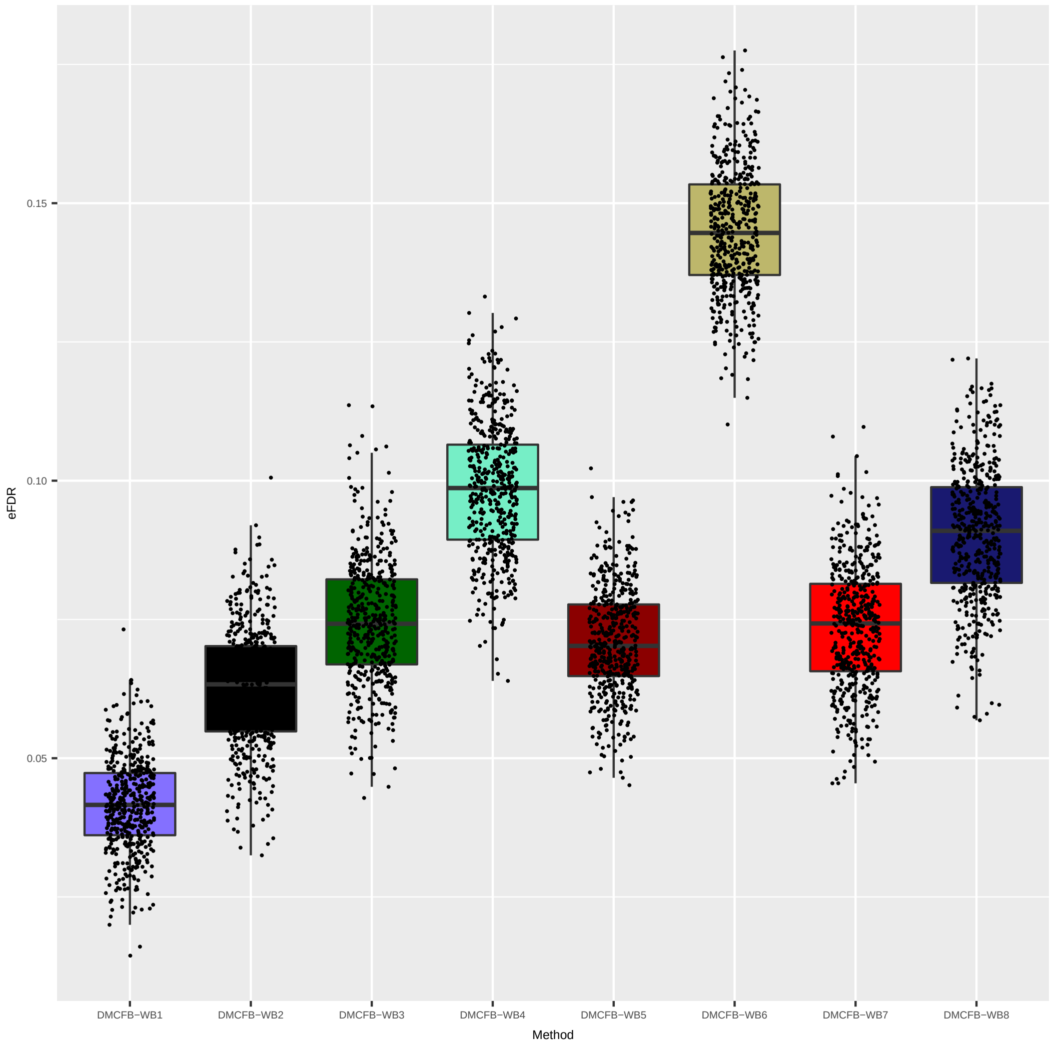

Figure S40. Average empirical FDR for each choice of joint band-width and partition size in DMCFB in simulated data for the first scenario; Errors are generated from $N(\mu = 0, \sigma = 0.18)$. The vertical axis is truncated.

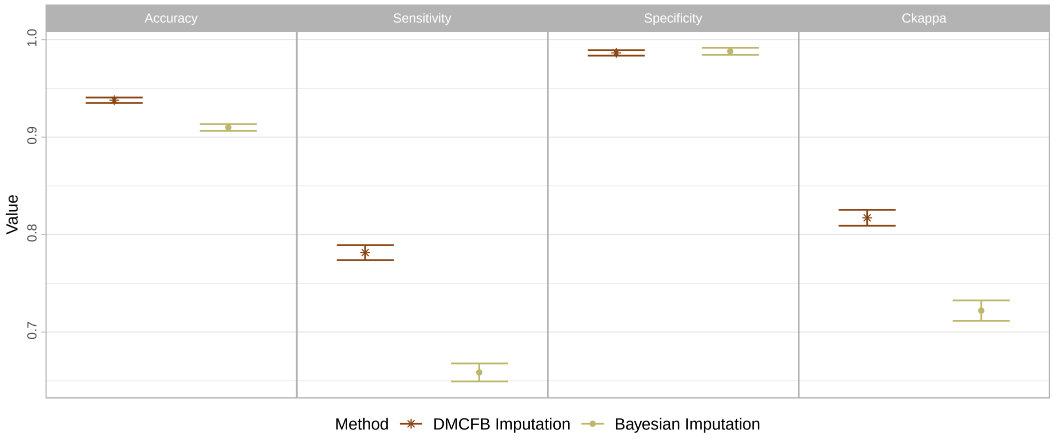

Figure S41. Average Accuracy, Sensitivity, 1-Specificity, and CKappa for imputing missing values using proposed method of setting $(y = 0, n = 0)$ versus regular Bayesian imputation in DMCFB in simulated data for the first scenario; Errors are generated from $N(\mu = 0, \sigma = 0.18)$.

S6. Comparison between DMCFB and SimBas methods in detecting DMCs

In this section, we compare the efficiency of DMCFB and SimBas method in DMC identification.

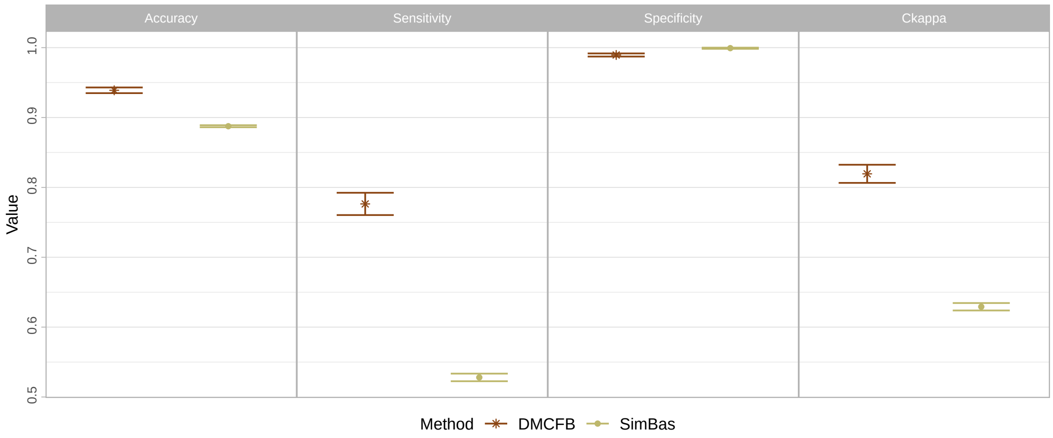

Figure S42. Comparison between DMCFB and SimBas in simulated data for the first scenario; Errors are generated from $N(\mu = 0, \sigma = 0.18)$. The vertical axis is truncated.

S7. Analysis of the BLK data

We have compared methylation profiles between cell types near the *BLK* gene described in Section 6of the main paper using DMCFB. Figure S43 shows pairwise DMCs for cell-type comparisons for the whole *BLK* region. We presented a graph of a small segment near the *BLK* gene in the main paper. We can observe that Monocyte cells and T-cells are hypermethylated compared to B-cells. Also, T-cells are hypomethylated compared to Monocyte cells. This fact stands out for the promoter of the *BLK* gene.

We also analyzed the BLK data using different FDR levels (Table S5). The FDR level 5×10^{-2}, which is quite liberal, identifies 63% of CpGs as DMCs, while the two FDR levels 1×10^{-5} and 1×10^{-8} show quite identical results; i.e., 28% of positions are DMCs.

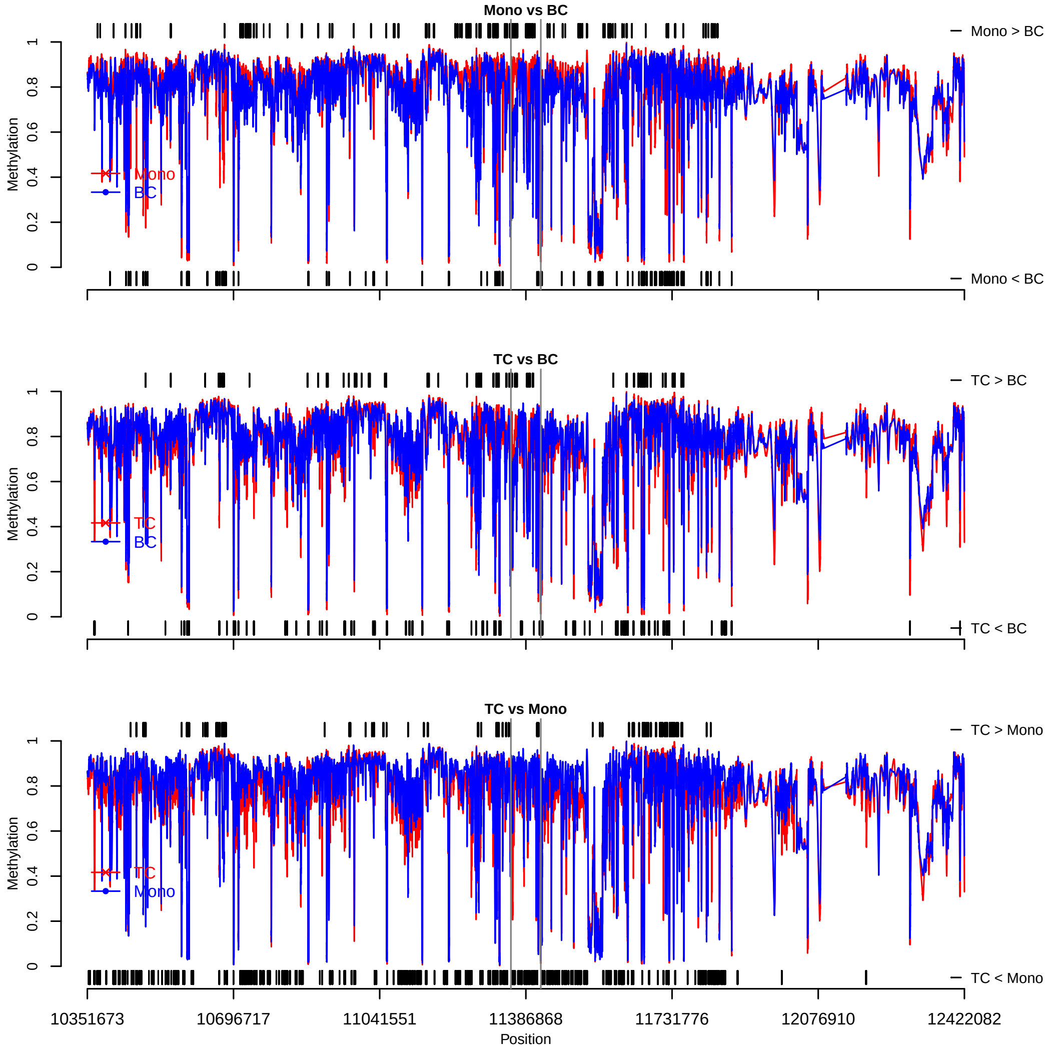

Figure S43. (a)-(c) Identified DMCs for three pairwise comparisons of cell-type methylation data near the *BLK* gene. Short black vertical lines indicate CpGs where one cell type was significantly different from the other, at credible interval level $\alpha = 1 \times 10^{-8}$. The average of methylation level for each cell-type are drawn using black and grey lines. B-cells are less methylated than other cell-types in the promoter of *BLK*.

Table S5. BLK data analysis on Chromosome 8 using DMCFB.

α	Pairwise contrasts		NDMC %	DMC %	Hypermethylated %	Hypomethylated %
5×10^{-2}	Mono	BC	60.06	39.94	23.43	16.51
	TC	BC	68.01	31.99	10.35	21.59
	TC	Mono	55.58	44.42	11.11	33.31
	All positions		37.41	62.59	–	–
1×10^{-5}	Mono	BC	86.34	13.66	5.95	7.71
	TC	BC	88.55	11.45	4.07	7.35
	TC	Mono	81.41	18.59	6.06	13.52
	All positions		71.33	28.67	–	–
1×10^{-8}	Mono	BC	86.42	13.57	5.89	7.68
	TC	BC	88.59	11.41	4.06	7.34
	TC	Mono	81.49	18.51	5.04	13.46
	All positions		71.41	28.59	–	–

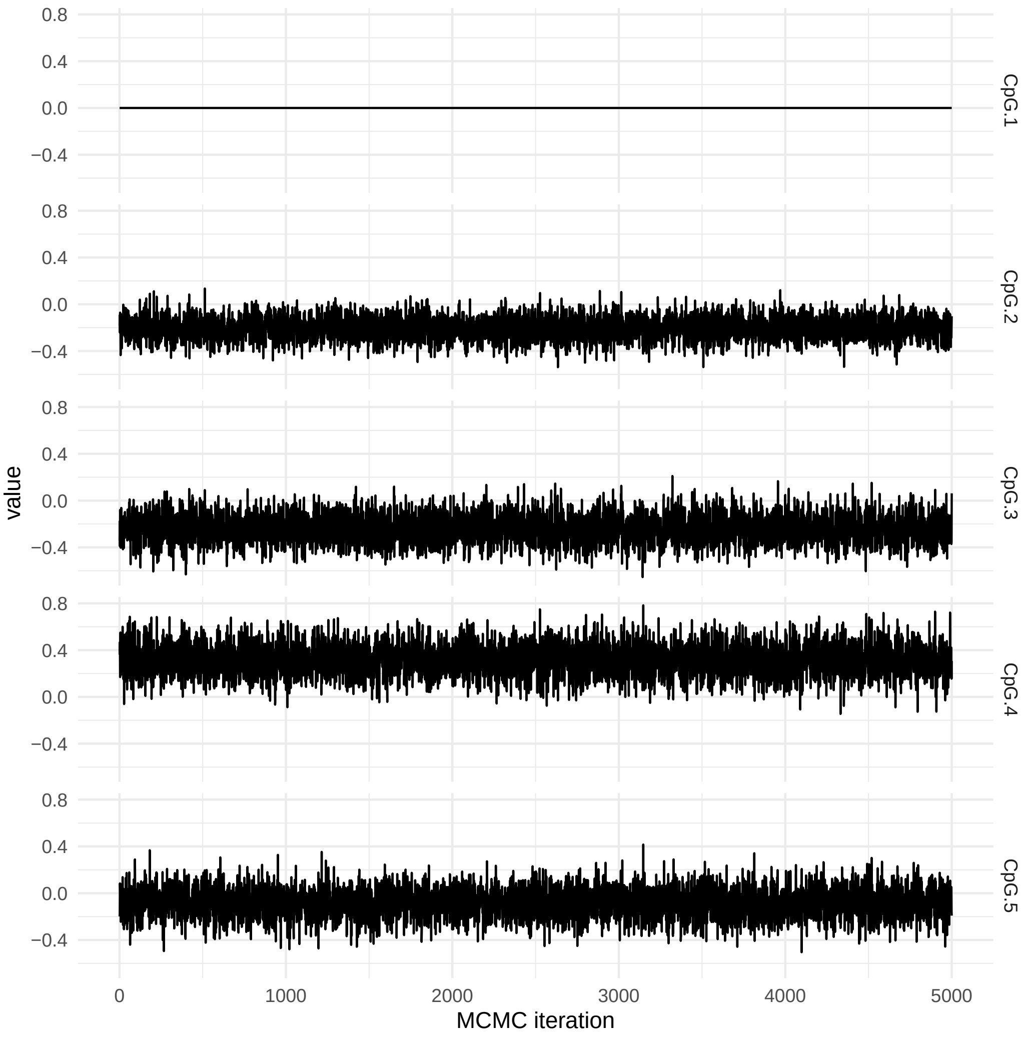

Figure S44. Trace plot of MCMC samples for a selected parameter of the model for five different genomic positions using DMCFB; BLK data.

S8. Analysis of the APL data

S8.1. Additional results from the reanalysis of APL data in the main paper

We reanalyzed the data set of patients with Acute Promyelocytic Leukemia (APL), published in Schoofs et al. (2013), publicly available on the Gene Expression Omnibus (accession no. GSE42119) in the main paper. These RRBS data contain 18 APL patients and 16 Control samples (which include 8 bone marrow samples from patients in remission, four healthy $CD34^+$ cells and four promyelocytes). APL is characterized by a chromosomal translocation involving the retinoic acid receptor-alpha gene on Chromosome 15 and 17 (Coombs et al., 2015).

In what follows, we present some of the findings discussed in the main paper.

Table S6. The percentage of CpGs identified as DMC by `DMCFB` and `BiSeq` displayed; Chromosomes 15 and 17.

Chromosome	DMCFB	BiSeq
15	30.64	6.48
17	31.90	3.67

Table S7. The number of CpGs identified as DMC by only `DMCFB`, only `BiSeq` and both methods at the same time, separated for CpG Islands and Shores; Chromosomes 15 and 17.

Chromosome	Location	DMCFB	BiSeq	Both
15	Islands	563	218	196
	Shores	718	196	170
17	Islands	1150	279	260
	Shores	1764	160	149

Table S8. Known binding sites identified as methylated by DMCFB and BiSeq; Chromosomes 15 and 17.

Chromosome	Start Position	End Position	Region ID	Gene Symbol	#DMCs by DMCFB	#DMCs by BiSeq
15	88578829	88579456	PR253	*CIB1 LOC390637*	3	0
	53487877	53488660	PR398	*CCPG1*	3	0
	76380719	76381874	PR495	*WDR61*	10	0
	70448192	70448955	PR847	*C15orf34*	4	0
	75500584	75501136	PR1239	*HMG20A*	24	0
	42815158	42815877	PR1262	*TRIM69*	1	0
	75499827	75500583	PR1719	*HMG20A*	20	3
	90196321	90197028	PR1920	*SLCO3A1*	2	0
	65621200	65622000	PR2000	*MAP2K5*	17	0
	90198507	90199221	PR2167	*SLCO3A1*	34	8
	41207073	41207569	PR2477	*TMEM62*	1	0
	60138953	60139520	PR2902	*NLF1 VPS13C*	2	0
17	29603024	29604938	PR172	*CCL2*	2	0
	25901212	25902428	PR326	*DKFZP434O047*	2	0
	45856072	45856714	PR401	*FLJ20920*	5	0
	78389194	78390110	PR501	*ZNF750*	18	0
	31152422	31153292	PR577	*MMP28 TAF15*	5	0
	200408	201496	PR723	*RPH3AL*	1	0
	77472015	77472905	PR835	*SIRT7 PCYT2*	1	0
	29717010	29717915	PR980	*CCL1*	30	5
	45859216	45859972	PR1046	*FLJ20920*	2	0
	29598889	29599671	PR1087	*CCL2*	4	0
	36717510	36718797	PR1125	NA	169	125
	21131431	21132190	PR1196	*MAP2K3*	16	0
	3570012	3570716	PR1467	*GSG2*	6	0
	2248484	2249021	PR1598	NA	5	0
	1533274	1533967	PR1606	*PRPF8*	2	0
	37589221	37589717	PR1611	*KCNH4 HCRT*	16	0
	37792477	37793256	PR1638	*STAT3*	12	0
	8031673	8032581	PR1663	*C17orf59*	7	0
	17679278	17680086	PR1746	*SREBF1*	2	0
	8138753	8139459	PR1821	*SLC25A35*	3	0
	1312519	1313086	PR1858	*CRK*	20	0
	8031211	8031672	PR2034	NA	10	0
	78154638	78155645	PR2323	NA	9	0
	27692272	27693059	PR2412	*RHOT1 C17orf75*	7	0
	21128960	21129724	PR2466	*MAP2K3*	7	0
	75384560	75385252	PR2479	*CBX8*	27	0
	9878588	9879133	PR2522	*GAS7*	5	0
	3814601	3815391	PR2611	*ATP2A3*	7	0
	9876834	9877589	PR2612	NA	8	0
	4789994	4790485	PR2775	*ENO3 SLC25A11*	6	0
	74441013	74441653	PR2777	*TIMP2*	7	0
	1568422	1568957	PR2892	*WDR81 MGC14376*	4	0
	39781024	39781679	PR2958	NA	5	0

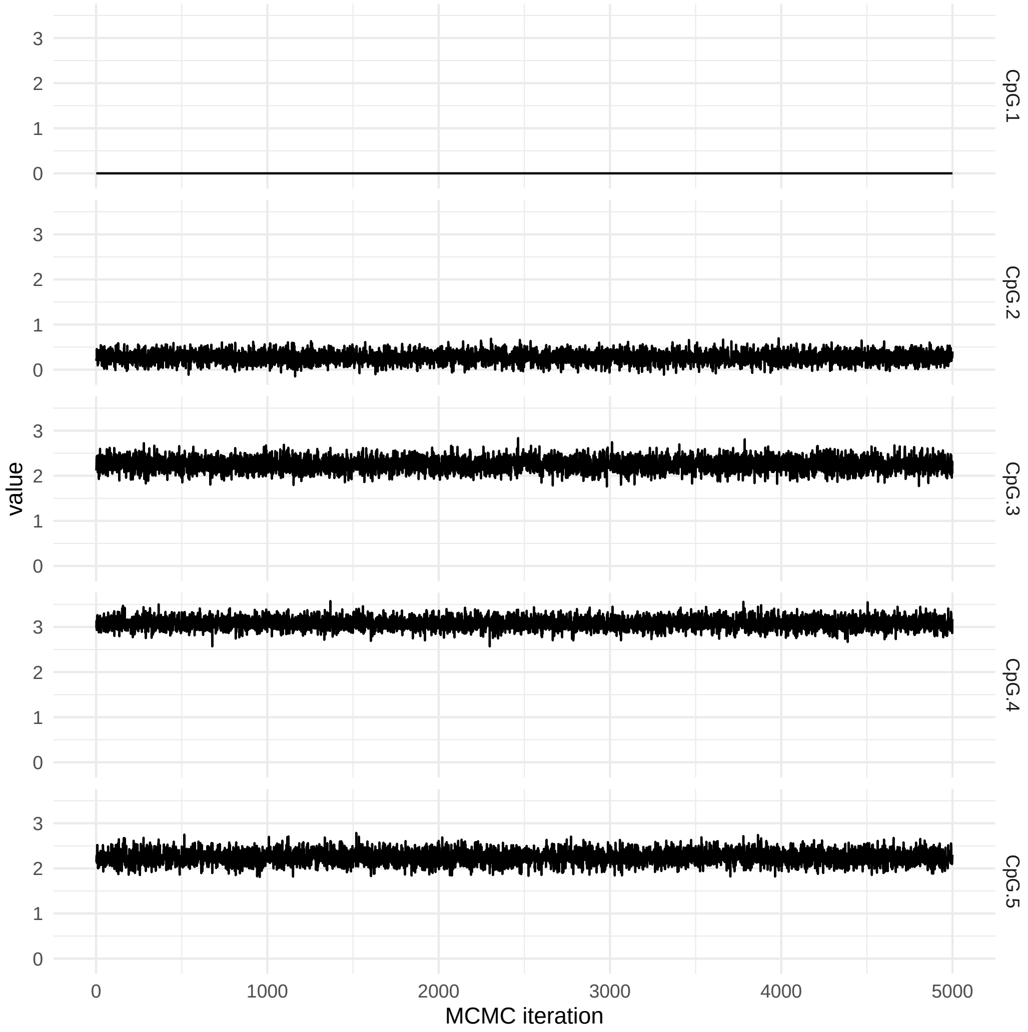

Figure S45. Trace plot of MCMC samples for a selected parameter of the model for five different genomic positions using `DMCFB`; Chromosome 17.

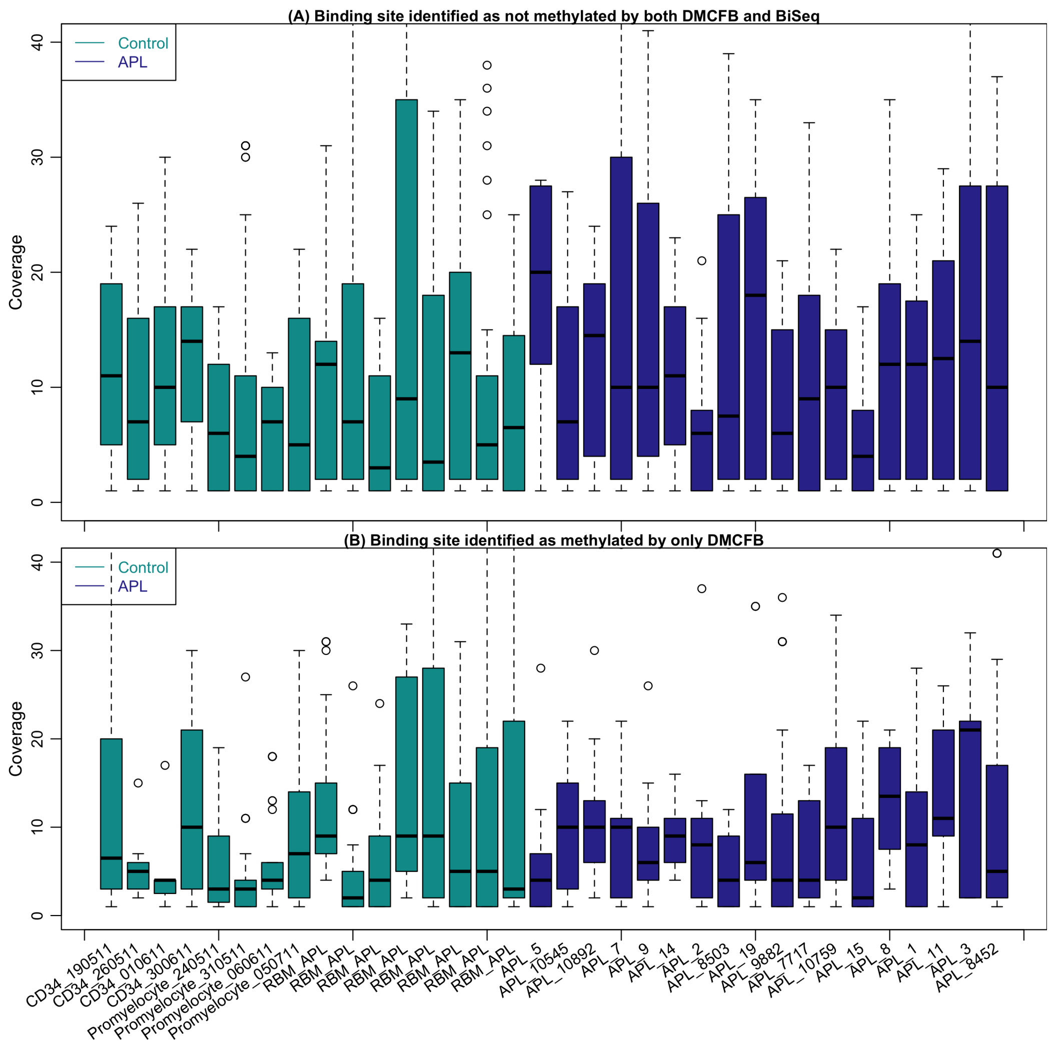

Figure S46. (A) Box-plots of read-depth of CpG positions in known binding sites identified as not DMCs by DMCFB and BiSeq; (B) Box-plots of read-depth of CpG positions in known binding sites identified as DMCs by only DMCFB and not BiSeq. Chromosome 15.

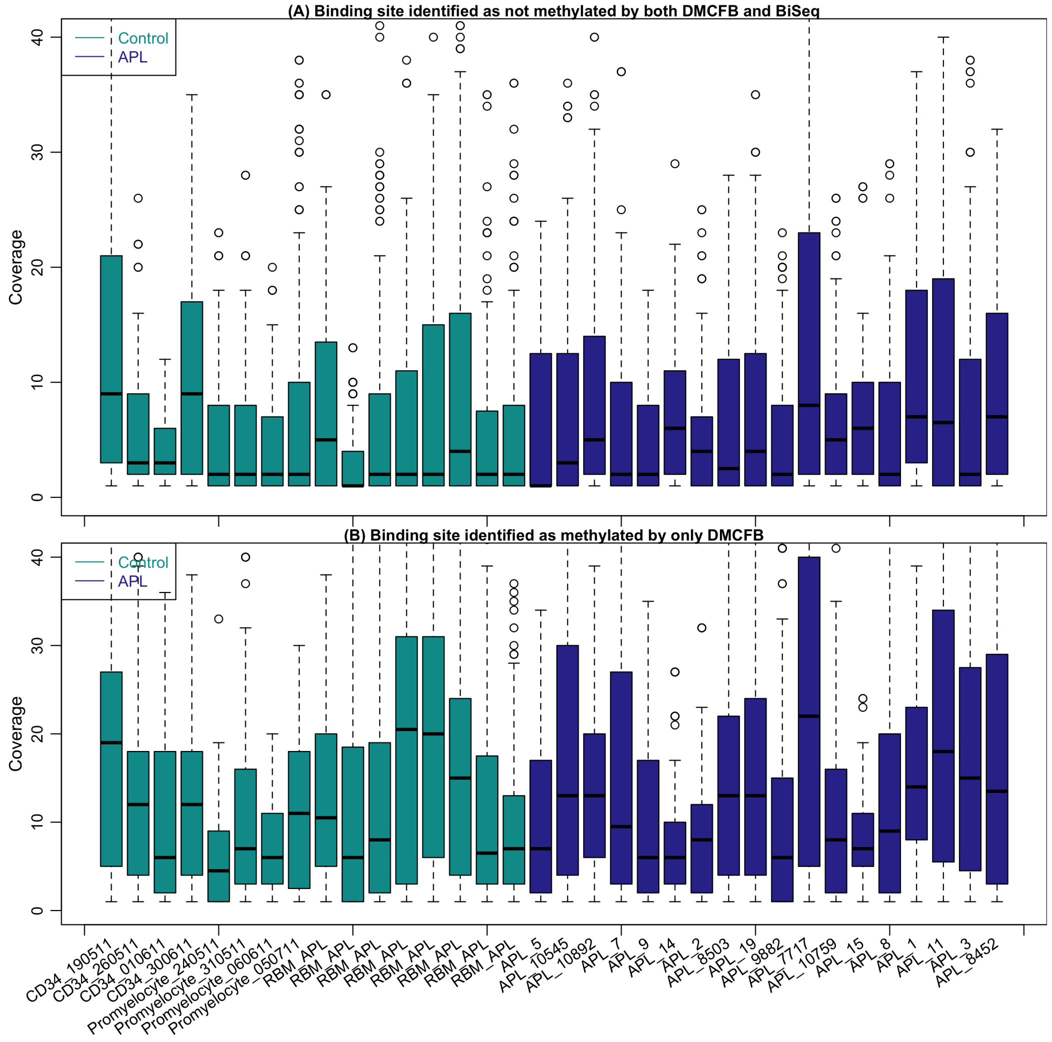

Figure S47. (A) Box-plots of read-depth of CpG positions in known binding sites identified as not DMCs by `DMCFB` and `BiSeq`; (B) Box-plots of read-depth of CpG positions in known binding sites identified as DMCs by only `DMCFB` and not `BiSeq`. Chromosome 17.

Table S9. Counts of Shore regions are shown, categorized by whether the adjacent Island contains at least one DMC and whether the Shore (in the same direction) contains at least one DMC. Counts are shown DMCFB and BiSeq; Chromosomes 15 and 17.

	# of Shores			With DMC	
Chromosome	DMCFB	BiSeq	Stream	Island	Shore
15	324	64	Up	✓	✓
	307	78	Down	✓	✓
	239	154	Up	✓	✗
	256	140	Down	✓	✗
	38	24	Up	✗	✓
	49	30	Down	✗	✓
	191	550	Up	✗	✗
	180	544	Down	✗	✗
17	770	40	Up	✓	✓
	740	53	Down	✓	✓
	443	239	Up	✓	✗
	410	226	Down	✓	✗
	170	34	Up	✗	✓
	147	33	Down	✗	✓
	314	1321	Up	✗	✗
	337	1322	Down	✗	✗

Table S10. The number of CpGs identified as DMC and NDMC by `DMCFB` versus `BiSeq`, classified by CpG Islands, Shores and Deserts, and by the direction of methylation; Chromosomes 15 and 17.

			Chromosome		15			17		
DMCFB	BiSeq	Type	Direction DMCFB	BiSeq	Equal	Hyper	Hypo	Equal	Hyper	Hypo
DMC	DMC	Island	Equal		0	0	0	0	0	0
			Hyper		0	10142	2	0	10910	0
			Hypo		0	205	167	0	59	291
		Shore	Equal		0	0	0	0	0	0
			Hyper		0	2036	9	0	2606	0
			Hypo		0	11	102	0	0	420
		Desert	Equal		0	0	0	0	0	0
			Hyper		0	1047	5	0	644	0
			Hypo		0	10	356	0	8	184
	NDMC	Island	Equal		0	0	0	0	0	0
			Hyper		5690	0	0	13146	0	0
			Hypo		21545	0	0	37337	0	0
		Shore	Equal		0	0	0	0	0	0
			Hyper		5650	0	0	18945	0	0
			Hypo		3483	0	0	10146	0	0
		Desert	Equal		0	0	0	0	0	0
			Hyper		31327	0	0	55790	0	0
			Hypo		5675	0	0	12323	0	0
NDMC	DMC	Island	Equal		0	3225	55	0	2861	26
			Hyper		0	0	0	0	0	0
			Hypo		0	0	0	0	0	0
		Shore	Equal		0	526	88	0	485	46
			Hyper		0	0	0	0	0	0
			Hypo		0	0	0	0	0	0
		Desert	Equal		0	282	245	0	93	65
			Hyper		0	0	0	0	0	0
			Hypo		0	0	0	0	0	0
	NDMC	Island	Equal		33956	0	0	74238	0	0
			Hyper		0	0	0	0	0	0
			Hypo		0	0	0	0	0	0
		Shore	Equal		16236	0	0	53027	0	0
			Hyper		0	0	0	0	0	0
			Hypo		0	0	0	0	0	0
		Desert	Equal		143362	0	0	216724	0	0
			Hyper		0	0	0	0	0	0
			Hypo		0	0	0	0	0	0

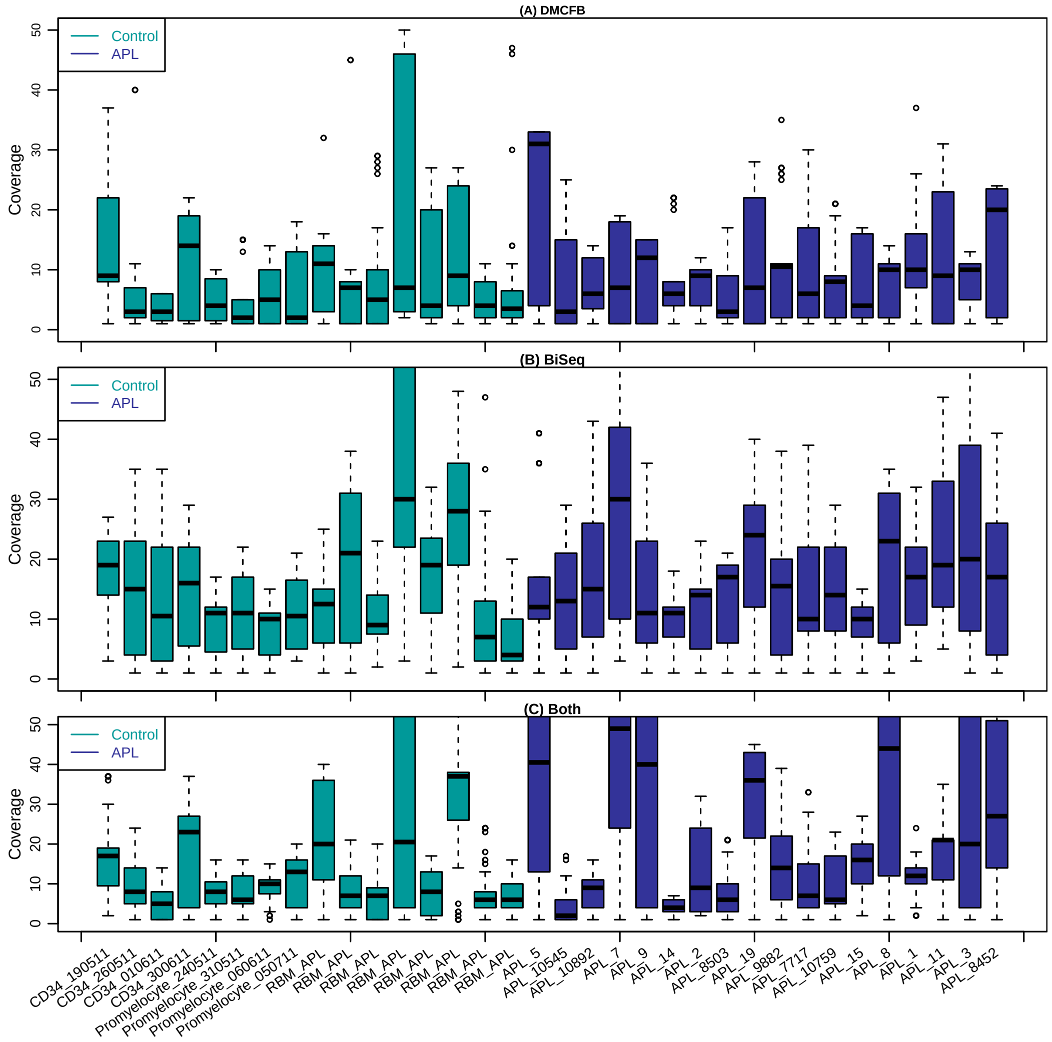

Figure S48. Box-plots of read-depth of identified DMCs by `DMCFB` (top graph), `BiSeq` (middle graph) and both (bottom graph); Chromosome 15.

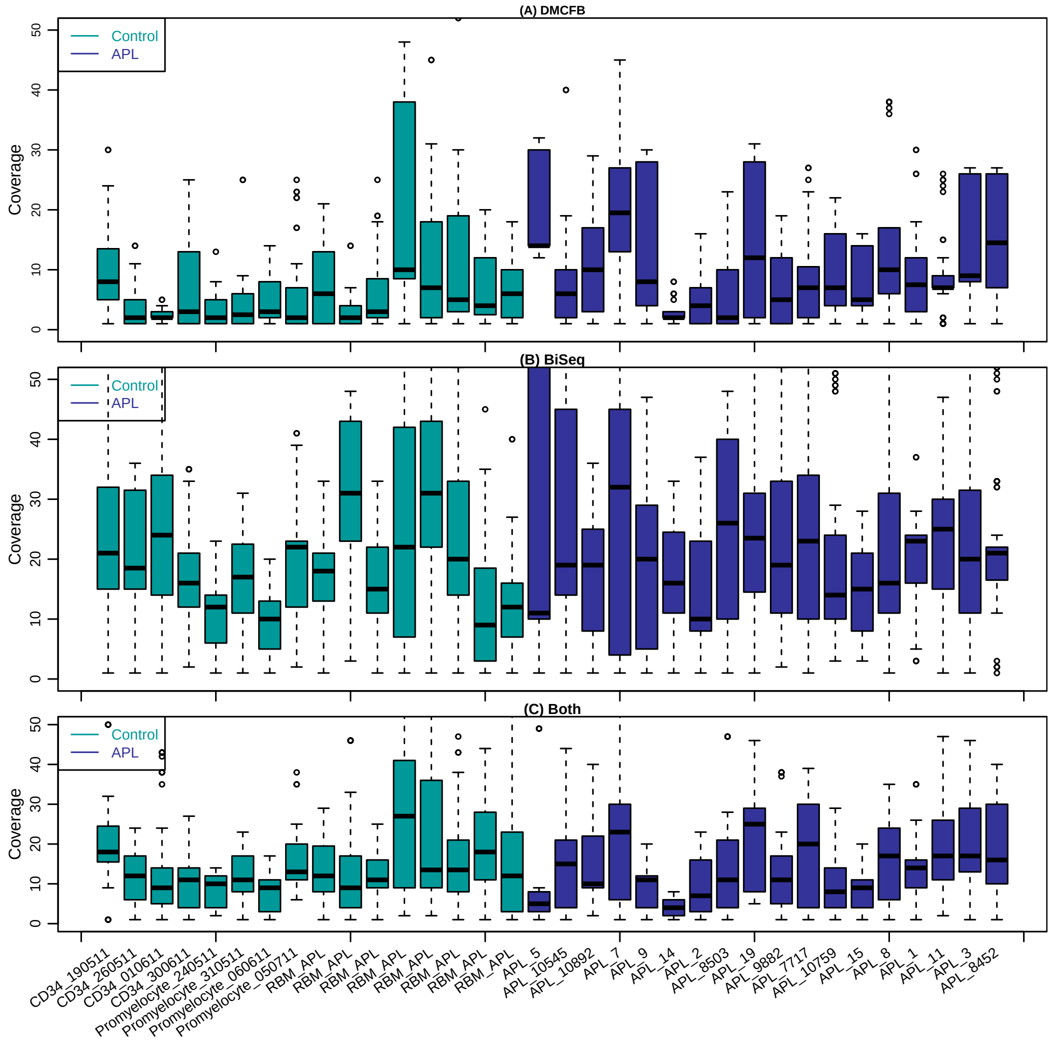

Figure S49. Box-plots of read-depth of identified DMCs by DMCFB (top graph), BiSeq (middle graph) and both (bottom graph); Chromosome 17.

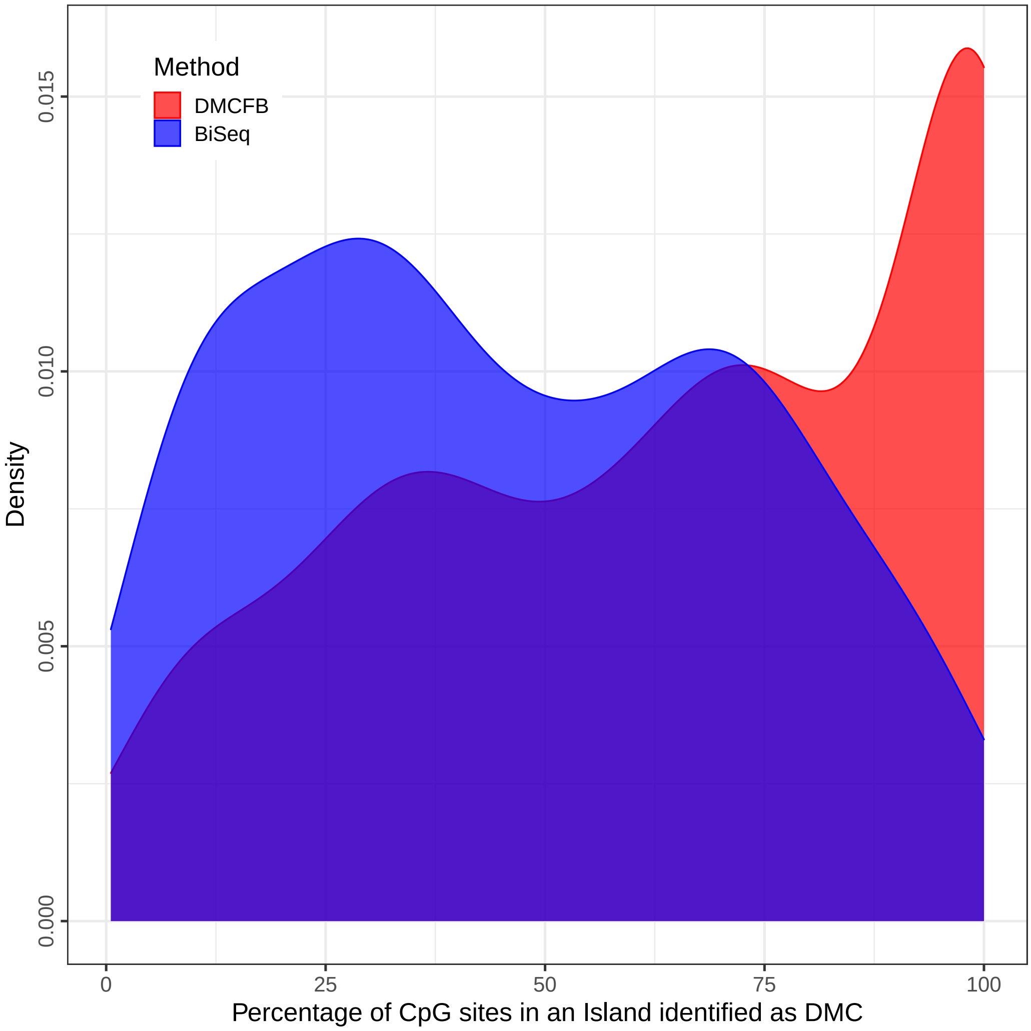

Figure S50. The density of percentage of CpGs in an Island identified as DMC by `DMCFB` (red) and `BiSeq` (blue); Chromosome 15.

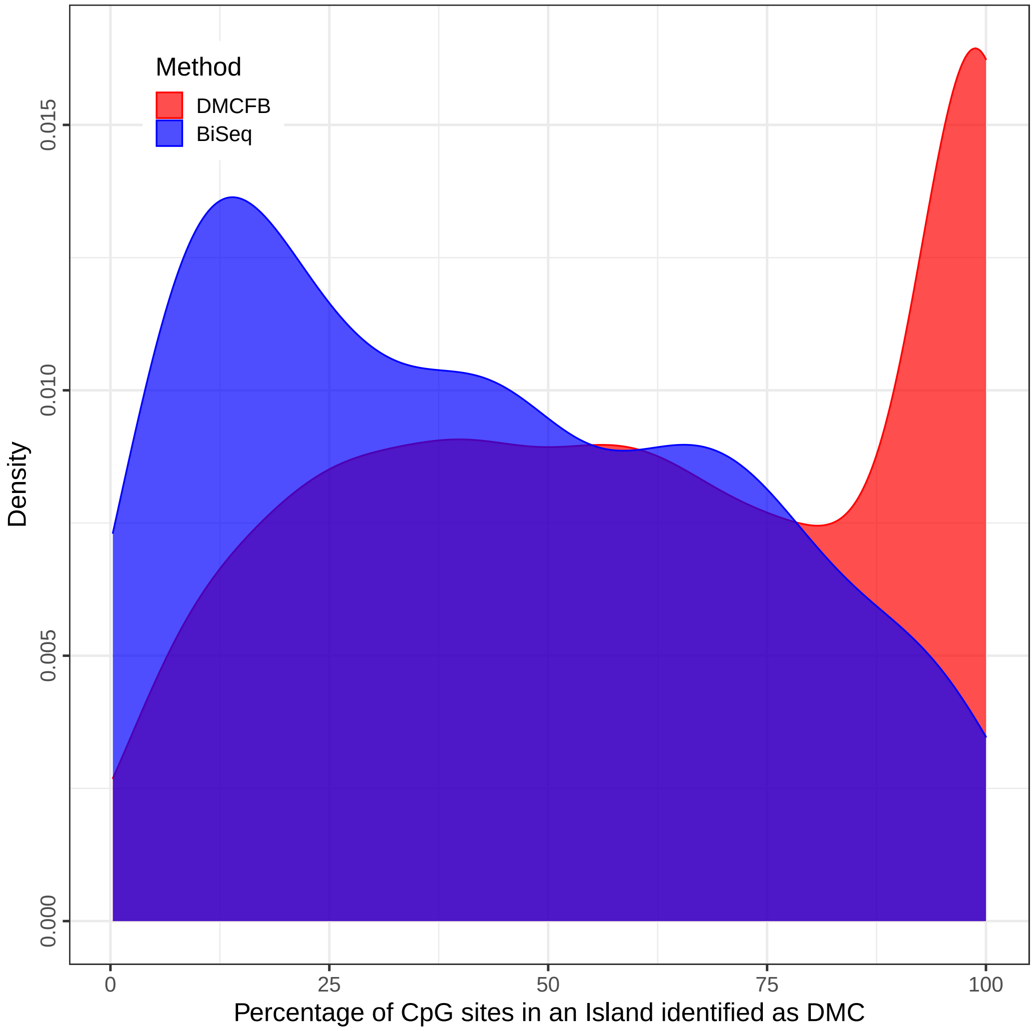

Figure S51. The density of percentage of CpGs in an Island identified as DMC by `DMCFB` (red) and `BiSeq` (blue); Chromosome 17.

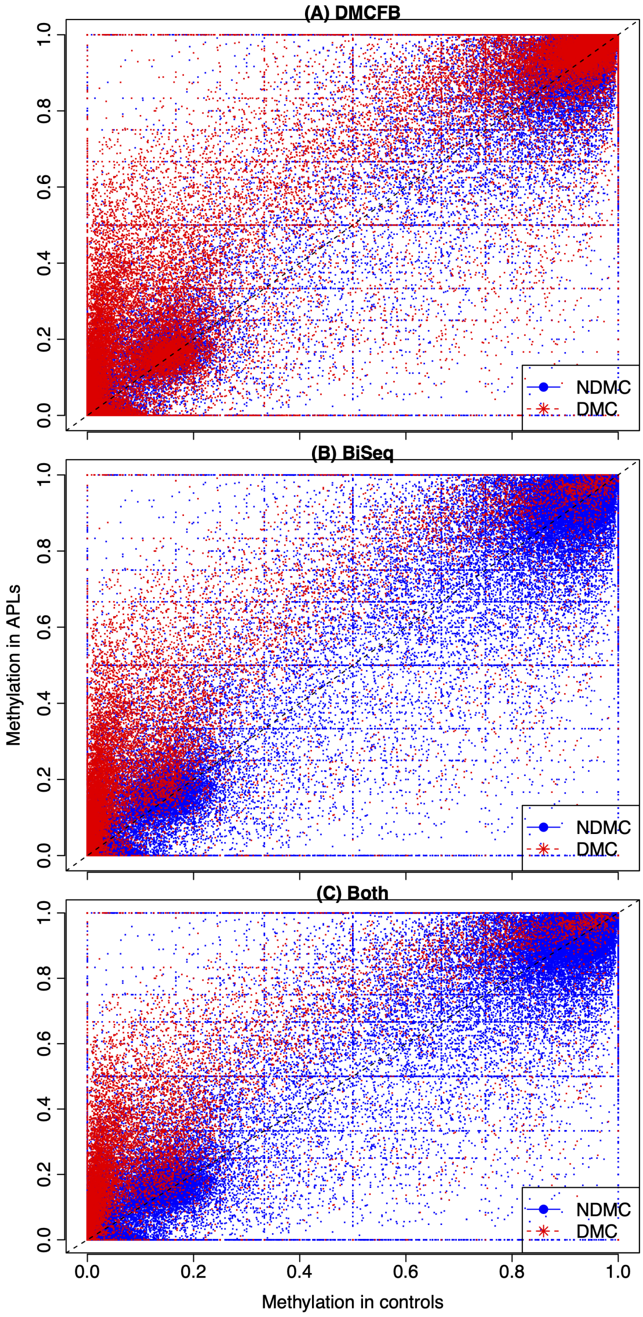

Figure S52. Raw methylation levels of CpGs for the Control group versus the APL group differentiated for identified DMCs (red) and NDMCs (blue) by `DMCFB` (top graph), `BiSeq` (middle graph) and both (bottom graph); Chromosome 15.

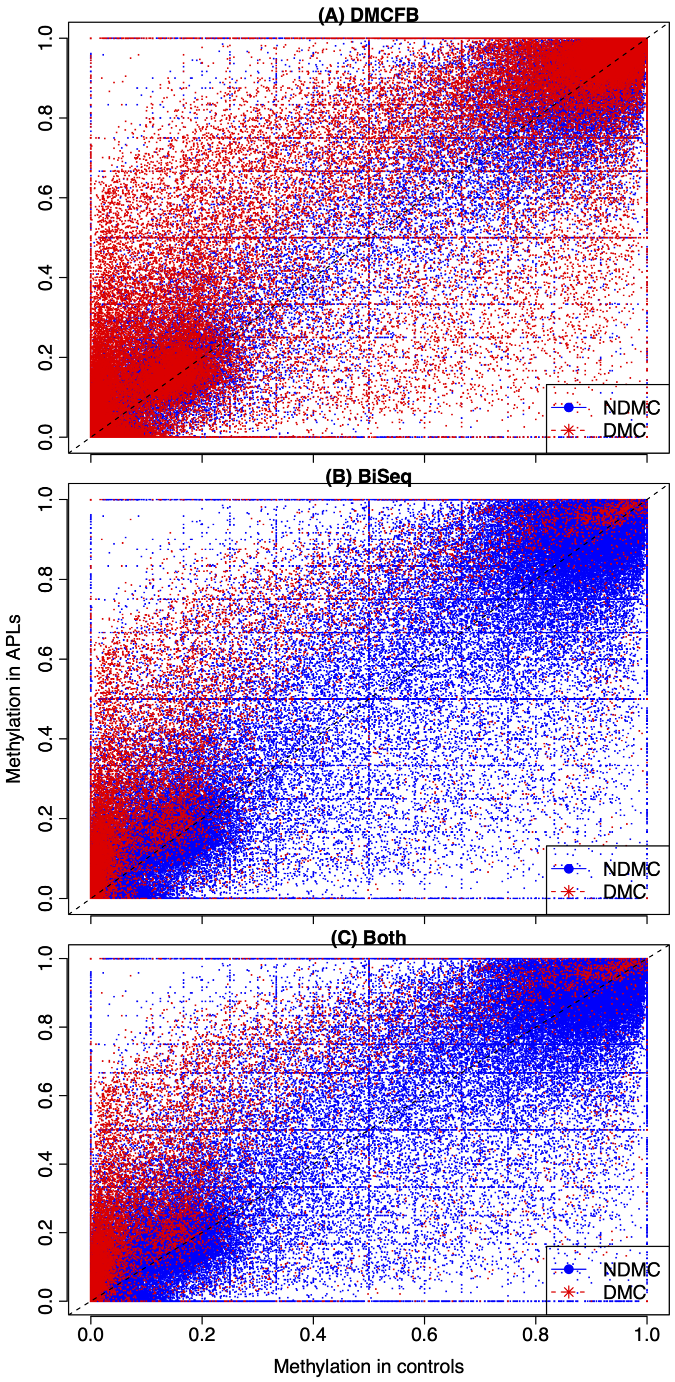

Figure S53. Raw methylation levels of CpGs for the Control group versus the APL group differentiated for identified DMCs (red) and NDMCs (blue) by `DMCFB` (top graph), `BiSeq` (middle graph) and both (bottom graph); Chromosome 17.

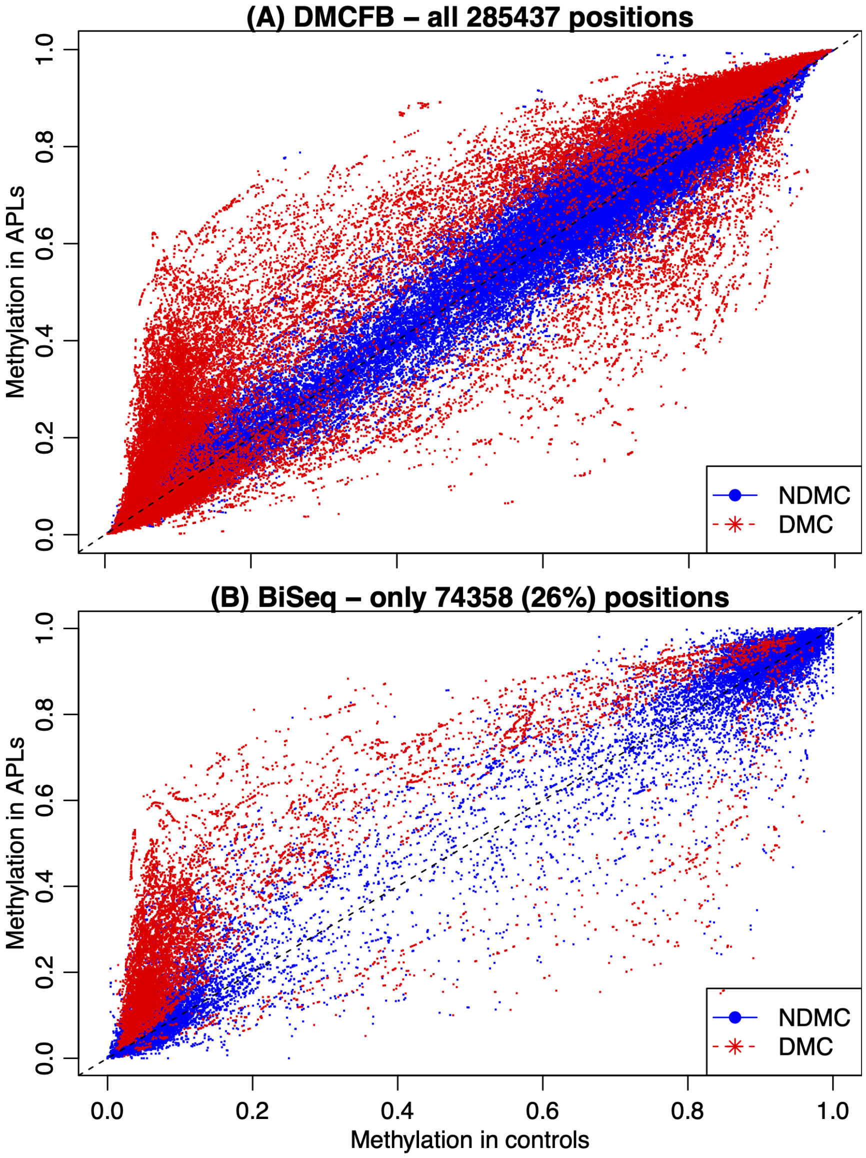

Figure S54. Predicted methylation levels of CpGs for the Control group versus the APL group differentiated for identified DMCs (red) and NDMCs (blue) by `DMCFB` (top graph) and `BiSeq` (bottom graph); Chromosome 15.

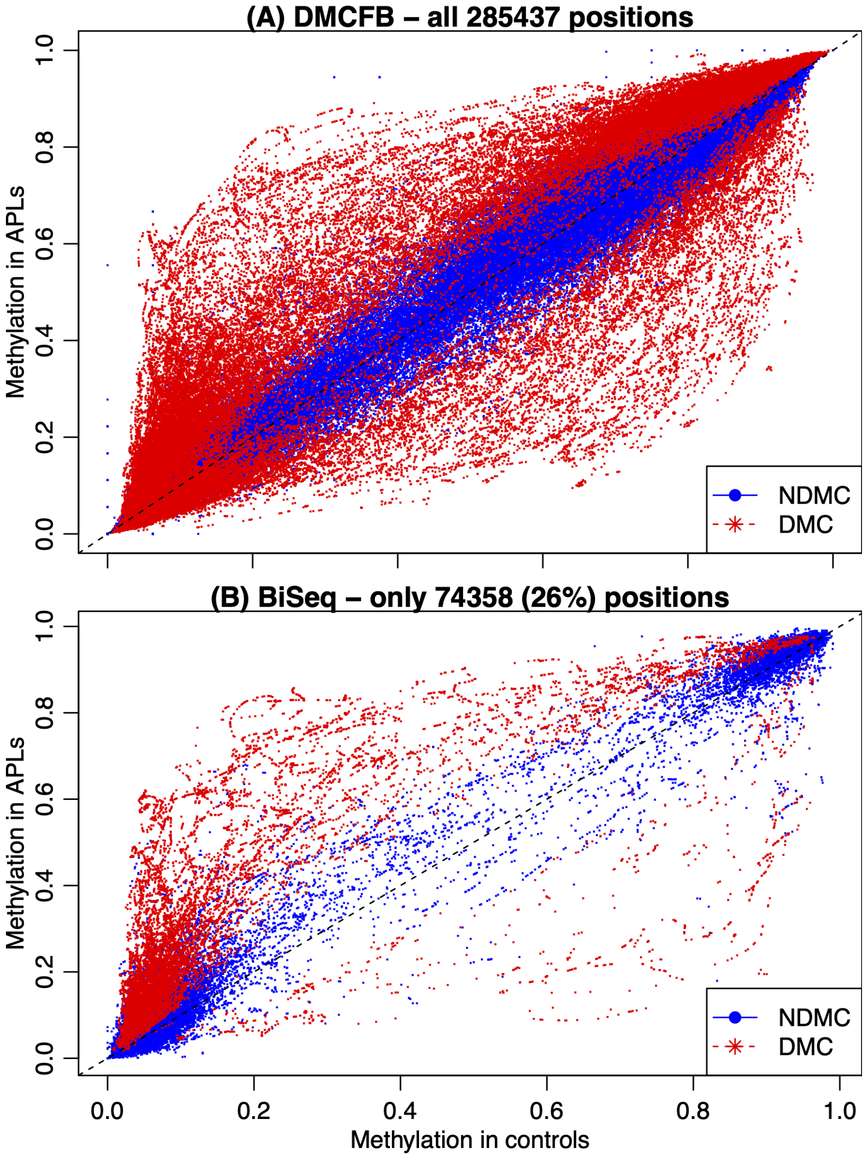

Figure S55. Predicted methylation levels of CpGs for the Control group versus the APL group differentiated for identified DMCs (red) and NDMCs (blue) by `DMCFB` (top graph) and `BiSeq` (bottom graph); Chromosome 17.

S8.2. Reanalysis of the APL data by changing the options in BiSeq

The BiSeq pipeline has different options; changing them will result in different percentages of DMCs. In this section, we have changed some of the options to increase the chance of identifying more DMRs leading to more DMCs. Table S11 presents the results. Although the percentage of DMCs has increased in both Chromosome 15 and 17, the agreement between DMCFB and BiSeq has decreased.

Table S11. Comparing DMCFB and BiSeq in identifying DMCs in the APL data (Default options in BiSeq are changed to increase the chance of identifying more DMRs.)

(A) Chromosome 15

		BiSeq					
**All CpGs**		NDMC	% of 2x2 table	% of BiSeq NDMC	DMC	% of 2x2 table	% of BiSeq DMC
DMCFB	NDMC[1]	186,941	65.49		11,034	3.87	
	DMC	68,781	24.10	26.90	18,681	6.54	62.87
**CpG Island**							
DMCFB	NDMC	28,331	37.78		8,905	11.88	
	DMC	23,378	31.18	45.21	14,373	19.17	61.74
**CpG Shore**							
DMCFB	NDMC	15,332	54.48		1,518	5.39	
	DMC	8,191	29.11	34.82	3,100	11.02	67.13
**CpG Desert**							
DMCFB	NDMC	143,278	78.59		611	0.34	
	DMC	37,212	20.41	20.62	1,208	0.66	66.41

(B) Chromosome 17

		BiSeq					
**All CpGs**		NDMC	% of 2x2 table	% of BiSeq NDMC	DMC	% of 2x2 table	% of BiSeq DMC
DMCFB	NDMC	332,734	65.19		14,831	2.91	
	DMC	135,692	26.59	28.97	27,129	5.32	64.65
**CpG Island**							
DMCFB	NDMC	66,093	47.59		11,032	7.94	
	DMC	44,655	32.16	40.32	17,088	12.31	60.77
**CpG Shore**							
DMCFB	NDMC	50,819	59.32		2,739	3.20	
	DMC	25,588	29.87	33.49	6,529	7.62	70.45
**CpG Desert**							
DMCFB	NDMC	215,822	75.50		1,060	0.37	
	DMC	65,449	22.90	23.27	3,512	1.23	76.82

[1] Not a DMC

S8.3. Reanalysis of the APL *data using* `DMCHMM`

In this section, we analyze the APL data using DMCHMM and compare the results with those in Table 1(A and B) The statistical model considered in DMCHMM is an HMM with

$$
\begin{array}{lll}
\text{Initial probability} & : & p(X_1 = k) = \pi_k, \quad k \in \{0, \ldots, K+1\}, \\
\text{Transition probability} & : & p(X_t = k | X_{t-1} = k') = p_{k',k}, \quad k', k \in \{0, \ldots, K+1\}, \quad X_0 \equiv X_1 \\
\text{Emission probability} & : & Y_t | X_t = k \overset{iid}{\sim} \text{Bin}(n_t, \beta_k), \quad k \in \{0, \ldots, K+1\},
\end{array}
$$

in the first and second step, the Bayesian model

$$p(\boldsymbol{y}|\boldsymbol{x};\boldsymbol{\beta})p(\boldsymbol{\beta};\boldsymbol{x},\boldsymbol{z},\gamma)p(\boldsymbol{x};\boldsymbol{\pi},\boldsymbol{P})$$

in the second step, and the logistic model

$$\log\{\beta_{ti}/(1-\beta_{ti})\} = \boldsymbol{Z}_i\gamma_t + \epsilon_{ti}, \quad i = 1, \ldots, 34, \tag{1}$$

in the third step, where $\boldsymbol{Z}_i$ is a vector of categorical variables that represents grouping information (i.e., Control and APL), and $\epsilon_{t,i}$ is a random variable term representing incidental variation for sample i at position t. Note that $t = 1, \ldots, T$, where $T = 285,437$ and $510,386$ for Chromosome 15 and 17, respectively

Table S12. The percentage of CpGs identified as DMC by DMCFB and DMCHMM displayed; Chromosomes 15 and 17.

Chromosome	DMCFB	DMCHMM
15	30.64	17.36
17	31.90	9.43

Table S13. The number of CpGs identified as DMC by only DMCFB, only DMCHMM and both methods at the same time, separated for CpG Islands and Shores; Chromosomes 15 and 17.

Chromosome	Location	DMCFB	DMCHMM	Both
15	Islands	563	764	563
	Shores	718	1452	718
17	Islands	1150	1574	1150
	Shores	1764	3183	1764

Table S14. Counts of Shore regions are shown, categorized by whether the adjacent Island contains at least one DMC and whether the Shore (in the same direction) contains at least one DMC. Counts are shown DMCFB and DMCHMM; Chromosomes 15 and 17.

	# of Shores			With DMC	
Chromosome	DMCFB	DMCHMM	Stream	Island	Shore
15	324	702	Up	✓	✓
	307	709	Down	✓	✓
	239	62	Up	✓	✗
	256	55	Down	✓	✗
	38	21	Up	✗	✓
	49	20	Down	✗	✓
	191	7	Up	✗	✗
	180	8	Down	✗	✗
17	707	1535	Up	✓	✓
	740	1541	Down	✓	✓
	443	39	Up	✓	✗
	410	33	Down	✓	✗
	170	52	Up	✗	✓
	147	55	Down	✗	✓
	314	8	Up	✗	✗
	337	5	Down	✗	✗

Table S15. The number of CpGs identified as DMC and NDMC by DMCFB versus DMCHMM, classified by CpG Islands, Shores and Deserts, and by the direction of methylation; Chromosomes 15 and 17.

			Chromosome	15			17		
			Direction DMCHMM	Equal	Hyper	Hypo	Equal	Hyper	Hypo
DMCFB	DMCHMM	Type	DMCFB						
DMC	DMC	Island	Equal	0	0	0	0	0	0
			Hyper	0	6954	0	0	4259	0
			Hypo	0	1922	1	0	1660	63
		Shore	Equal	0	0	0	0	0	0
			Hyper	0	2751	0	0	2769	7
			Hypo	0	507	0	0	349	255
		Desert	Equal	0	0	0	0	0	0
			Hyper	0	5268	21	0	5765	27
			Hypo	0	723	0	0	1167	168
	NDMC	Island	Equal	0	0	0	0	0	0
			Hyper	75	7570	1235	118	15819	3860
			Hypo	204	13925	5866	435	23220	12309
		Shore	Equal	0	0	0	0	0	0
			Hyper	11	4020	913	149	13981	4645
			Hypo	39	1801	1249	85	5846	4031
		Desert	Equal	0	0	0	0	0	0
			Hyper	260	20584	6246	691	33018	16953
			Hypo	86	3211	2021	94	5126	5952
NDMC	DMC	Island	Equal	0	5048	11	0	6192	12
			Hyper	0	0	0	0	0	0
			Hypo	0	0	0	0	0	0
		Shore	Equal	0	3125	141	0	4881	48
			Hyper	0	0	0	0	0	0
			Hypo	0	0	0	0	0	0
		Desert	Equal	0	23038	36	0	20107	381
			Hyper	0	0	0	0	0	0
			Hypo	0	0	0	0	0	0
	NDMC	Island	Equal	393	23966	7818	907	49284	20730
			Hyper	0	0	0	0	0	0
			Hypo	0	0	0	0	0	0
		Shore	Equal	120	9451	4013	503	32751	15375
			Hyper	0	0	0	0	0	0
			Hypo	0	0	0	0	0	0
		Desert	Equal	1375	88534	30906	2686	120235	73473
			Hyper	0	0	0	0	0	0
			Hypo	0	0	0	0	0	0

Table S16. Comparing DMCFB and DMCHMM in identifying DMCs in the APL data.

(A) Chromosome 15

		DMCHMM					
**All CpGs**		NDMC	% of 2x2 table	% of DMCHMM NDMC	DMC	% of 2x2 table	% of DMCHMM DMC
DMCFB	NDMC	166,576	58.36		31,399	11.00	
	DMC	69,316	24.28	29.38	18,146	6.36	36.63
**CpG Island**							
DMCFB	NDMC	32,177	42.91		5,059	6.75	
	DMC	28,875	38.51	47.30	8,876	11.84	63.70
**CpG Shore**							
DMCFB	NDMC	13,584	48.27		3,266	11.61	
	DMC	8,033	28.55	37.16	3,258	11.58	49.94
**CpG Desert**							
DMCFB	NDMC	120,815	66.27		23,074	12.66	
	DMC	32,408	17.78	21.15	6,012	3.30	20.67

(B) Chromosome 17

		DMCHMM					
**All CpGs**		NDMC	% of 2x2 table	% of DMCHMM NDMC	DMC	% of 2x2 table	% of DMCHMM DMC
DMCFB	NDMC	315,944	61.90		31,621	6.20	
	DMC	146,332	28.67	31.65	16,489	3.23	34.27
**CpG Island**							
DMCFB	NDMC	70,921	51.07		6,204	4.47	
	DMC	55,761	40.15	44.02	5,982	4.31	49.09
**CpG Shore**							
DMCFB	NDMC	48,629	56.76		4,929	5.76	
	DMC	28,737	33.54	41.69	3,380	3.95	40.68
**CpG Desert**							
DMCFB	NDMC	196,394	68.71		20,488	7.17	
	DMC	61,834	21.63	23.95	7,127	2.49	25.81

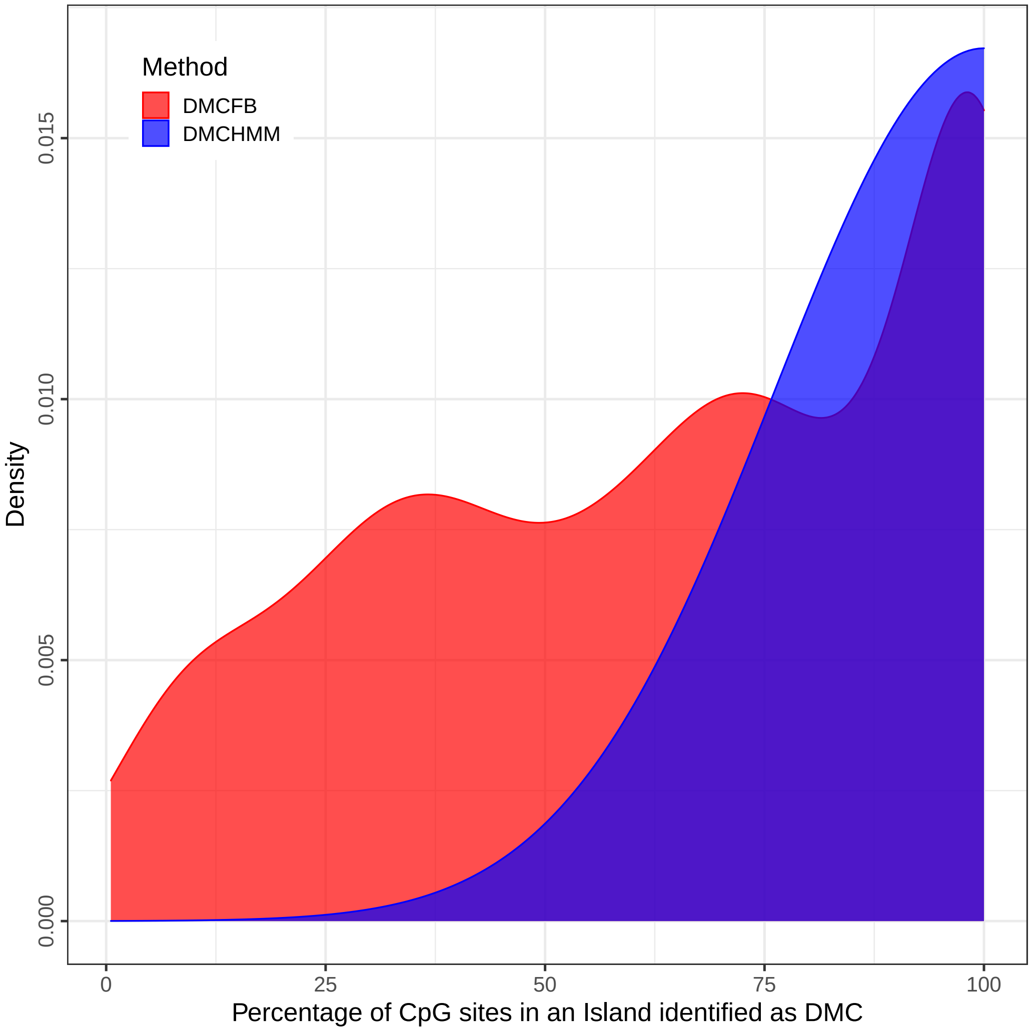

Figure S56. The density of percentage of CpGs in an Island identified as DMC by `DMCFB` (red) and `DMCHMM` (blue); Chromosome 15.

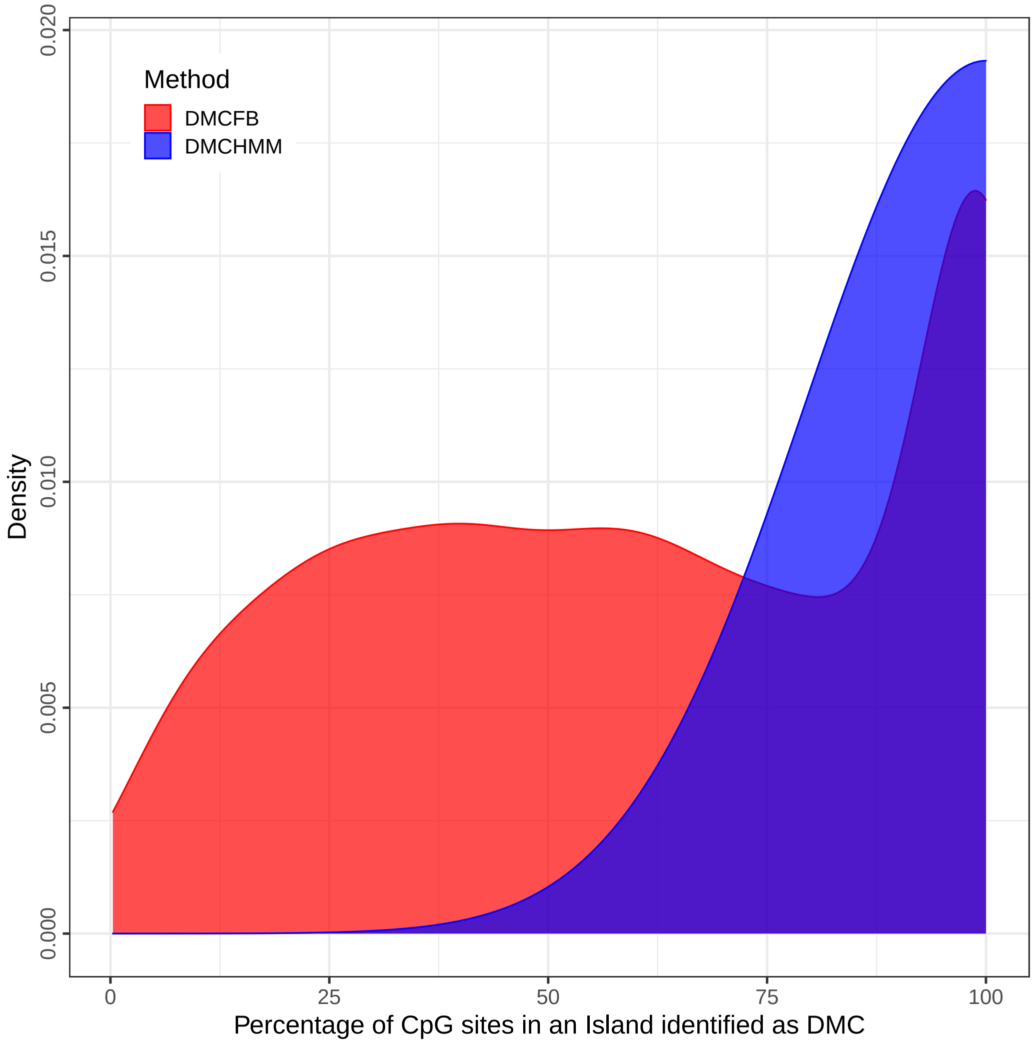

Figure S57. The density of percentage of CpGs in an Island identified as DMC by DMCFB (red) and DMCHMM (blue); Chromosome 17.

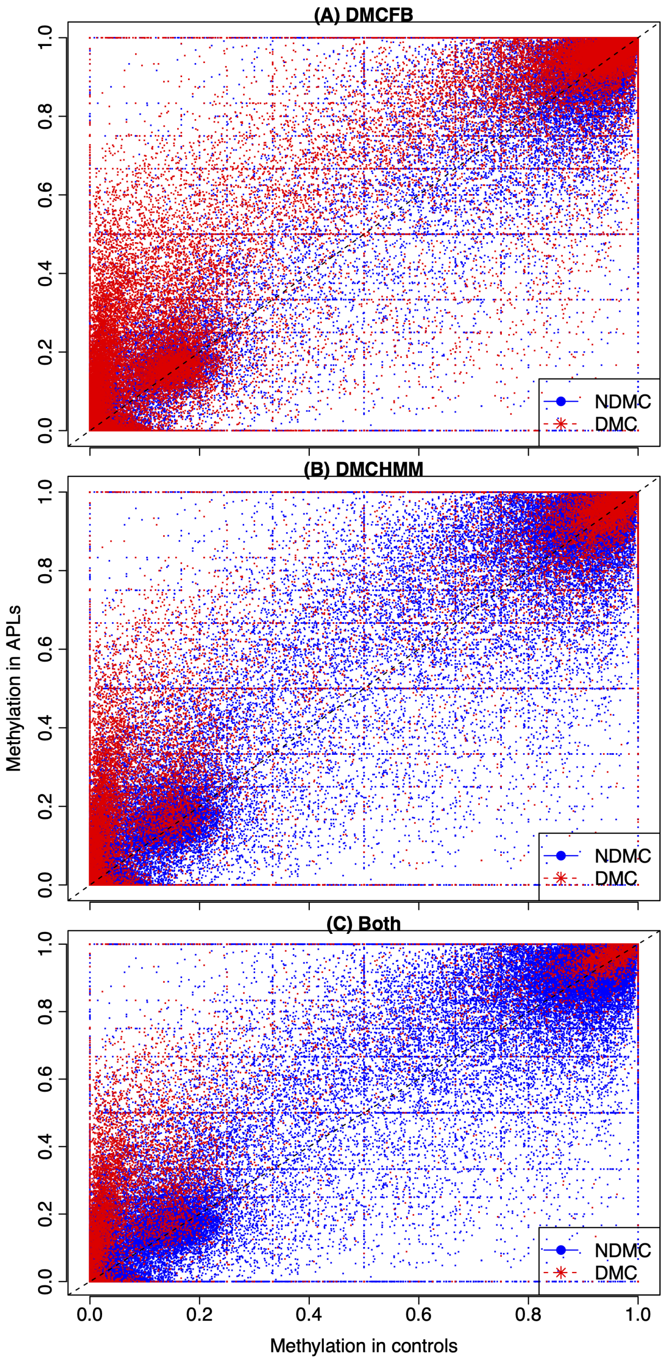

Figure S58. Raw methylation levels of CpGs for the Control group versus the APL group differentiated for identified DMCs (red) and NDMCs (blue) by DMCFB (top graph), DMCHMM (middle graph) and both (bottom graph); Chromosome 15.

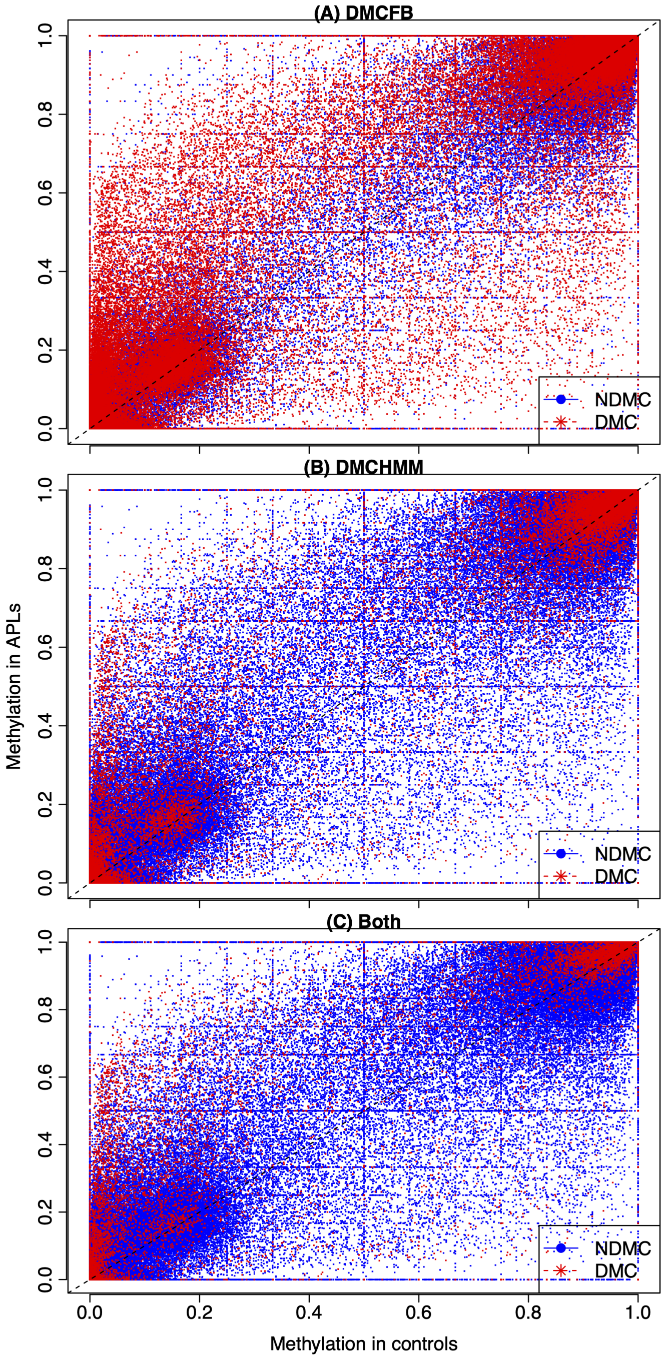

Figure S59. Raw methylation levels of CpGs for the Control group versus the APL group differentiated for identified DMCs (red) and NDMCs (blue) by DMCFB (top graph), DMCHMM (middle graph) and both (bottom graph); Chromosome 17.

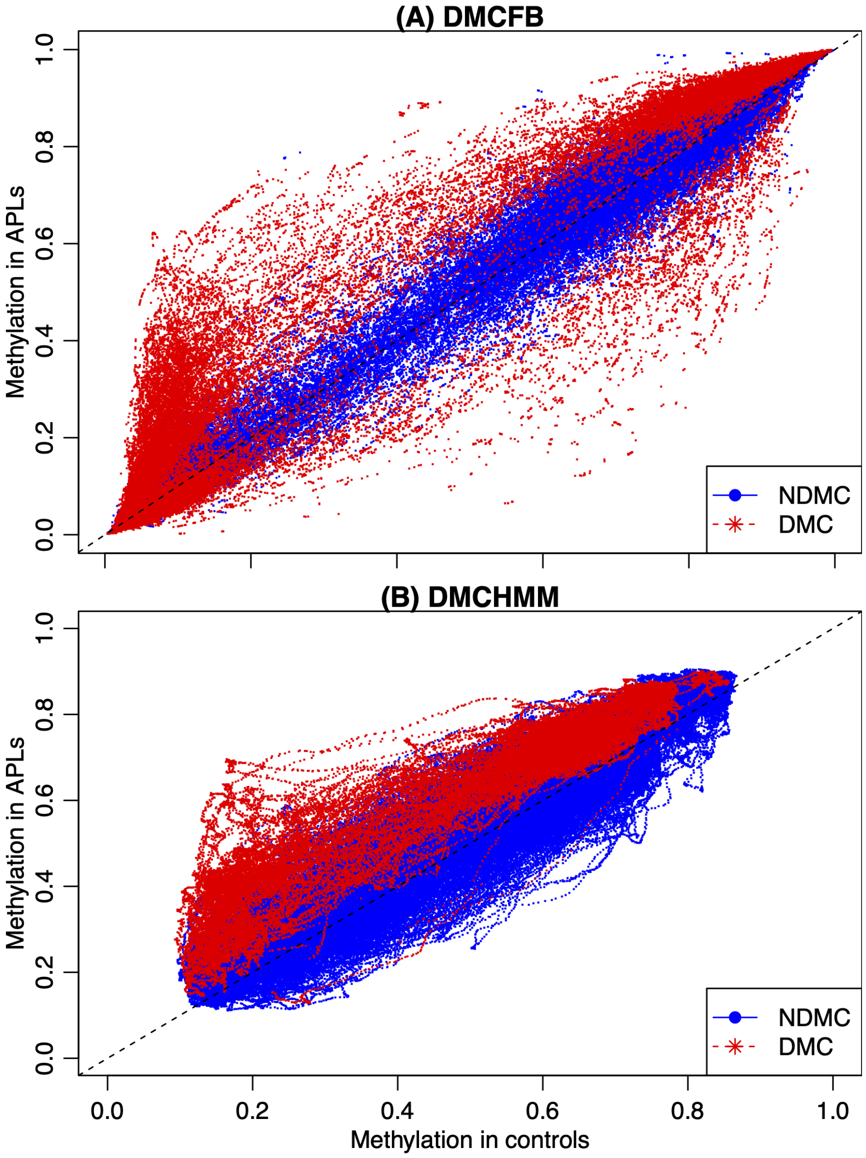

Figure S60. Predicted methylation levels of CpGs for the Control group versus the APL group differentiated for identified DMCs (red) and NDMCs (blue) by `DMCFB` (top graph) and `DMCHMM` (bottom graph); Chromosome 15.

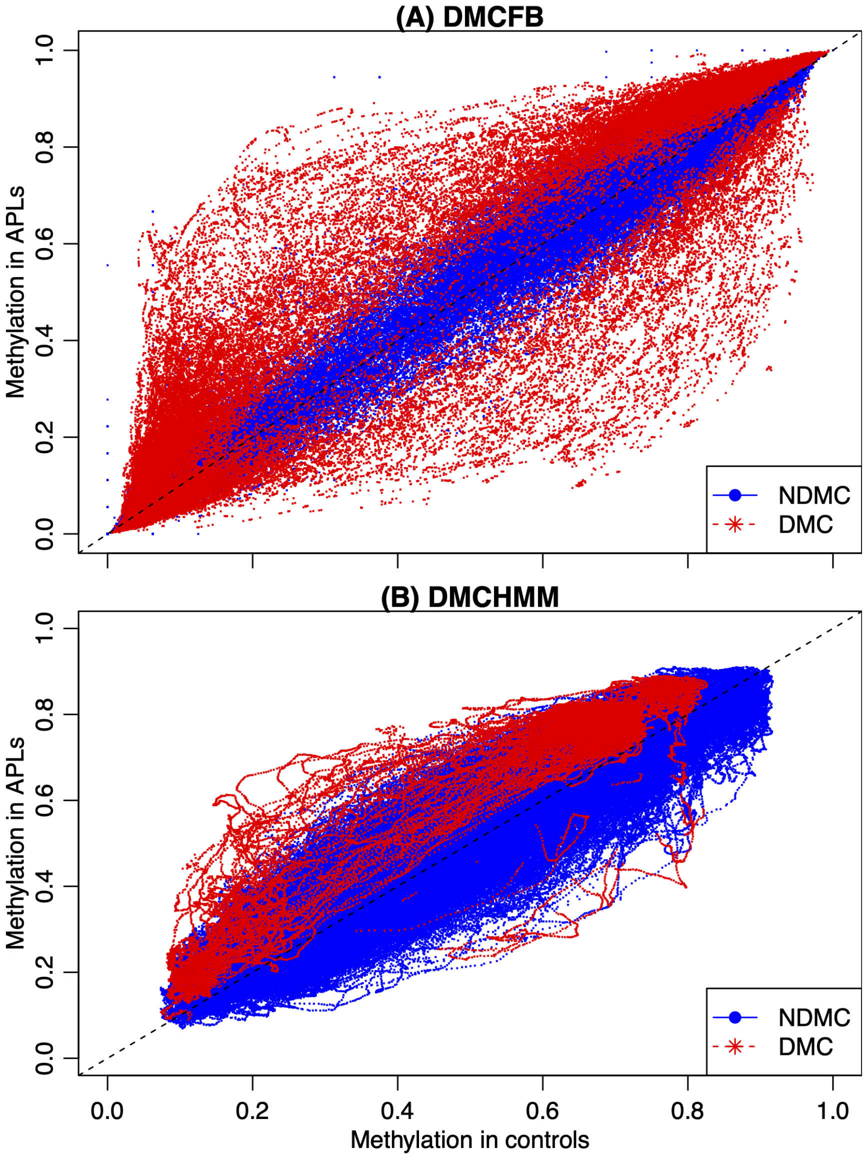

Figure S61. Predicted methylation levels of CpGs for the Control group versus the APL group differentiated for identified DMCs (red) and NDMCs (blue) by DMCFB (top graph) and DMCHMM (bottom graph); Chromosome 17.

References

1. Cheung, A.; Shao, X.; Morin, A.; Siroux, V.; et al. Functional variation in allelic methylomes underscores a strong genetic contribution and reveals novel epigenetic alterations in the human epigenome. *Genome Biology* **2017**, *18*, 50.
2. Shokoohi, F.; Stephens, D.A.; Bourque, G.; Pastinen, T.; Greenwood, C.M.T.; Labbe, A. A hidden Markov model for identifying differentially methylated sites in bisulfite sequencing data. *Biometrics* **2019**, *75*, 210–221. https://doi.org/10.1111/biom.12965.
3. Schoofs, T.; Rohde, C.; Hebestreit, K.; Klein, H.; Göllner, S.; Schulze, I.; et al. DNA methylation changes are a late event in acute promyelocytic leukemia and coincide with loss of transcription factor binding. *Blood* **2013**, *121*, 178.
4. Hebestreit, K.; Dugas, M.; Klein, H.U. Detection of significantly differentially methylated regions in targeted bisulfite sequencing data. *Bioinformatics* **2013**, *29*, 1647–1653.
5. Song, Q.; Decato, B.; Hong, E.E.; Zhou, M.; Fang, F.; Qu, J.; et al. A Reference Methylome Database and Analysis Pipeline to Facilitate Integrative and Comparative Epigenomics. *PLOS ONE* **2013**, *8*, e81148.
6. Kishore, K.; de Pretis, S.; Lister, R.; Morelli, M.; et al. methylPipe and compEpiTools: a suite of R packages for the integrative analysis of epigenomics data. *BMC Bioinformatics* **2015**. https://doi.org/10.1186/s12859-015-0742-6.
7. Akalin, A.; Kormaksson, M.; Li, S.; Garrett-Bakelman, F.E.; Figueroa, M.E.; Melnick, A.; et al. methylKit: a comprehensive R package for the analysis of genome-wide DNA methylation profiles. *Genome Biology* **2012**, *13*, R87.
8. Saito, Y.; Mituyama, T. Detection of differentially methylated regions from bisulfite-seq data by hidden Markov models incorporating genome-wide methylation level distributions. *BMC Genomics* **2015**, *16*, S3.
9. Klein, H.U.; Hebestreit, K. An evaluation of methods to test predefined genomic regions for differential methylation in bisulfite sequencing data. *Briefings in Bioinformatics* **2015**, *17*, 796–807.
10. Wu, H.; Xu, T.; Feng, H.; Chen, L.; Li, B.; Yao, B.; et al. Detection of differentially methylated regions from whole-genome bisulfite sequencing data without replicates. *Nucleic Acids Research* **2015**, *43*, e141.
11. Yu, X.; Sun, S. Comparing five statistical methods of differential methylation identification using bisulfite sequencing data. *Statistical Applications in Genetics and Molecular Biology* **2016**, *15*, 173–191.
12. Coombs, C.C.; Tavakkoli, M.; Tallman, M.S. Acute promyelocytic leukemia: where did we start, where are we now, and the future. *Blood Cancer Journal* **2015**, *5*, e304.

Disclaimer/Publisher's Note: The statements, opinions and data contained in all publications are solely those of the individual author(s) and contributor(s) and not of MDPI and/or the editor(s). MDPI and/or the editor(s) disclaim responsibility for any injury to people or property resulting from any ideas, methods, instructions or products referred to in the content.
